# Regioselective quinazoline C2 modifications through the azide–tetrazole tautomeric equilibrium

**DOI:** 10.3762/bjoc.20.61

**Published:** 2024-03-28

**Authors:** Dāgs Dāvis Līpiņš, Andris Jeminejs, Una Ušacka, Anatoly Mishnev, Māris Turks, Irina Novosjolova

**Affiliations:** 1 Faculty of Natural Sciences and Technology, Riga Technical University, P. Valdena Str. 3, Riga, LV-1048, Latvia; 2 Ķekava Secondary School, Gaismas Str. 9, Ķekava, Ķekava Parish, Ķekava Municipality, LV-2123, Latvia; 3 Latvian Institute of Organic Synthesis, Aizkraukles Str. 21, Riga, LV-1006, Latvia

**Keywords:** aromatic nucleophilic substitution, azide–tetrazole equilibrium, 4-azido-2-sulfonylquinazolines, quinazolines, sulfonyl group dance

## Abstract

2-Chloro-4-sulfonylquinazolines undergo functional group swap when treated with an azide nucleophile: 1) the azide replaces the sulfonyl group at the C4 position; 2) the intrinsic azide–tetrazole tautomeric equilibrium directs the nucleofugal sulfinate from the first step to replace chloride at the C2 position. This transformation is effective with quinazolines bearing electron-rich substituents. Therefore, the title transformations are demonstrated on the 6,7-dimethoxyquinazoline core, which is present in pharmaceutically active substances. The methodology application is showcased by transforming the obtained 4-azido-6,7-dimethoxy-2-sulfonylquinazolines into the α_1_-adrenoceptor blockers terazosin and prazosin by further C2-selective S_N_Ar reaction and azide reduction.

## Introduction

The quinazoline core is a privileged structure with a wide range of applications. Quinazoline derivatives exhibit a broad spectrum of biological activities, finding use as anticancer, antimicrobial, antimalarial, and antiviral agents [1–2]. Furthermore, numerous 2-amino-6,7-dimethoxyquinazoline analogs are extensively employed as α_1_-adrenoceptor blockers [3–4]. In recent years quinazoline-based OLED materials have also gained attention showing great quantum efficiencies [5–7]. Consequently, ongoing efforts focus on advancing methodologies for synthesizing established quinazoline-based drugs and acquiring novel modified quinazoline derivatives for pharmaceutical or materials science purposes.

**Scheme 1:**
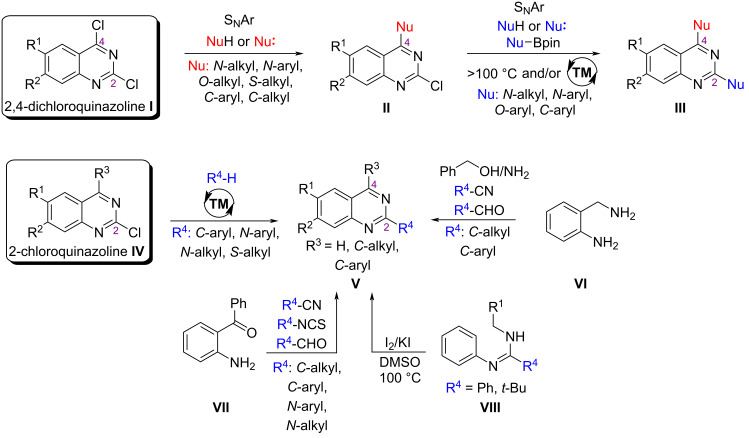
Approaches for quinazoline modifications at the C2 and C4 positions.

Aromatic nucleophilic substitution [8] or metal-catalyzed reactions [9–10] are commonly employed for quinazoline modification (Scheme 1). Existing literature underscores the reactivity of the C4 position in aromatic nucleophilic substitutions of quinazolines **I** while achieving regioselective replacement at the C2 position poses challenges [11]. Modification of the C2 position of quinazolines requires longer time, higher temperatures, and sometimes the use of expensive transition-metal catalysts [12]. A selective C2 modification can be achieved by using 2-chloroquinazolines **IV**, where the C4 position is blocked by an unreactive C–C or C–H bond (Scheme 1). Cyclization reactions of substituted anilines **VI**, **VII** or *N*-arylamidines **VIII** are frequently employed for synthesizing C2-substituted quinazolines (Scheme 1), thereby influencing the spatial arrangement of the desired substituents [13–14]. Moreover, there have been recent advancements in efficient C–H activation techniques employing transition-metal and photocatalysis [15–16]. These methods facilitate C–C bond formation, enabling the introduction of alkyl groups at the C2 position of quinazoline derivatives.

While arylsulfanyl group rearrangement reactions have been documented by us for modifying 2,4-substituted quinazolines [17–18], and sulfonyl group rearrangement has been applied to functionalize purines [19], the literature lacks information on sulfonyl group migration in quinazolines. Notably, this transformation has not been previously reported, despite its potential utility in the synthesis of drugs such as terazosin and prazosin [20].

Herein, we report the use of the sulfonyl group dance to synthesize novel 4-azido-2-sulfonylquinazolines and their C2-selective modification in S_N_Ar reactions. In addition, we offer an approach for the synthesis of terazosin and prazosin*,* known medications against hypertension, using sulfonyl group dance products.

## Results and Discussion

### Synthesis of 4-azido-2-sulfonylquinazolines

**Scheme 2:**
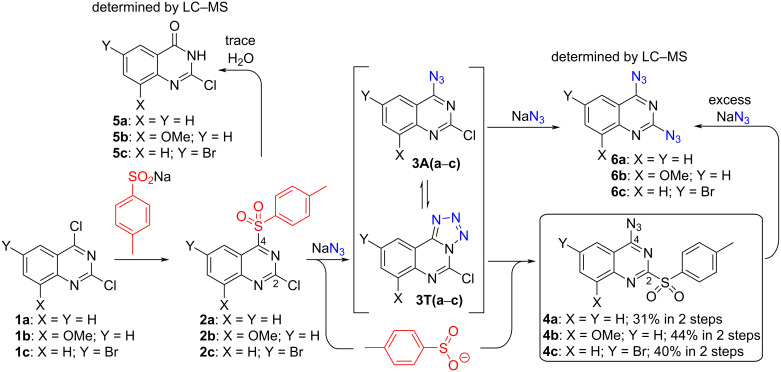
Attempts toward sulfonyl group dance using 2,4-dichloroquinazolines **1a**‒**c**.

We started our experiments with commercially available 2,4-dichloroquinazoline (**1a**). It was treated with sodium 4-methylphenylsulfinate in order to yield 4-sulfonylquinazoline **2a** (Scheme 2), but the first attempts in iPrOH did not provide the starting material conversion. The reaction in THF resulted in the full conversion of the starting material, but the analysis of the crude product revealed the quantitative formation of hydrolysis product **5a**. Assuming the instability of intermediate **2a**, a one-pot reaction was performed by adding sodium 4-methylphenylsulfinate in the first step which was followed by NaN_3_. As the result, the formation of hydrolysis product **5a** and 2,4-diazidoquinazoline (**6a**) was observed.

Next, the reaction **1a → 4a** in DMSO yielded diazidoquinazoline **6a** as a major product and hydrolysis product **5a**. In MeCN the conversion to derivative **2a** was stopped at 50% and was not facilitated by an extra addition of sulfinate. To our delight, in MeOH we observed the formation of intermediate **2a** over 5 hours, and after the subsequent addition of sodium azide product **4a** was isolated in 31% yield over 2 steps. The full conversion was achieved by keeping the reaction mixture at a temperature of 0 °C and by the stepwise additions of the sulfinate and NaN_3_. Any deviation from these conditions facilitated the formation of byproducts.

In addition, the sulfonyl group dance reactions were carried out also with quinazoline derivatives **1b** and **1c** (Scheme 2), the structure features of which may slow-down the fast S_N_Ar processes due to the substituents’ character. The desired products **4b** and **4c** were obtained in MeOH and isolated in 44 and 40% yields, respectively. Methanol is known to decrease reactivity in the S_N_Ar reactions in comparison to polar solvents such as DMSO and DMF. This is explained by solvent hydrogen bond acidity and basicity descriptors α and β, for example, α(DMSO) = 0, β(DMSO) = 0.88, α(MeOH) = 0.43, β(MeOH) = 0.47. The rate constant of the S_N_Ar process escalates with an increase of β parameters and diminishes with an increase of α parameters [21–22]. Therefore, it was possible to accomplish the sulfonyl group dance reactions of very reactive quinazolines **1a**‒**c** in MeOH.

### Synthesis of 4-azido-6,7-dimethoxy-2-sulfonylquinazolines

**Scheme 3:**
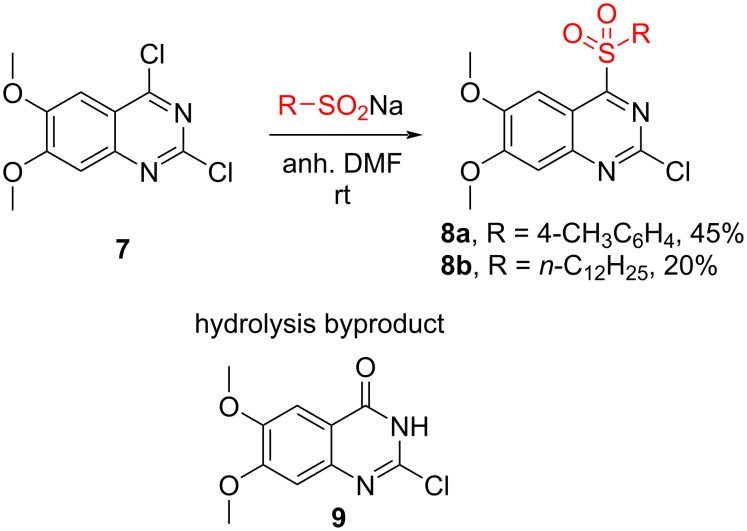
Synthesis of 2-chloro-6,7-dimethoxy-4-sulfonylquinazoline derivatives **8**.

Next, we aimed to explore the sulfonyl group dance process using a more electron-rich quinazoline. The commercially available 2,4-dichloro-6,7-dimethoxyquinazoline (**7**) was chosen for this purpose (Scheme 3). The common dimethoxy motif is also found in a variety of quinazoline-based pharmaceuticals [2–3,8,23].

We commenced our study with the preparation of 2-chloro-4-sulfonylquinazolines **8** (Scheme 3). The starting material **7** underwent S_N_Ar reactions with sodium sulfinates and the C4-substituted products **8a**,**b** were isolated [24]. The complete conversion was achieved in DMF or DMSO. In the case of sodium dodecylsulfinate, the reaction stopped at 70% conversion when 1 equivalent of sulfinate was used. Products **8** exhibited instability in the presence of water, leading to the formation of hydrolysis product **9** [25] in the reaction mixture. This instability caused issues during the reaction work-up, and attempts for purification using column chromatography resulted in full degradation of the formed product.

**Scheme 4:**
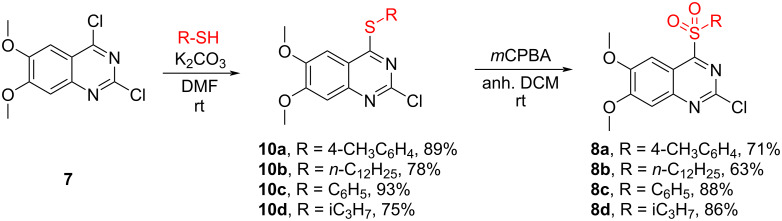
Alternative synthesis pathway for 2-chloro-6,7-dimethoxy-4-sulfonylquinazoline derivatives **8**.

Consequently, an alternative pathway toward product **8** was explored (Scheme 4). 2-Chloro-4-thioquinazolines **10** were prepared from starting material **7** in an S_N_Ar reaction with thiols in the presence of K_2_CO_3_ in good 75–93% yields. Next, thioquinazolines **10** were oxidized to the corresponding sulfonylquinazolines **8**. Inspired by our previous work [19] a TFAA/H_2_O_2_ oxidizing system was tried first but yielded several side-products, such as the hydrolysis product and unwanted oxidation of the quinazoline N3 position. Changing the oxidant to *m*CPBA (with 96% purity) [26] provided a more selective reaction, no water-based work-up was needed and the pure product was obtained by simple recrystallization from ethanol in yields up to 88%. The oxidation step thiol → sulfoxide was fast and full conversion to the intermediate was achieved in one hour for most substrates, but the step sulfoxide → sulfone was entirely slower and required stirring overnight (except for **8d** (R = iC_3_H_7_)).

**Scheme 5:**
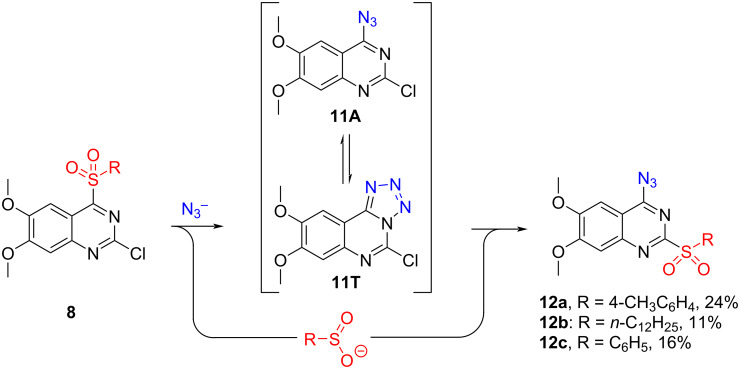
Sulfonyl group dance using 2-chloro-6,7-dimethoxy-4-sulfonylquinazolines **8**.

With 2-chloro-6,7-dimethoxy-4-sulfonylquinazolines **8** in hand, we started to explore the reactivity in S_N_Ar reactions (Scheme 5). Sulfonyl group dance reactions did not work in anhydrous THF, MeCN, and dioxane, using such azide sources as NaN_3_, LiN_3_, and TMS-N_3_. Full conversion towards product **12a** was observed by HPLC with NaN_3_ in anhydrous DMF. However, precipitation, direct, and reversed-phase column chromatography provided low yields (Scheme 5) due to the degradation of the product. Compounds **12** did not tolerate aqueous conditions or high temperatures and have also been observed to degrade under direct sunlight.

**Scheme 6:**
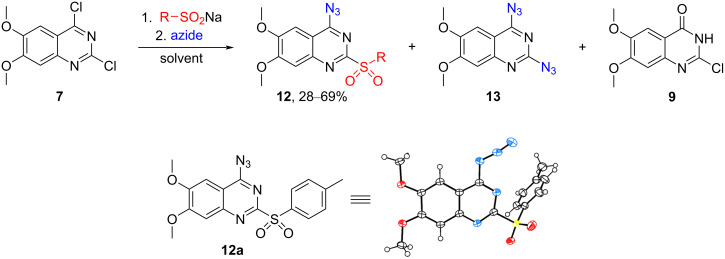
One-pot synthesis of 4-azido-6,7-dimethoxy-2-sulfonylquinazolines **12**. The crystallographic information for **12a** has been submitted to the Cambridge Crystallographic Data Centre and is available as supplementary publication No. CCDC-2312750.

Next, a stepwise one-pot approach was investigated to increase the overall yield (Scheme 6). The reaction in anhydrous DMF yielded a mixture of the desired product **12a**, diazide **13**, and hydrolysis product **9** [25] which were inseparable using common purification methods (Table 1).

**Table 1:** Conditions for one-pot synthesis of 4-azido-6,7-dimethoxy-2-sulfonylquinazolines **12**.

Entry	Solvent	Azide source	Time, h	R	Yield, %

1	anh. DMF	1.0 equiv NaN_3_^a^	1	4-CH_3_C_6_H_4_	–^b^
2	anh. DMSO	0.6 equiv NaN_3_^a^	1	4-CH_3_C_6_H_4_	**12a**, 39
3	anh. DMSO	0.8 equiv NaN_3_^a^	4	4-CH_3_C_6_H_4_	**12a**, 69
4	anh. DMSO	0.8 equiv NaN_3_^c^	2	4-CH_3_C_6_H_4_	**12a**, 66^d^
5	anh. DMSO	0.8 equiv NaN_3_^c^	12	*n*-C_12_H_25_	**12b**, 28
6	anh. DMSO	0.8 equiv NaN_3_^c^	12	iC_3_H_7_	**12d**, 63^e^
7	anh. DMSO	0.8 equiv NaN_3_^c^	2	C_6_H_5_	**12c**, 50^e^

^a^Added in portions; ^b^a mixture of products **12** and **13** (Scheme 6); ^c^0.5 M solution of NaN_3_ in anh. DMSO added over 2 hours; ^d^5.8 mmol scale; ^e^qNMR yield.

The pivotal advancement occurred when attempting the reaction in DMSO (Table 1). In the case of **8a** (R = 4-CH_3_C_6_H_4_), the product precipitated out when full conversion was reached. Filtration of this precipitate yielded the pure desired product **12a** in 39% yield (Table 1, entry 2). Incremental additions of NaN_3_, coupled with HPLC analysis following each addition, facilitated the achievement of full conversion of the starting material after 0.7–0.8 equivalents of NaN_3_. This approach limited the formation of diazide **13** and significantly elevated the yield of the desired product to 69% over two steps. When other sufinates were employed, the product failed to precipitate, necessitating isolation through preparative HPLC. Quantitative nuclear magnetic resonance (qNMR) yields were consequently reported.

To reduce the formation of diazide **13**, an overnight addition of the azide solution via a dispenser was employed at a rate of 0.1 equivalents of NaN_3_ per hour. This strategy improved the ratio of product **12** to diazide **13**. For arylsulfinates, the addition time was finally reduced to 2 hours without compromising selectivity. Although tetrabutylammonium azide (TBAA) is better soluble in DMSO than NaN_3_, practical challenges associated with its use led to the preference for NaN_3_.

### Confirmation of regioselectivity for the sulfonyl group dance products

**Scheme 7:**
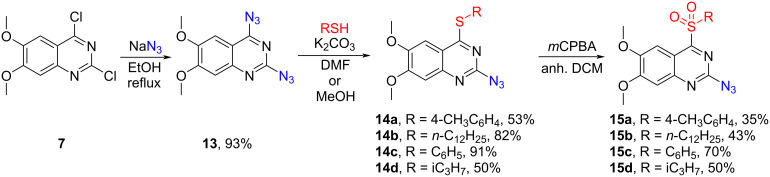
Synthesis of 2-azido-4-sulfonyl-6,7-dimethoxyquinazolines **15**.

The regioselectivity and the structure of 4-azido-6,7-dimethoxy-2-sulfonylquinazoline derivatives **12** were proven by chemical synthesis of the regioisomers **15** (Scheme 7) and X-ray analysis of **12a** (Scheme 6). 6,7-Dimethoxy-2,4-diazidoquinazoline (**13**) was synthesized from commercially available dichloroquinazoline **7** in 93% yield. Further, thioether substituents were installed in the presence of K_2_CO_3_. For alkylthiols, DMF was used, but arylthiols required milder conditions with MeOH and cooling to acquire regioselectivity to the C4 position which resulted in yields up to 91%. Oxidation with purified *m*CPBA (commercial *m*CPBA with 68% purity was washed with pH 7.4 phosphate buffer to reach 96% purity [26]) yielded the regioisomers **15** of the sulfonyl group dance products at a lower yield than the previously mentioned oxidation step, which was most likely caused by the high reactivity of product **14**, but the reaction conditions were not further optimized since the products were only needed for analytical purposes.

**Scheme 8:**
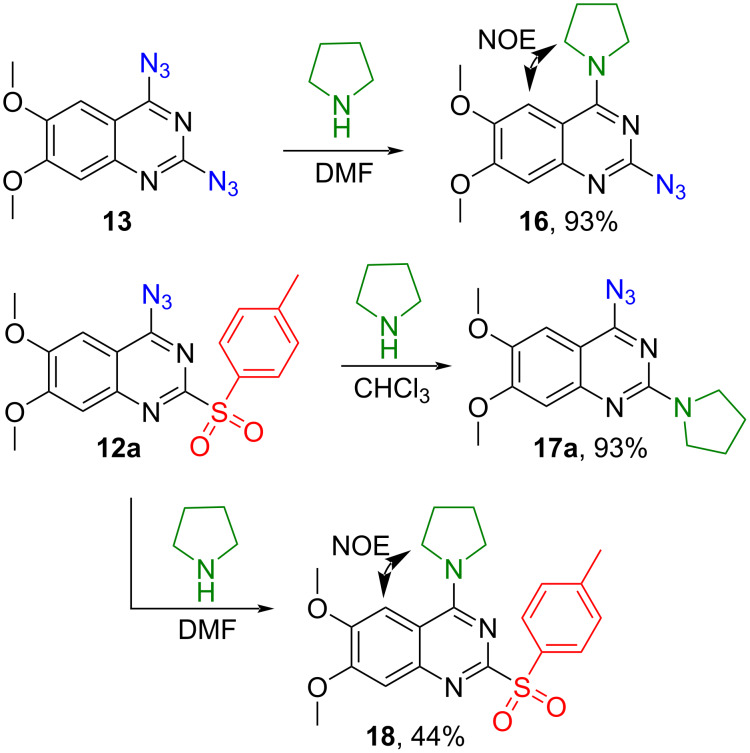
Synthesis of 6,7-dimethoxyquinazoline derivatives **16**, **17a** and **18**.

Two different pyrrolidine-substituted derivatives were additionally synthesized to prove the regioselectivity of the sulfonyl group dance products (Scheme 8). Compound **16** was obtained in the C4-selective S_N_Ar reaction between diazidoquinazoline **13** and pyrrolidine in 93% yield. A cross peak for the H–C5 position of quinazoline and CH_2_ groups of pyrrolidine at the second position was observed in the NOESY spectrum and unequivocally proved the structure **16**. Selective C2 substitution was achieved between sulfonylquinazoline **12** and pyrrolidine in CHCl_3_ yielding product **17a**. No NOESY signals were seen between the quinazoline core and the pyrrolidine moiety. Interestingly, the C4 substitution was achieved when DMF was used as a solvent in the transformation **12** → **18**, resulting in product **18**.

In addition, the reaction **12a** + pyrrolidine in MeCN and THF gave only product **17a**, but in DMSO resulted in the mixture of **17a**/**18**/6,7-dimethoxy-2,4-di(pyrrolidin-1-yl)quinazoline = 36:15:48% (HPLC analysis). The selectivity of **17a**/**18** was interesting but was not further developed in the scope of this study.

Consequently, an investigation into the azide–tetrazole equilibrium of product **12a** was initiated, revealing a singular form present in all solvents. Despite attempts to increase the amount of the azide form with the increase of the temperature in NMR experiments [27], no observable alteration in the tautomeric equilibrium was observed. FTIR analysis of **12a** in CHCl_3_ and DMSO solutions revealed the absence of the azide form (see Supporting Information File 1), precluding an explanation of the reactivity of **12a** through the tautomeric equilibrium. The presence of electron-donating methoxy groups in the structure was proposed as a plausible explanation for the present tetrazole form in the solutions. Surprisingly, FTIR and X-ray analyses of **12a** in the solid state indicated the existence of **12a** in the azide form.

In subsequent experiments it was discovered that for less nucleophilic *N*-nucleophiles (piperidine, morpholine, *N*-methylpiperazine) C2 selectivity was reached only in polar solvents such as DMF, DMSO, and MeCN. In other solvents, no reactivity was observed at the C2 or C4 positions.

### Selective modification of the C2 position of 6,7-dimethoxyquinazoline

Products **12** are useful intermediates to achieve selective modification at the C2 position of quinazolines. A scope of 2-amino-4-azido-6,7-dimetoxyquinazolines **17** was synthesized. For pyrrolidine, selective C2 substitution was achieved in a non-polar solvent such as CHCl_3_. Less nucleophilic amines gave C2-selective S_N_Ar in MeCN.

**Scheme 9:**
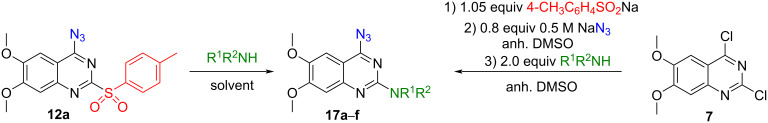
Synthesis of 2-amino-4-azido-6,7-dimethoxyquinazolines **17**.

To apply the developed technique to the synthesis of pharmaceutically active substances such as terazosin and prazosin, nucleophilic substitution at the C2 position was carried out with the corresponding amines – piperazin-1-yl(tetrahydrofuran-2-yl)methanone and furan-2-yl(piperazin-1-yl)methanone to give products **17e** and **17f**. Products **17e**,**f** can be obtained through the aromatic nucleophilic substitution of 2-azido-4-sulfonylquinazoline **12a** or by performing three subsequent S_N_Ar reactions starting from 2,4-dichloroquinazoline **7** in a one-pot procedure [28] (Scheme 9, Table 2).

**Table 2:** Diversity and yields for 2-amino-4-azido-6,7-dimethoxyquinazolines **17**.

Entry	R^1^R^2^NH	Solvent	Yield

1	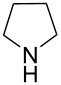	CHCl_3_	**17a**, 93^a^
2	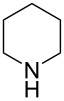	MeCN	**17b**, 73^a^
3	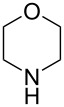	MeCN	**17c**, 75^a^
4	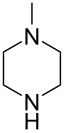	MeCN	**17d**, 77^a^
5	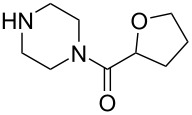	DMSO	**17e**, 80^a^, 41^b^
6	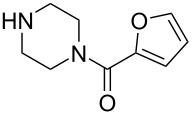	DMSO	**17f**, 75^a^, 49^b^

^a^Yield **12a→17**, %; ^b^yield **7→17**, % (over 3 steps).

The resulting products exist in an azide–tetrazole equilibrium in solution, but in solid form can be in either the azide (**17e**, **17f**) or tetrazole form (**17a**–**d**).

**Scheme 10:**
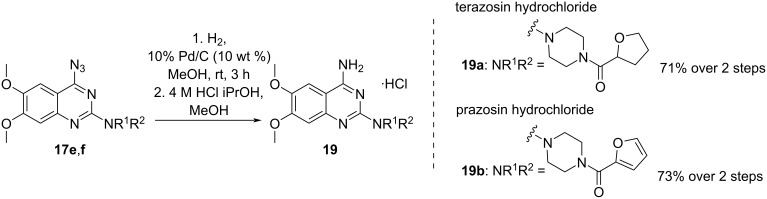
Synthesis of terazosin and prazosin hydrochlorides **19a** and **19b**.

With derivatives **17e**,**f** in hand, the reduction of the azido group in the C4 position was carried out by bubbling hydrogen through the solution in the presence of palladium on charcoal. In the last step, the product was acidified with a 4 M HCl solution in iPrOH, forming the respective hydrochlorides of terazosin [29–30] and prazosin [31–32] (Scheme 10).

**Scheme 11:**
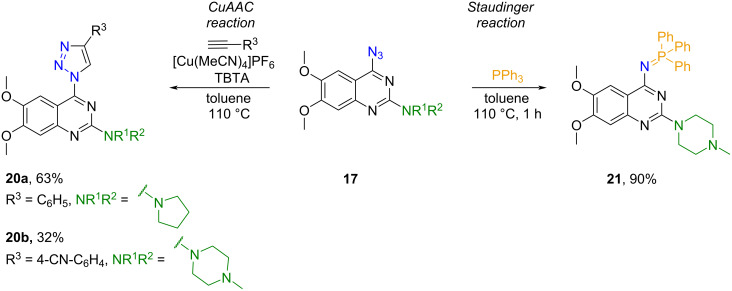
Modifications of derivatives **17**.

In addition, we explored some other reactions of the azido group, and derivatives **17** were used in CuAAC and Staudinger reactions, yielding products **20** and **21** (Scheme 11).

For CuAAC reactions no conversion towards the desired triazolyl product **20** was observed in systems such as CuSO_4_·5H_2_O/sodium ascorbate/*t*-BuOH/H_2_O, CuSO_4_·5H_2_O/sodium ascorbate/THF/H_2_O, CuI/DIPEA/DCM. Instead, triazolyl derivatives **20** were synthesized using [Cu(MeCN)_4_]PF_6_/TBTA (tris(benzyltriazolylmethyl)amine) [33] in toluene.

## Conclusion

To summarize, an approach toward 4-azido-6,7-dimethoxy-2-alkyl/arylsulfonylquinazolines was developed employing a sulfonyl group dance caused by the azide–tetrazole equilibrium in quinazolines. 4-Azido-6,7-dimethoxy-2-alkyl/arylsulfonylquinazolines were obtained using two pathways: 1) S_N_Ar reaction between 2-chloro-6,7-dimethoxy-4-sulfonylquinazoline derivatives and NaN_3_; 2) S_N_Ar reaction between 2,4-dichloro-6,7-dimethoxyquinazoline and alkyl/arylsulfinates, followed by substitution with NaN_3_. 4-Azido-6,7-dimethoxy-2-alkyl/arylsulfonylquinazolines serve as valuable precursors for the C2-regioselective modification in quinazolines. Furthermore, the developed methodology was valorized by successfully employing it in the synthesis of adrenoblockers terazosin and prazosin.

## Supporting Information

File 1 Experimental, copies of spectra and crystal data, data collection and structure refinement details for compound **12a**.

File 2 Checkcif for compound **12a**.

File 3 Crystallographic information file (CIF) for compound **12a**.

## Data Availability

All data that supports the findings of this study is available in the published article and/or the supporting information to this article.
