# Supplementary material for: Regioselective quinazoline C2 modifications through the azide–tetrazole tautomeric equilibrium
Source: Beilstein J Org Chem. 2024 Mar 28;20:675–83. doi: 10.3762/bjoc.20.61 (PMC10999978; doi:10.3762/bjoc.20.61)
Supplement: File 1 — Experimental, copies of spectra and crystal data, data collection and structure refinement details for compound 12a. [file Beilstein_J_Org_Chem-20-675-s001.pdf]

BEILSTEIN JOURNAL OF ORGANIC CHEMISTRY

Supporting Information

for

Regioselective quinazoline C2 modifications through the azide–tetrazole tautomeric equilibrium

Dāgs Dāvis Līpiņš, Andris Jeminejs, Una Ušacka, Anatoly Mishnev, Māris Turks and Irina Novosjolova

Beilstein J. Org. Chem. **2024,** *20,* 675–683. doi:10.3762/bjoc.20.61

Experimental, copies of spectra and crystal data, data collection and structure refinement details for compound 12a

License and Terms: This is a supporting information file under the terms of the Creative Commons Attribution License (https://creativecommons.org/licenses/by/4.0). Please note that the reuse, redistribution and reproduction in particular requires that the author(s) and source are credited and that individual graphics may be subject to special legal provisions.
The license is subject to the *Beilstein Journal of Organic Chemistry* terms and conditions: (https://www.beilstein-journals.org/bjoc/terms)

Experimental procedures

Copies of ^{1}H, ^{13}C, and ^{31}P NMR spectra for all compounds

FTIR spectra and X-ray data for compound 12a

Experimental

General information

Commercially available reagents were used as received. The reactions and the purity of the synthesized compounds were monitored by HPLC and TLC analysis using silica gel 60 F_{254} aluminum plates (Merck). Visualization was accomplished by UV light. Column chromatography was performed on silica gel (60 Å, 40–63 μm, ROCC). The yield of the products refers to chromatographically and spectroscopically homogeneous materials. Commercial *m*CPBA with 68% purity was washed with 7.4 pH phosphate buffer to reach 96% purity [1].

The infrared spectra were recorded in hexachlorobutadiene (4000–2000 cm^{-1}) and paraffin oil (2000–450 cm^{-1}) with a FTIR Perkin-Elmer Spectrum 100 spectrometer and in KBr tablets with a Perkin-Elmer Spectrum BX FTIR spectrometer (4000–450 cm^{-1}). Wavelengths are given in cm^{-1}.

^{1}H, ^{13}C, and ^{31}P NMR spectra were recorded with a Bruker Avance 500 spectrometer in $CDCl_3$ and DMSO-d_6. Chemical shifts (δ) are reported in ppm and coupling constants (J) in Hz. The proton ($CDCl_3$ δ = 7.26 ppm, DMSO-d_6 δ = 2.50 ppm) and carbon signals ($CDCl_3$ δ = 77.16 ppm, DMSO-d_6 δ = 39.52 ppm) for residual non-deuterated solvents were used as an internal reference for ^{1}H and ^{13}C NMR spectra, respectively. ^{1}H NMR spectra were recorded at 500 MHz and ^{13}C NMR spectra at 126 MHz. ^{31}P NMR spectra were recorded at 202 MHz with H_3PO_4 (85%) as an external standard (H_3PO_4 δ P = 0.00 ppm). The multiplicity is assigned as follows: s – singlet, d (for ^{1}H NMR) and D (for ^{13}C NMR) – doublet, t – triplet, q – quartet, m – multiplet. Nontrivial peak assignments were confirmed by $^{1}H,^{1}H$-COSY, $^{1}H,^{1}H$-HMBC, and/or $^{1}H,^{13}C$ HSQC 2D NMR experiments for representative products of each compound class.

Single-crystal diffraction data were collected on an XtaLAB Synergy-S Dualflex diffractometer (Rigaku Corporation, Tokyo, Japan) equipped with a HyPix6000 detector and micro-focus sealed X-ray tube using CuK_{α} radiation (λ = 1.54184 Å). Single crystals were fixed with oil in a nylon loop of a magnetic CryoCap and set on a goniometer head. The samples were cooled down to 150 K, and ω-scans were performed with a step size of 0.5°. Data collection and reduction were performed with the CrysAlisPro 1.171. 41.123a software (Oxford Diffraction Ltd., Abingdon, UK). Structure solution and refinement were performed with SHELXT and SHELXL software which are parts of the CrysAlisPro and Olex2 suites.

HPLC analysis was performed using an Agilent Technologies 1200 Series system equipped with an XBridge C_{18} column, 4.6 × 150 mm, particle size 3.5 µm, with a flow rate of 1 mL/min, using eluent A–0.1% TFA/H_2O with 5 vol % MeCN and eluent B–MeCN as the mobile phase. The wavelength of detection was 264 nm. Gradient: 30–95% B 5 min, 95% B 5 min, 95–30% B 2 min. LC–MS spectra were recorded with a Waters Acquity UPLC system equipped with an Acquity UPLC BEH C18 1.7 µm, 2.1 × 50 mm column, using 0.1% TFA/H_2O and MeCN as the mobile phase.

High-resolution mass spectra (ESI) were recorded with a Thermo Fisher Scientific Orbitrap Exploris 120 mass spectrometer operating in the Full Scan mode at the 120000 resolutions.

General procedures and product characterization

General procedure for the synthesis of derivatives 4a–c

4-Azido-2-tosylquinazoline (4a)

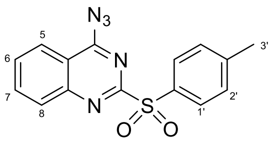

A solution of sodium 4-methylbenzenesulfinate (134 mg, 0.75 mmol, 1.5 equiv) in cold anhydrous MeOH (2 mL) was added over 1 hour to a stirred solution of 2,4-dichloroquinazoline (100 mg, 0.50 mmol, 1.0 equiv) in anhydrous MeOH (10 mL) under argon at 0 °C temperature and stirred until a full starting material conversion. After 5 hours, NaN_3 (26 mg, 0.40 mmol, 0.8 equiv) was added in portions every 5 minutes over an hour and stirred overnight. The solvent was evaporated and the solid residue was suspended in DCM (30 mL), filtered, and washed with DCM (2 × 10 mL). The filtrate was concentrated and purified by reversed-phase column chromatography ($MeCN/H_2O$ 30→100%). Yield: 51 mg, 31%. Colorless amorphous solid.

IR (KBr) ν (cm^{-1}): 3052, 2926, 2210, 2145, 1614, 1561, 1490, 1357, 1154, 1084.

^1H NMR (500 MHz, $CDCl_3$) δ (ppm): 8.17 (d, 1H, ^3J = 8.5 Hz, H-C(8)), 8.07 (m, 3H, H-C(5), 2 x H-C(1')), 7.99 (t, 1H, ^3J = 7.8 Hz, H-C(6)), 7.70 (t, 1H, ^3J = 7.6 Hz, H-C(7)), 7.37 (d, 2H, ^3J = 8.1 Hz, 2 x H-C(2')), 2.45 (s, 3H, (H_3C-)).

^{13}C NMR (126 MHz, $CDCl_3$) δ (ppm): 164.3, 161.4, 150.6, 145.6, 136.1, 134.7, 130.1 (2C), 129.8, 129.5, 123.9, 117.3, 21.9.

HRMS (ESI) m/z: $[M+H]^+$ calcd. for $C_{15}H_{12}N_5O_2S$, 326.0706; found, 326.0703.

4-Azido-8-methoxy-2-tosylquinazoline (4b)

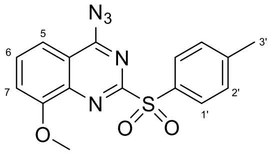

Reaction conditions: 4-methylbenzenesulfinate (117 mg, 0.65 mmol, 1.5 equiv), 2,4-dichloro-8-methoxyquinazoline (100 mg, 0.44 mmol, 1.0 equiv), anhydrous MeOH (10 mL), NaN_3 (23 mg, 0.35 mmol, 0.8 equiv). Purified by reversed-phase column chromatography ($MeCN/H_2O$ 30→100%). Yield: 68 mg, 44%. Yellow amorphous solid.

IR (KBr) ν (cm^{-1}): 2925, 2854, 2150, 1605, 1487, 1391, 1356, 1329 1143, 1085.

^1H NMR (500 MHz, $CDCl_3$) δ (ppm): 8.11 (d, 2H, ^3J = 8.1 Hz, 2 x H-C(2')), 7.65 – 7.54 (m, 2H, H-C(5), H-C(6)), 7.36 (d, 2H, ^3J = 8.1 Hz, 2 x H-C(1')), 7.31 (d, 1H, ^3J = 7.5 Hz, H-C(7)), 4.05 (s, 3H, H_3C-O), 2.44 (s, 3H, (H_3C-)).

^{13}C NMR (126 MHz, $CDCl_3$) δ (ppm): 163.9, 160.3, 155.7, 145.4, 142.3, 134.9, 130.6, 130.2, 129.7, 118.3, 114.8, 114.4, 56.6, 21.9.

HRMS (ESI) m/z: $[M+H]^+$ calcd. for $C_{16}H_{14}N_5O_3S$, 356.0812, found, 356.0810.

4-Azido-6-bromo-2-tosylquinazoline (4c)

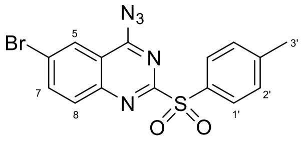

Reaction conditions: 4-methylbenzenesulfinate (96 mg, 0.54 mmol, 1.5 equiv), 6-bromo-2,4-dichloroquinazoline (100 mg, 0.36 mmol, 1.0 equiv), anhydrous MeOH (10 mL), NaN_3 (19 mg, 0.29 mmol, 0.8 equiv). Purified by reverse-phase column chromatography ($MeCN/H_2O$ 30→70%). Yield: 59 mg, 40%. Yellow amorphous solid.

IR (KBr) ν (cm^{-1}): 3047, 2922, 2851, 2143, 1600, 1555, 1478, 1366, 1335, 1147.

^1H NMR (500 MHz, $CDCl_3$) δ (ppm): 8.22 (d, 1H, ^3J = 2.0 Hz, H-C(5)), 8.12 – 7.96 (m, 4H, 2 x H-C(2'), H-C(7), H-C(8)), 7.38 (d, 2H, ^3J = 8.2 Hz, 2 x H-C(1')), 2.45 (s, 3H, (H_3C-)).

^{13}C NMR (126 MHz, $CDCl_3$) δ (ppm): 163.3, 161.6, 149.3, 145.8, 139.7, 134.5, 131.0, 130.1, 129.9, 126.4, 124.3, 118.2, 21.9.

HRMS (ESI) m/z: $[M+H]^+$ calcd. for $C_{15}H_{11}BrN_5O_2S$, 403.9811; found, 403.9811.

General procedure for the synthesis of 2-chloro-6,7-dimethoxy-4-sulfonylquinazolines 8:

2-Chloro-6,7-dimethoxy-4-tosylquinazoline (8a) [2]

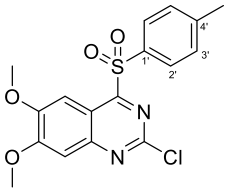

Method A: Sodium *p*-tolylsulfinate (100 mg, 0.55 mmol, 1.1 equiv) was added to a stirred mixture of 2,4-dichloro-6,7-dimethoxyquinazoline (130 mg, 0.50 mmol, 1.0 equiv) in anhydrous DMF (4 mL) under argon. Upon full starting material conversion, the reaction mixture was diluted with toluene (30 mL), washed with 11% NaCl solution (3 × 4 mL), dried over anhydrous Na_2SO_4, evaporated and recrystallized from EtOH (5 mL). Precipitate was filtered, washed with cold EtOH (2 × 3 mL), and dried in vacuum. Yield: 86 mg, 45%. Neon green crystalline solid.

Method B: Anhydrous DCM (5 mL) was added to a mixture of 2-chloro-6,7-dimethoxy-4-(-*p*-tolylthio)quinazoline **10a** (0.31 mmol, 1.0 equiv) and *m*CPBA (158 mg, 0.92 mmol, 3.0 equiv). The reaction mixture was stirred at rt under argon for 2.5 hours, controlled by HPLC. Upon completion the solvent was evaporated and the mixture was recrystallized from EtOH (2.5 mL). Precipitate was filtered, washed with cold EtOH (2 × 3 mL), and dried in vacuum. Yield: 82 mg, 71%. Neon green crystalline solid.

2-Chloro-4-(dodecylsulfonyl)-6,7-dimethoxyquinazoline (8b)

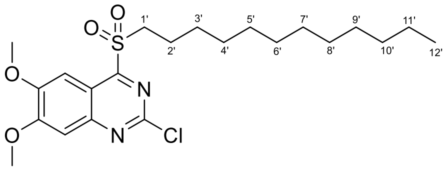

Method A: Sodium dodecylsulfinate (141 mg, 0.55 mmol, 1.1 equiv), 2,4-dichloro-6,7-dimethoxyquinazoline (100 mg, 0.50 mmol, 1.0 equiv). Yield: 47 mg, 20%. Colorless crystalline solid.

Method B: 2-Chloro-4-(dodecylthio)-6,7-dimethoxyquinazoline **10b** (100 mg, 0.24 mmol, 1.0 equiv), *m*CPBA (102 mg, 0.59 mmol, 2.5 equiv) Yield: 67 mg, 63%. Colorless crystalline solid.

IR (KBr) ν (cm^{-1}): 2915, 2848, 1611, 1507, 1418, 1408, 1297, 1245, 1139, 1011.

^{1}H NMR (500 MHz, $CDCl_3$) δ (ppm): 8.10 (s, 1H, H-C(Ar)), 7.34 (s, 1H, H-C(Ar)), 4.08, 4.07 (2s, 6H, 2x (-OCH_3)), 3.70 (dd, 2H, ^3J = 9.2 Hz, ^3J = 6.6 Hz, H_2-C(1')), 1.95 (quintet, 2H, ^3J = 7.7 Hz, H_2-C(2')), 1.53 (quintet, 2H, ^3J = 7.4 Hz, H_2-C(3')), 1.41–1.21 (m, 16H, 8 x H_2-C), 0.88 (t, ^3J = 7.0 Hz, 3H, H_3-C(12')).

^{13}C NMR (126 MHz, $CDCl_3$) δ (ppm): 162.7, 158.4, 153.6, 153.4, 152.1, 114.4, 106.5, 101.8, 56.9, 56.8, 51.6, 32.0, 29.7 (2C), 29.6, 29.5, 29.4, 29.1, 28.5, 22.8, 22.1, 14.3.

HRMS (ESI) m/z: $[M + H]^+$ calcd for $C_{22}H_{34}ClN_2O_4S$, 457.1922; found, 457.1903.

2-Chloro-6,7-dimethoxy-4-(phenylsulfonyl)quinazoline (8c)

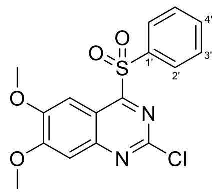

Method B: 2-Chloro-4-(phenylthio)-6,7-dimethoxyquinazoline **10c** (105 mg, 0.32 mmol, 1.0 equiv), *m*CPBA (163 mg, 0.95 mmol, 3.0 equiv). Yield: 101 mg, 88%. Colorless crystalline solid.

IR (KBr) ν (cm^{-1}): 1707, 1500, 1413, 1324, 1240, 1146, 1082, 1011.

^{1}H NMR (500 MHz, $CDCl_3$) δ (ppm): 8.33 (s, 1H, H-C(Ar)), 8.10 (d, 2H, ^3J = 7.5 Hz, H-C(2')), 7.73 (t, 1H, ^3J = 7.5 Hz, H-C(4')), 7.62 (t, 2H, ^3J = 7.6 Hz, H-C(3')), 7.32 (s, 1H, H-C(Ar)), 4.13, 4.07 (2s, 6H, 2 x (-OCH_3)).

^{13}C NMR (126 MHz, $CDCl_3$) δ (ppm): 163.7, 158.3, 153.7 (2C), 152.1, 137.2, 134.8, 129.9, 129.3, 114.8, 106.5, 102.1, 56.9, 56.8.

HRMS (ESI) m/z: $[M + H]^+$ calcd for $C_{16}H_{14}ClN_2O_4S$, 365.0357; found, 365.0342.

2-Chloro-4-(isopropylsulfonyl)-6,7-dimethoxyquinazoline (8d)

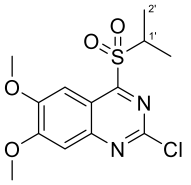

Method B: 2-Chloro-4-(isopropylthio)-6,7-dimethoxyquinazoline **10d** (50 mg, 0.17 mmol, 1 equiv), *m*CPBA (72 mg, 0.42 mmol, 2.5 equiv). Yield: 47 mg, 86%. Colorless crystalline solid.

IR (UATR) ν (cm^{-1}): 3014, 2975, 2875, 1610, 1500, 1300, 1238, 1137, 1007.

^{1}H NMR (500 MHz, $CDCl_3$) δ (ppm): 8.22 (s, 1H, H-C(Ar)), 7.33 (s, 1H, H-C(Ar)), 4.32 (septet, 1H, ^3J = 6.9 Hz, H-C(1')), 4.08, 4.07 (2s, 6H, 2 x (-OCH_3)), 1.50 (d, 6H, ^3J = 6.9 Hz, 2 x H_3-C(2')).

^{13}C NMR (126 MHz, $CDCl_3$) δ (ppm) 161.9, 158.4, 153.7, 153.5, 152.1, 115.48, 106.5, 101.9, 56.9, 56.7, 51.6, 15.2.

HRMS (ESI) m/z: $[M + H]^+$ calcd for $C_{13}H_{16}ClN_2O_4S$, 331.0514; found, 331.0504.

General procedure for the synthesis of 2-chloro-6,7-dimethoxy-4-thioquinazolines 10:

2-Chloro-6,7-dimethoxy-4-(*p*-tolylthio)quinazoline (10a)

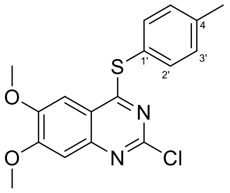

4-Methylbenzenethiol (68 mg, 0.55 mmol, 1.1 equiv) was added to a stirred solution of 2,4-dichloro-6,7-dimethoxyquinazoline (130 mg, 0.5 mmol, 1.0 equiv) and K_2CO_3 (83 mg, 0.6 mmol, 1.2 equiv) in DMF (5 mL) at room temperature. After HPLC monitoring showed full conversion of the starting material, H_2O was added (20 mL) to the reaction mixture and left to cool in the freezer. The suspension was filtered and washed with H_2O (2 × 3 mL) and cold EtOH (3 × 3 mL). The precipitate was collected and dried in vacuum. Yield: 155 mg; 89%. Colorless amorphous solid.

IR (KBr) ν (cm^{-1}): 3010, 2958, 2910, 1508, 1412, 1346, 1234, 1140.

^1H NMR (500 MHz, DMSO-d_6) δ (ppm): 7.54 (d, 2H, ^3J = 8.0 Hz, 2 x H-C(3')), 7.36 (d, 2H, ^3J = 8.0 Hz, 2 x H-C(2')), 7.32 (s, 1H, H-C(Ar)), 7.31 (s, 1H, H-C(Ar)), 3.98 (s, 6H, 2 x (-OCH_3)), 2.40 (s, 3H, -CH_3).

^{13}C NMR (126 MHz, DMSO-d_6) δ (ppm): 169.7, 156.7, 153.5, 150.4, 147.4, 139.9, 135.4, 130.3, 122.7, 116.0, 106.4, 101.1, 56.5, 56.1, 20.9.

HRMS (ESI) m/z: $[M + H]^+$ calcd for $C_{17}H_{16}ClN_2O_2S$, 347.0616; found, 347.0603.

2-Chloro-4-(dodecylthio)-6,7-dimethoxyquinazoline (10b)

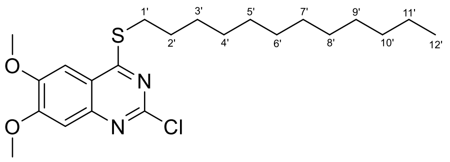

Dodecane-1-thiol (122 mg, 0.6 mmol, 0.15 mL, 1.2 equiv) 2,4-dichloro-6,7-dimethoxyquinazoline (130 mg, 0.5 mmol, 1.0 equiv), K_2CO_3 (90 mg, 0.65 mmol, 1.3 equiv). Yield: 166 mg, 78%. Colorless amorphous solid.

IR (KBr) ν (cm^{-1}): 2917, 2850, 1505, 1412, 1343, 1234, 1139, 1016, 988.

^1H NMR (500 MHz, $CDCl_3$) δ (ppm): 7.183 (s, 1H, H-C(Ar)), 7.178 (s, 1H, H-C(Ar)), 4.02, 4.01 (2s, 6H, 2 x (-OCH_3)), 3.38 (t, 2H, ^3J = 7.3 Hz, H_2-C(1')), 1.79 (quintet, 2H,

^3J = 7.4 Hz, H_2-C(2')), 1.49 (quintet, 2H, ^3J = 7.4 Hz, H_2-C(3')), 1.40–1.21 (m, 16H, 8×(-CH_2-)), 0.87 (t, 3H, ^3J = 7.0 Hz, H_3-C(12')).

^{13}C NMR (126 MHz, $CDCl_3$) δ (ppm): 171.1, 156.4, 154.9, 150.1, 147.4, 117.5, 106.7, 101.8, 56.6, 56.5, 32.1, 30.3, 29.8, 29.8, 29.7, 29.6, 29.5, 29.3, 29.0, 28.9, 22.8, 14.3.

HRMS (ESI) m/z: $[M + H]^+$ calcd for $C_{22}H_{34}ClN_2O_2S$, 425.2024; found, 425.2011.

2-Chloro-6,7-dimethoxy-4-(phenylthio)quinazoline (10c)

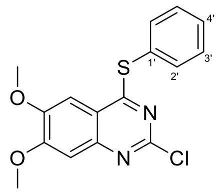

Benzenethiol (309 mg, 2.8 mmol, 0.29 mL, 1.1 equiv), 2,4-dichloro-6,7-dimethoxyquinazoline (660 mg, 2.6 mmol, 1.0 equiv), K_2CO_3 (431 mg, 3.12 mmol, 1.2 equiv). Yield: 766 mg; 93%. Colorless amorphous solid.

IR (KBr) ν (cm^{-1}): 2920, 2853, 1611, 1504, 1414, 1341, 1235, 1125, 987.

^{1}H NMR (500 MHz, $CDCl_3$) δ (ppm): 7.65–7.60 (m, 2H, 2 x H-C(3')), 7.50–7.46 (m, 3H, 2 x H-C(2'), 1 x H-C(4')), 7.28 (s, 1H, H-C(Ar)), 7.19 (s, 1H, H-C(Ar)), 4.04, 4.02 (2s, 6H, 2 x (-OCH_3)).

^{13}C NMR (126 MHz, $CDCl_3$) δ (ppm): 170.1, 156.6, 155.0, 150.4, 148.2, 135.5, 129.9, 129.5, 127.0, 116.9, 106.7, 101.6, 56.6, 56.5.

HRMS (ESI) m/z: $[M + H]^+$ calcd for $C_{16}H_{14}ClN_2O_2S$, 333.0459; found, 333.0446.

2-Chloro-4-(isopropylthio)-6,7-dimethoxyquinazoline (10d)

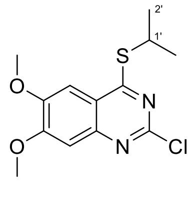

Propane-2-thiol (42 mg, 0.55 mmol, 0.05 mL, 1.1 equiv) 2,4-dichloro-6,7-dimethoxyquinazoline (130 mg, 0.5 mmol, 1.0 equiv), K_2CO_3 (83 mg, 0.6 mmol, 1.2 equiv). Yield: 112 mg; 75%. Colorless amorphous solid.

IR (KBr) ν (cm^{-1}): 2961, 1615, 1569, 1501, 1413, 1241, 1144, 987.

^{1}H NMR (500 MHz, $CDCl_3$) δ (ppm): 7.17 (s, 1H, H-C(Ar)), 7.14 (s, 1H, H-C(Ar)), 4.31 (septet, 1H, ^3J = 6.9 Hz, H-C(*i*-Pr)), 4.00 (s, 6H, 2 x (-OCH_3)), 1.51 (d, 6H, ^3J = 6.9 Hz, 2 x H_3-C).

^{13}C NMR (126 MHz, $CDCl_3$) δ (ppm): 171.0, 156.4, 154.8, 150.1, 147.5, 117.4, 106.7, 101.8, 56.6, 56.4, 36.0, 23.0.

HRMS (ESI) m/z: $[M + H]^+$ calcd for $C_{13}H_{16}ClN_2O_2S$, 299.0616; found, 299.0604.

General procedure for the synthesis of 4-azido-6,7-dimethoxy-2-sulfonylquinazolines 12:

4-Azido-6,7-dimethoxy-2-tosylquinazoline (12a)

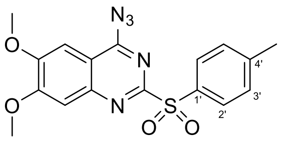

<u>Method C</u>: A mixture of 2,4-dichloro-6,7-dimethoxyquinazoline (1.5 g, 5.8 mmol, 1.0 equiv) and the corresponding sodium sulfinate (6.1 mmol, 1.05 equiv) was co-evaporated thrice from toluene (3 mL) and dried in vacuum. Anhydrous DMSO (8 mL) was added and the reaction mixture was stirred under argon at rt, controlled by HPLC. After 30 minutes a 0.5 M solution of sodium azide in anhydrous DMSO (9.25 mL, 4.63 mmol, 0.8 equiv) was added over 2 hours. The reaction mixture was filtered, the precipitate was washed with H_2O (2 × 8 mL) and cold EtOH (2 × 8 mL), collected and dried in vacuum. Yield: 1.47 g, 66%. Colorless amorphous solid.

Method D: Sodium azide (11 mg, 0.17 mmol, 0.9 equiv) in anhydrous DMSO (1 mL) was added to a suspension of 2-chloro-6,7-dimethoxy-4-sulfonylquinazoline (70 mg, 0.19 mmol, 1.0 equiv) in anhydrous DMSO (5 mL) at a rate of 0.1 mL/h. The reaction mixture was lyophilized, the residue was dissolved in DCM (10 mL) and washed with H_2O (2 × 5 mL). Aqueous layer was back-extracted with DCM (2 × 3 mL), and the combined organic layer was washed with Brine (2 x 2 mL) and dried with anhydrous Na_2SO_4. The solvent was evaporated and the solid residue recrystallized from MeCN (5 mL). Yield: 17 mg, 24%. Colorless amorphous solid.

Method E: Sodium sulfinate (358 mg, 2.1 mmol, 1.05 equiv) was added to a suspension of 2,4-dichloro-6,7-dimethoxyquinazoline (500 mg, 2.0 mmol, 1.0 equiv) in anhydrous DMSO (5 mL) under argon. Upon full starting material conversion NaN_3 (104 mg, 1.6 mmol. 0.8 equiv) was added in portions of ≈5 mg, with HPLC analysis after each addition until full conversion. The reaction mixture was filtered, the precipitate was washed with H_2O (2 × 4 mL) and cold EtOH (2 × 4 mL), collected, and freeze-dried. Yield: 525 mg, 69%. Colorless amorphous solid.

Method for crystal growth of compound **12a**: slow evaporation. Solvent system: toluene/$CHCl_3$ 2:1.

IR (KBr) ν (cm^{-1}): 3076, 2130, 1608, 1500, 1423, 1316, 1253, 1220, 1080.

^1H NMR (500 MHz, $CDCl_3$) δ (ppm): 8.07 (d, 2H, ^3J = 7.8 Hz, 2×H-C(Ar)), 7.49 (s, 1H, H-C(Ar)), 7.36 (d, 2H, ^3J = 7.8 Hz, 2×H-C(Ar)), 7.19 (s, 1H, H-C(Ar)), 4.03, 4.01 (2s, 6H, 2 x (-OCH_3)), 2.44 (s, 3H, -CH_3).

^{13}C NMR (126 MHz, $CDCl_3$) δ (ppm): 160.7, 160.1, 157.5, 152.2, 148.5, 145.3, 135.2, 129.9, 129.8, 112.5, 108.1, 101.1, 56.9, 56.6, 21.9.

HRMS (ESI) m/z: $[M + H]^+$ calcd for $C_{17}H_{16}N_5O_4S$, 386.0918; found, 386.0902.

4-Azido-2-(dodecylsulfonyl)-6,7-dimethoxyquinazoline (12b)

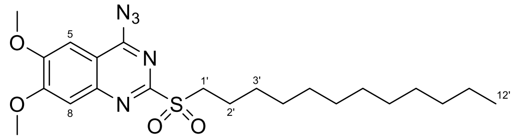

Method C: 2,4-Dichloro-6,7-dimethoxyquinazoline (104 mg, 0.4 mmol, 1.0 equiv), sodium dodecylsulfinate (106 mg, 0.42 mol, 1.05 equiv), a 0.5 M solution of NaN_3 in anhydrous DMSO (0.6 mL, 0.32 mmol, 0.8 equiv). Purified by silica gel column chromatography (DCM/MeCN 1→5%). Yield: 51 mg, 28%. Colorless amorphous solid.

Method D: NaN_3 (9 mg, 0.14 mmol, 1 equiv), 2-chloro-4-(dodecylsulfonyl)-6,7-dimethoxyquinazoline (63 mg, 0.14 mmol, 1.0 equiv). Purified by silica gel column chromatography (DCM/MeCN 1→5%). Yield: 7 mg, 11%. Colorless amorphous solid.

R_f = 0.8 (DCM/MeCN 10:1).

IR (KBr) ν (cm^{-1}): 2921, 2852, 2125, 1610, 1508, 1426, 1313, 1238, 1125, 989.

^1H NMR (500 MHz, $CDCl_3$) δ (ppm): 7.48 (s, 1H, H-C(Ar)), 7.28 (s, 1H, H-C(Ar)), 4.06, 4.05 (2s, 6H, 2 x (-OCH_3)), 3.60–3.50 (m, 2H, H_2-C(1')), 1.95–1.84 (m, 2H, H_2-C(2')), 1.50-1.41 (m, 2H, H_2-C(3')), 1.36–1.19 (m, 16H, 8 x H_2-C(4'-11')), 0.87 (t, ^3J = , 3H, H_3-C(12')).

^{13}C NMR (126 MHz, $CDCl_3$) δ (ppm): 160.7, 159.2, 157.8, 152.3, 148.5, 112.9, 107.8, 101.2, 56.9, 56.7, 51.6, 32.0, 29.7 (2C), 29.6, 29.5, 29.4, 29.1, 28.6, 22.8, 22.3, 14.3.

HRMS (ESI) m/z: $[M + H]^+$ calcd for $C_{22}H_{34}N_5O_4S$, 464.2326; found, 464.2307

4-Azido-6,7-dimethoxy-2-(phenylsulfonyl)quinazoline (12c)

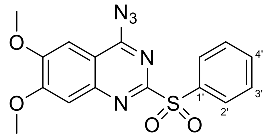

Method C: 2,4-Dichloro-6,7-dimethoxyquinazoline (100 mg, 0.39 mmol, 1.0 equiv), sodium phenylsulfinate (67 mg, 0.41 mol, 1.05 equiv), a 0.5 M solution of NaN_3 in

anhydrous DMSO (0.6 mL, 0.31 mmol, 0.8 equiv). qNMR yield with 1,2,3-trimethoxybenzene as an internal standard: 50%.

Method D: NaN_3 (15 mg, 0.23 mmol, 1.0 equiv), 2-chloro-6,7-dimethoxy-4-phenylsulfonylquinazoline (83 mg, 0.23 mmol, 1.0 equiv). Yield: 48 mg with purity of 28%, qNMR yield: 16%.

For analytical purposes, the compound was purified by preparative HPLC.

IR (KBr) ν (cm^{-1}): 2956, 2921, 2850, 2136, 1501, 1423, 1251, 1225, 1140, 1082, 856.

^{1}H NMR (500 MHz, $CDCl_3$) δ (ppm): 8.20 (d, 2H, ^3J = 7.7 Hz, H-C(2')), 7.66 (t, 1H, ^3J = 7.5 Hz, H-C(4')) , 7.57 (dd, 2H, ^3J = 7.7 Hz, ^3J = 7.5 Hz, H-C(3')), 7.48 (s, 1H, H-C(Ar)), 7.20 (s, 1H, H-C(Ar)), 4.04, 4.01 (2s, 6H, 2 x (-OCH_3)).

^{13}C NMR (126 MHz, $CDCl_3$) δ (ppm): 160.7, 159.9, 157.6, 152.3, 148.5, 138.2, 134.2, 129.9, 129.1, 112.6, 108.0, 101.1, 56.9, 56.6.

HRMS (ESI) m/z: $[M + H]^+$ calcd for $C_{16}H_{14}N_5O_4S$, 372.0761; found, 372.0745.

4-Azido-2-(isopropylsulfonyl)-6,7-dimethoxyquinazoline (12d)

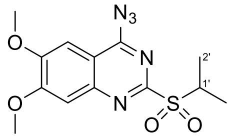

Method C: 2,4-Dichloro-6,7-dimethoxyquinazoline (100 mg, 0.39 mmol, 1.0 equiv), sodium isopropylsulfinate (200 mg, 1.5 mol, 4.0 equiv), a 0.5 M solution of sodium azide in anhydrous DMSO (0.62 mL, 0.31 mmol, 0.8 equiv). qNMR yield with 1,2,3-trimethoxybenzene as an internal standard: 63%.

Method D: NaN_3 (14 mg, 0.21 mmol, 1.0 equiv), 2-chloro-4-(isopropylsulfonyl)-6,7-dimethoxyquinazoline (70 mg, 0.21 mmol, 1.0 equiv). Yield: 50 mg with purity 47%; qNMR yield with 1,2,3-trimethoxybenzene as an internal standard: 34%.

For analytical purposes, the compound was purified by preparative HPLC.

IR (KBr) ν (cm^{-1}): 2920, 2852, 2128, 1609, 1508, 1365, 1311, 1121, 993.

^{1}H NMR (500 MHz, $CDCl_3$) δ (ppm): 7.50 (s, 1H, H-C(Ar)), 7.28 (s, 1H, H-C(Ar)), 4.05 (2s, 6H, 2 x (-OCH_3)), 4.01 (septet, 1H, ^{3}J = 7.0 Hz, H-C(1')), 1.45 (d, 6H, ^{3}J = 6.9 Hz, 2 x H_3-C(2')).

^{13}C NMR (126 MHz, $CDCl_3$) δ (ppm): 160.6, 158.4, 157.7, 152.3, 148.6, 112.9, 107.9, 101.2, 56.9, 56.7, 51.6, 15.2.

HRMS (ESI) m/z: $[M + H]^+$ calcd for $C_{13}H_{16}N_5O_4S$, 338.0918; found, 338.0905.

Synthesis of 2,4-diazido-6,7-dimethoxyquinazoline 13

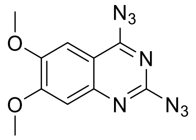

A suspension of 2,4-dichloro-6,7-dimethoxyquinazoline (500 mg, 1.93 mmol, 1.0 equiv) and NaN_3 (375 mg, 5.76 mmol, 3.0 equiv) was refluxed in EtOH (5 mL) for 45 minutes. The reaction mixture was cooled in the freezer for 5 minutes, filtered, and washed with H_2O (2 × 3 mL) and cold EtOH (2 × 3 mL). The solid residue was collected and dried in vacuum. Yield: 489 mg, 93%. Colorless amorphous solid.

IR (KBr) ν (cm^{-1}): 3028, 2941, 2844, 2134, 1615, 1542, 1528, 1383, 1248, 1216, 1018.

^{1}H NMR (500 MHz, $CDCl_3$) δ (ppm): 7.90 (s, 1H, H-C(Ar)), 7.47 (s, 1H, H-C(Ar)), 4.17, 4.05 (2s, 6H, 2 x (-OCH_3)).

^{13}C NMR (126 MHz, $CDCl_3$) δ (ppm): 159.7, 157.4, 152.4, 150.2, 130.1, 106.9, 105.2, 97.7, 57.4, 56.7.

HRMS (ESI) m/z: $[M + H]^+$ calcd for $C_{10}H_9N_8O_2$, 273.0843; found, 273.0837.

General procedure for the synthesis of 2-azido-6,7-dimethoxy-4-thioquinazolines 14:

2-Azido-6,7-dimethoxy-4-(*p*-tolylthio)quinazoline (14a)

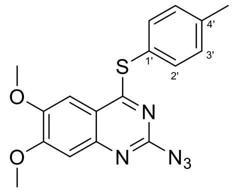

Method F*:* A solution of the 4-methylbenzenethiol (73 mg, 0.59 mmol, 1.5 equiv) in MeOH (2 mL) was added over one hour to a stirred solution of 2,4-diazido-6,7-dimethoxyquinazoline (100 mg, 0.39 mmol, 1.0 equiv) and K_2CO_3 (83 mg, 0.59 mmol, 1.5 equiv) in MeOH (5 mL) under argon at −5 °C. The reaction mixture was stirred overnight at rt, cooled in the freezer and filtered. The precipitate was washed with H_2O (2 × 3 mL) and cold EtOH (2 × 3 mL), collected, and dried in vacuum. Yield: 70 mg, 53%. Colorless amorphous solid.

IR (UATR) ν (cm^{-1}): 2959, 2924, 1959, 1613, 1595, 1497, 1373, 1249, 1200, 1041.

^1H NMR (500 MHz, $CDCl_3$) δ (ppm): 7.88 (s, 1H, H-C(Ar)), 7.59 (s, 1H, H-C(Ar)), 7.51 (d, 2H, ^3J = 7.8 Hz, H-C(3)), 7.30 (s, 2H, ^3J = 7.8 Hz, H-C(2)), 4.16, 4.11 (2s, 6H, 2 x (-OCH_3)), 2.44 (s, 3H, H_3C(5’)).

^{13}C NMR (126 MHz, $CDCl_3$) δ (ppm): 169.6, 156.4, 152.4, 150.0, 141.0, 136.0, 130.7, 127.9, 122.0, 111.9, 105.1, 97.7, 57.3, 56.7, 21.6.

HRMS (ESI) m/z: $[M + H]^+$ calcd for $C_{17}H_{16}N_5O_2S$, 354.1019; found, 354.1006.

2-Azido-4-(dodecylthio)-6,7-dimethoxyquinazoline (14b)

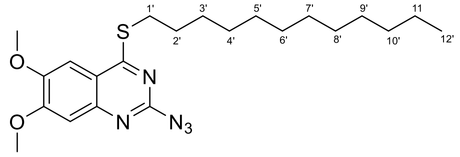

Method G: To a suspension of 2,4-diazido-6,7-dimethoxyquinazoline (50 mg, 0.18 mmol, 1.0 equiv) and K_2CO_3 (35 mg, 0.25 mmol, 1.35 equiv) in DMF (5 mL), corresponding dodecylthiol (52 µL, 0.22 mmol, 1.2 equiv) was added and stirred at rt, controlled by HPLC. Upon completion H_2O (15 mL) was added to the reaction mixture, cooled in the freezer and filtered. Precipitate was washed with H_2O (2 × 3 mL) and dissolved in DCM. The solvent was evaporated and the solid residue was dried in vacuum. Yield: 65 mg, 82% colorless amorphous solid.

IR (UATR) ν (cm^{-1}): 2915, 2847, 1617, 1598, 1536, 1376, 1252, 1204, 1044, 931.

^{1}H NMR (500 MHz, $CDCl_3$) δ (ppm): 7.86 (s, 1H, H-C(Ar)), 7.47 (s, 1H, H-C(Ar)), 4.15, 4.06 (2s, 6H, 2 x (-OCH_3)), 3.49 (t, 2H, ^3J = 7.3 Hz, H_2-C(1’)), 1.88–1.78 (m, 2H, H_2-C(2’)), 1.55–1.46 (m, 2H, H_2-C(3’)), 1.39–1.19 (m, 16H, 8×(-CH_2-)), 0.87 (t, 3H, ^3J = 6.9 Hz, H_3-C(12’)).

^{13}C NMR (126 MHz, $CDCl_3$) δ (ppm): 169.8, 156.2, 152.5, 149.9, 127.6, 112.5, 105.3, 97.6, 57.3, 56.7, 32.0, 30.9, 29.8, 29.8, 29.7, 29.7, 29.5, 29.3, 29.1, 28.8, 22.8, 14.3.

HRMS (ESI) m/z: $[M + H]^+$ calcd for $C_{22}H_{34}N_5O_2S$, 432.2428; found, 432.2409.

2-Azido-6,7-dimethoxy-4-(phenylthio)quinazoline (14c)

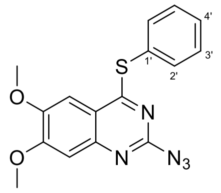

Method F: benzenethiol (62 µL, 67 mg, 0.6 mmol, 1.5 equiv), 2,4-diazido-6,7-dimethoxyquinazoline (110 mg, 0.4 mmol, 1.0 equiv), K_2CO_3 (84 mg, 0.61 mmol, 1.5 equiv). Yield: 124 mg, 91%. Colorless amorphous solid.

IR (UATR) ν (cm^{-1}): 3089, 2982, 2928, 1611, 1593, 1533, 1371, 1309, 1251, 1043.

^{1}H NMR (500 MHz, $CDCl_3$) δ (ppm): 7.91 (s, 1H, H-C(Ar)), 7.65 (d, 2H, ^3J = 6.7 Hz, H-C(2')), 7.61 (s, 1H, H-C(Ar)), 7.56–7.50 (m, 3H, 2 x H-C(3'), 1 x H-C(4')), 4.17, 4.12 (2s, 6H, 2 x (-OCH_3)).

^{13}C NMR (126 MHz, $CDCl_3$) δ (ppm): 169.2, 156.5, 150.1, 136.2, 136.1, 130.6, 129.9, 128.0, 125.7, 111.9, 105.1, 97.7, 57.4, 56.8.

HRMS (ESI) m/z: $[M + H]^+$ calcd for $C_{16}H_{14}N_5O_2S$, 340.0863; found, 340.0850.

2-Azido-4-(isopropylthio)-6,7-dimethoxyquinazoline (14d)

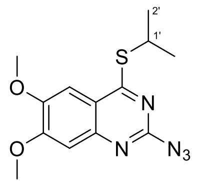

Method G: 2,4-Diazido-6,7-dimethoxyquinazoline (50 mg, 0.18 mmol, 1.0 equiv), K_2CO_3 (35 mg, 0.25 mmol, 1.35 equiv), isopropylthiol (45 mg, 0.22 mmol, 20 µL, 1.2 equiv). Yield: 28 mg, 50%. Colorless amorphous solid.

IR (UATR) v (cm^{-1}): 2959, 2920, 1958, 1618, 1600, 1535, 1306, 1250, 1203, 1041.

^{1}H NMR (500 MHz, $CDCl_3$) δ (ppm): 7.86 (s, 1H, H-C(Ar)), 7.44 (s, 1H, H-C(Ar)), 4.47 (septet, 1H, ^3J = 6.9 Hz, HC(1')), 4.15, 4.06 (2s, 6H, 2 x (-OCH_3)), 1.55 (d, 6H, ^3J = 6.9 Hz, 2 x H_3C(2')).

^{13}C NMR (126 MHz, $CDCl_3$) δ (ppm): 169.7, 156.2, 152.5, 149.9, 127.7, 112.5, 105.3, 97.6, 57.3, 56.6, 36.8, 22.9.

HRMS (ESI) m/z: $[M + H]^+$ calcd for $C_{13}H_{16}N_5O_2S$, 306.1019; found, 306.1007.

General procedure for the synthesis of 2-azido-6,7-dimethoxy-4-sulfonylquinazolines 15:

Tautomeric mixture of 2-azido-6,7-dimethoxy-4-tosylquinazoline and 7,8-dimethoxy-5-tosyltetrazolo[1,5-*a*]quinazoline (15a)

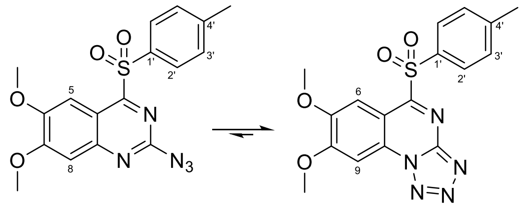

A solution of 2-azido-6,7-dimethoxy-4-(*p*-tolylthio)quinazoline (20 mg, 0.06 mmol, 1.0 equiv) and *m*CPBA (30 mg, 0.18 mmol, 3.0 equiv) in anhydrous DCM (3 mL) was stirred overnight under argon. The solvent was evaporated, recrystallized in EtOH (4 mL), and filtered over celite. The resulted precipitate was washed with cold EtOH (2 × 3 mL) and dissolved in DCM (4 × 5 mL). The solvent was evaporated and the solid dried in vacuum. Yield: 8 mg, 35%. Yellow crystalline solid.

IR (UATR) ν (cm^{-1}): 2922, 2853, 2134, 1618, 1551, 1498, 1391, 1249, 1209, 1085.

Tetrazole form from a 96:4 mixture in $CDCl_3$:

^1H NMR (500 MHz, $CDCl_3$) δ (ppm): 8.58 (s, 1H, H-C(9)), 8.04 (s, 1H, H-C(6)), 7.97 (d, 2H, ^3J = 8.0 Hz, H-C(2')), 7.46 (d, 2H, ^3J = 8.0 Hz, H-C(3')), 4.23, 4.17 (2s, 6H, 2 x (-OCH_3)), 2.53 (s, 3H, H_3-C(5')).

^{13}C NMR (126 MHz, $CDCl_3$) δ 163.6, 158.0, 150.7, 146.8, 133.8, 132.2, 131.5, 130.6, 130.1, 108.8, 106.6, 97.7, 57.6, 57.0, 22.1.

HRMS (ESI) m/z: $[M + H]^+$ calcd for $C_{17}H_{16}N_5O_4S$, 386.0918; found, 386.0902.

Tautomeric mixture of 2-azido-4-(dodecylsulfonyl)-6,7-dimethoxyquinazoline and 5-(dodecylsulfonyl)-7,8-dimethoxytetrazolo[1,5-*a*]quinazoline (15b)

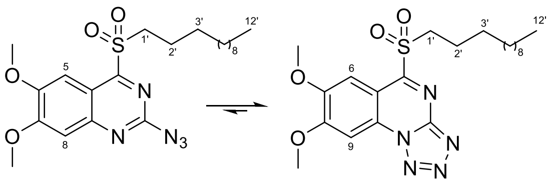

2-Azido-4-(dodecylthio)-6,7-dimethoxyquinazoline (56 mg, 0.13 mmol, 1.0 equiv), *m*CPBA (68 mg, 0.39 mmol, 3.0 equiv). Purified by silica gel column chromatography (Hexane/EtOAc 10→25%). Yield: 26 mg, 43%. Yellow amorphous solid.

IR (UATR) ν (cm^{-1}): 2918, 2848, 1617, 1597, 1520, 1391, 1376, 1249, 1148, 1085.

Tetrazole form from a 7:3 mixture in $CDCl_3$:

^{1}H NMR (500 MHz, $CDCl_3$) δ (ppm): 8.35 (s, 1H, H-C(9)), 8.05 (s, 1H, H-C(6)), 4.23, 4.10 (2s, 6H, 2 x (-OCH_3)), 3.86 (dd, 2H, ^3J = 10.8 Hz, ^3J = 5.3 Hz, H_2C(1')), 2.04 (quintet, 2H, ^3J = 7.9 Hz, H_2C(2')), 1.58 (quintet, 2H, ^3J = 7.2 Hz, H_2C(3')), 1.43–1.20 (m, 16H, 8 x H_2C), 0.87 (t, 3H, ^3J = 6.9 Hz, H_3C(12')).

^{13}C NMR (126 MHz, $CDCl_3$) δ (ppm): 162.4, 158.3, 150.7, 131.6, 108.5, 106.2, 102.0, 97.8, 57.6, 56.9, 51.7, 32.0, 29.7 (2C), 29.7, 29.5, 29.4, 29.2, 28.7, 22.8, 22.0, 14.3.

HRMS (ESI) m/z: $[M + H]^+$ calcd for $C_{22}H_{34}N_5O_4S$, 464.2326; found, 464.2306.

Tautomeric mixture of 2-azido-6,7-dimethoxy-4-(phenylsulfonyl)quinazoline and 7,8-dimethoxy-5-(phenylsulfonyl)tetrazolo[1,5-*a*]quinazoline (15c)

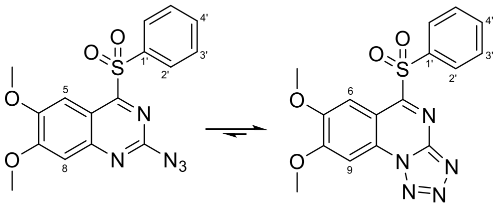

2-Azido-4-(phenylthio)-6,7-dimethoxyquinazoline (123 mg, 0.36 mmol, 1.0 equiv), *m*CPBA (190 mg, 1.1 mmol, 3.0 equiv). Yield: 95 mg, 70%. Yellow crystalline solid.

IR (UATR) ν (cm^{-1}): 2920, 2851, 2134, 1611, 1519, 1376, 1322, 1314, 1153, 1083.

Tetrazole form from a 7:3 mixture in $CDCl_3$:

^{1}H NMR (500 MHz, $CDCl_3$) δ (ppm): 8.57 (s, 1H, H-C(9)), 8.10 (d, 2H, ^{3}J = 7.3 Hz, H-C(2’)), 8.04 (s, 1H, H-C(6)), 7.80 (t, 1H, ^{3}J = 7.5 Hz, H-C(4’)), 7.67 (t, 2H, ^{3}J = 7.9 Hz , H-C(3’)), 4.23, 4.17 (2s, 6H, 2 x (-OCH_3)).

^{13}C NMR (126 MHz, $CDCl_3$) δ (ppm): 163.3, 158.1, 153.7, 150.7, 135.3, 134.7, 130.5, 130.1, 129.4, 129.1, 106.5, 97.8, 57.6, 57.0.

HRMS (ESI) m/z: $[M + H]^+$ calcd for $C_{16}H_{14}N_5O_4S$, 372.0761; found, 372.0747

Tautomeric mixture of 2-azido-4-(isopropylsulfonyl)-6,7-dimethoxyquinazoline and 5-(isopropylsulfonyl)-7,8-dimethoxytetrazolo[1,5-*a*]quinazoline (15d)

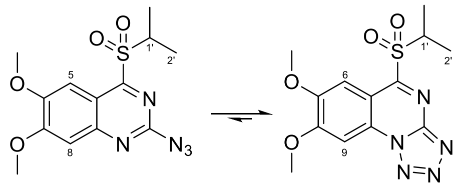

2-Azido-4-(isopropylthio)-6,7-dimethoxyquinazoline (15 mg, 0.05 mmol, 1.0 equiv), *m*CPBA (26 mg, 0.15 mmol, 3.0 equiv) Yield: 8 mg, 50%. Orange crystalline solid.

IR (UATR) ν (cm^{-1}): 2921, 2853, 1616, 1501, 1379, 1313, 1256, 1130, 1044, 998.

Tetrazole form from a 76:24 mixture in $CDCl_3$:

^{1}H NMR (500 MHz, $CDCl_3$) δ (ppm): 8.46 (s, 1H, H-C(9)), 8.06 (s, 1H, H-C(6)), 4.58 (septet, 1H, ^{3}J = 6.9 Hz, H-C(1’)), 4.23, 4.11 (2s, 6H, 2 x (-OCH_3)), 1.60 (d, 6H, ^{3}J = 6.9 Hz, 2 x H_3C(2’)).

^{13}C NMR (126 MHz, $CDCl_3$) δ (ppm): 161.8, 158.2, 153.6, 150.7, 131.7, 109.1, 106.4, 97.8, 57.6, 56.9, 51.7, 15.5.

HRMS (ESI) m/z: $[M + H]^+$ calcd for $C_{13}H_{16}N_5O_4S$, 338.0918; found, 338.0905.

Synthesis of 2-azido-6,7-dimethoxy-4-(pyrrolidin-1-yl)quinazoline (16)

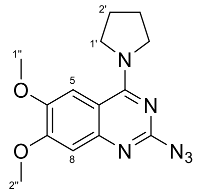

Pyrrolidine (0.18 ml, 156 mg, 2.2 mmol, 3.0 equiv) was added to a stirred suspension of 2,4-diazido-6,7-dimethoxyquinazoline (**13**, 200 mg, 0.73 mmol, 1.0 equiv) in DMF (3 mL) at 60 °C. After 15 minutes the reaction mixture was diluted with H_2O (15 mL), neutralized with 10% AcOH, cooled in the freezer, and filtered. The precipitate was washed with H_2O (2 × 3 mL) and cold EtOH (2 × 3 mL) and dried in vacuum. Yield: 204 mg, 93%. Colorless amorphous solid.

IR (UATR) ν (cm^{-1}): 3006, 2955, 2882, 2843, 1626, 1529, 1433, 1299, 1248, 1095.

^{1}H NMR (500 MHz, $CDCl_3$) δ (ppm): 7.78 (s, 1H, H-C(8)), 7.61 (s, 1H, H-C(5)), 4.11 (s, 3H, H_3-C(1'')), 4.03 - 3.97 (m, 7H, H_3-C(2''), 2 x H_2-C(1')), 2.13 – 2.07 (m, 4H, H_2-C(2')).

^{13}C NMR (126 MHz, $CDCl_3$) δ (ppm): 157.5, 154.2, 153.5, 148.0, 130.2, 108.0, 106.3, 98.1, 56.9, 56.5, 51.8, 25.9.

HRMS (ESI) m/z: $[M+H]^+$ calcd for $C_{14}H_{17}N_6O_2$, 301.1408; found, 301.1400.

Synthesis of 6,7-dimethoxy-4-(pyrrolidin-1-yl)-2-tosylquinazoline (18)

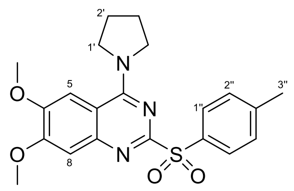

Pyrrolidine (60 µL, 0.73 mmol, 5.6 equiv) was added to a solution of 4-azido-6,7-dimethoxy-2-tosylquinazoline (**12a**, 50 mg, 0.13 mmol, 1.0 equiv) in anhydrous DMF (3 mL) under argon at 0 °C. After 0.5 h the reaction mixture was diluted with toluene (30 mL), washed with 11% NaCl (3 × 4 mL), back-extracted with toluene (3 × 3 mL),

dried over anhydrous Na_2SO_4. Purified by column chromatography (toluene/EtOAc 0 →50%). Yield: 24 mg, 44%. Colorless amorphous solid.

IR (UATR) ν (cm^{-1}): 2968, 2955, 2917, 2872, 1616, 1576, 1458, 1256, 1165, 1150, 1126, 1080.

^{1}H NMR (500 MHz, $CDCl_3$) δ (ppm): 8.05 (d, 2H, ^3J = 8.2 Hz, 2 x H-C(2')), 7.44 (s, 1H, H-C(Ar)), 7.41 (s, 1H, H-C(Ar)), 7.32 (d, 2H ^3J = 8.2 Hz, 2 x H-C(2'')), 3.99, 3.95 (2s, 6H, 2 x H_3C-O), 3.88 -3.82 (m, 4H, 2 x H_2-C(1')), 2.42 (s, 3H, H_3-C(3'')), 2.02 (m, 4H, 2 x H_2-C(2')).

^{13}C NMR (126 MHz, $CDCl_3$) δ 160.7, 159.5, 154.2, 148.8, 148.5, 144.4, 136.2, 129.8, 129.4, 110.4, 109.1, 104.5, 56.5, 56.2, 50.9, 25.8, 21.8.

HRMS (ESI) m/z: $[M+H]^+$ calcd for $C_{21}H_{24}N_3O_4S$, 414.1482, found, 414.1468.

General procedure for the synthesis of 2-amino-4-azido-6,7-dimethoxyquinazolines 17:

Tautomeric mixture of 4-azido-6,7-dimethoxy-2-(pyrrolidin-1-yl)quinazoline and 8,9-dimethoxy-5-(pyrrolidin-1-yl)tetrazolo[1,5-*c*]quinazoline (17a)

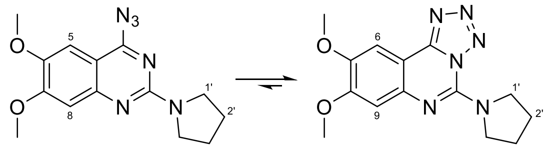

<u>Method H:</u> Pyrrolidine (0.14 mL, 118 mg, 1.66 mmol, 3.0 equiv) was added to a stirred solution of 4-azido-6,7-dimethoxy-2-tosylquinazoline (213 mg, 0.55 mmol, 1.0 equiv) in $CHCl_3$ (5 mL) at room temperature. Then, the mixture was diluted with $CHCl_3$ (25 mL), washed with H_2O (3 × 3 mL) and brine (3 mL) and the organic layer was dried over anhydrous Na_2SO_4 and evaporated and dried in vacuum. Yield: 154 mg, 93%. Light yellow crystalline solid.

IR (UATR) ν (cm^{-1}): 2960, 2883, 2836, 1617, 1496, 1348, 1238, 1214, 1022.

^{1}H NMR (500 MHz, $CDCl_3$) δ (ppm): 7.69 (s, 1H, H-C(Ar)), 7.08 (s, 1H, H-C(Ar)), 4.14–4.09 (m, 4H, 2×H_2-C(1’)), 4.01, 4.01 (2s, 6H, 2×H_3C-O), 2.11–2.07 (m, 4H, 2×H_2-C(2’))

^{13}C NMR (126 MHz, $CDCl_3$) δ (ppm): 154.7, 151.1, 147.3, 142.3, 140.7, 106.4, 103.9, 103.7, 56.5, 56.4, 50.2, 25.6.

HRMS (ESI) m/z: $[M + H]^+$ calcd for $C_{14}H_{17}N_6O_2$, 301.1408; found, 301.1398.

Tautomeric mixture of 4-azido-6,7-dimethoxy-2-(piperidin-1-yl)quinazoline and 8,9-dimethoxy-5-(piperidin-1-yl)tetrazolo[1,5-*c*]quinazoline (17b)

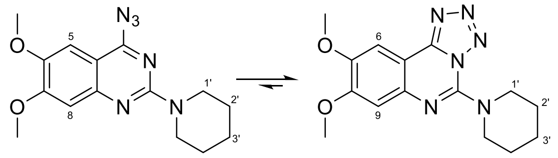

Method H: 4-Azido-6,7-dimethoxy-2-tosylquinazoline (100 mg, 0.26 mmol, 1.0 equiv), piperidine (0.05 mL, 45 mg, 0.5 mmol, 2.0 equiv), MeCN (10 mL), 1.5 h. Recrystallized from EtOH (5 mL). Yield: 60 mg, 73%. Light pink crystalline solid.

IR (KBr) ν (cm^{-1}): 2959, 2929, 2862, 2834, 1624, 1611, 1497, 1261, 1215, 1125, 1013.

Tetrazole form from an 80:20 mixture in $CDCl_3$:

^{1}H NMR (500 MHz, $CDCl_3$) δ (ppm): 7.77 (s, 1H, H-C(Ar)), 7.17 (s, 1H, H-C(Ar)), 4.04, 4.02 (2s, 6H, 2×H_3C-O), 4.02–3.99 (m, 4H, 2×H_2-C(1’)), 1.85–1.80 (m, 4H, 2 × H_2-C(2’)), 1.79–1.75 (m, 2H, H_2-C(3’)).

^{13}C NMR (126 MHz, $CDCl_3$) δ (ppm): 154.6, 151.2, 148.2, 142.3, 141.1, 107.0, 104.8, 103.8, 56.6, 56.4, 49.3, 25.8, 24.7.

HRMS (ESI) m/z: $[M + H]^+$ calcd for $C_{15}H_{19}N_6O_2$, 315.1564; found, 315.1556.

Tautomeric mixture of 4-azido-6,7-dimethoxy-2-(morpholin-1-yl)quinazoline and 8,9-dimethoxy-5-(morpholin-1-yl)tetrazolo[1,5-*c*]quinazoline (17c)

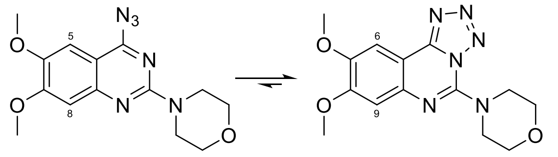

Method H: 4-Azido-6,7-dimethoxy-2-tosylquinazoline (100 mg, 0.26 mmol, 1.0 equiv), morpholine (0.05 mL, 45 mg, 0.5 mmol, 2.0 equiv), MeCN (10 ml), 16 h, recrystallized from EtOH (18 mL). Yield: 61 mg, 75%. Light green crystalline solid.

IR (KBr) ν (cm^{-1}): 2999, 2976, 2909, 2865, 1626, 1613, 1498, 1360, 1263, 1245, 1115, 1016.

Tetrazole form from a 53:47 mixture in $CDCl_3$:

^1H NMR (500 MHz, $CDCl_3$) δ (ppm): 7.03 (s, 1H, H-C(Ar)), 6.91 (s, 1H, H-C(Ar)), 4.09–4.06 (m, 4H, 2×H_2-C(1')), 3.98, 3.93 (2s, 6H, 2×H_3C-O), 3.97–3.94 (m, 4H, H_2-C(2')).

^{13}C NMR (126 MHz, $CDCl_3$) δ (ppm): 159.9, 156.7, 151.3, 146.9, 141.9, 107.2, 103.8, 101.8, 66.6, 56.6, 56.5, 48.3.

HRMS (ESI) m/z: $[M + H]^+$ calcd for $C_{14}H_{17}N_6O_3$, 317.1357; found, 317.1348.

Tautomeric mixture of 4-azido-6,7-dimethoxy-2-(4-methylpiperazin-1-yl)quinazoline and 8,9-dimethoxy-5-(4-methylpiperazin-1-yl)tetrazolo[1,5-*c*]quinazoline (17d)

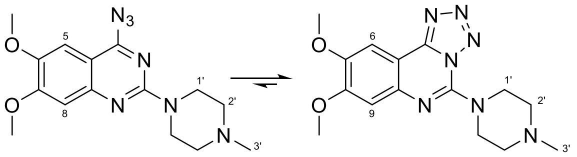

<u>Method H</u>: 4-Azido-6,7-dimetoxy-2-tosylquinazoline (200 mg, 0.52 mmol, 1.0 equiv), *N*-methylpiperazine (0.12 mL, 104 mg, 1.0 mmol, 2.0 equiv), MeCN (20 mL), 16 h, recrystallized from a mixture of *n*-propanol (2 mL) and MeCN (3 mL). Yield: 131 mg, 77%. Light green crystalline solid.

IR (UATR) ν (cm^{-1}): 3003, 2966, 2932, 2860, 2802, 1615, 1498, 1433, 1261, 1242, 1212, 1004.

Tetrazole form from a 6:4 mixture in $CDCl_3$:

^{1}H NMR (500 MHz, $CDCl_3$) δ (ppm): 7.78 (s, 1H, H-C(Ar)), 7.19 (s, 1H, H-C(Ar)), 4.13 – 4.07 (m, 4H, 2 x H_2-C(1')), 4.04, 4.03 (2s, 6H, 2 x H_3C-O), 2.67 (t, 4H, ^3J = 5.0 Hz, 2 x H_2-C(2')), 2.39 (s, 3H, H_3-C(3')).

^{13}C NMR (126 MHz, $CDCl_3$) δ (ppm): 158.0, 154.7, 151.2, 148.5, 140.9, 107.1, 105.2, 103.8, 56.6, 56.4, 54.8, 47.9, 46.2.

HRMS (ESI) m/z: $[M + H]^+$ calcd for $C_{15}H_{20}N_7O_2$, 330.1673; found 330.1664.

Tautomeric mixture of (4-(4-azido-6,7-dimethoxyquinazolin-2-yl)piperazin-1-yl)(tetrahydrofuran-2-yl)methanone and (4-(8,9-dimethoxytetrazolo[1,5-*c*]quinazolin-5-yl)piperazin-1-yl)(tetrahydrofuran-2-yl)methanone (17e)

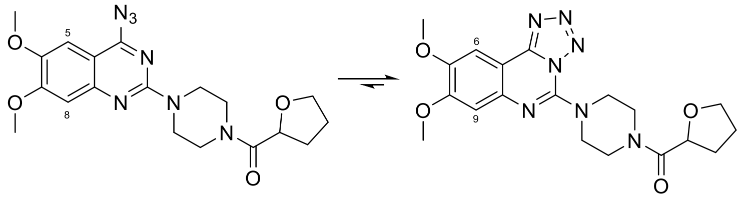

Method I: Piperazin-1-yl(tetrahydrofuran-2-yl)methanone (160 mg, 0.86 mmol, 3.0 equiv) was added to a suspension of 4-azido-6,7-dimethoxy-2-tosylquinazoline (111 mg, 0.29 mmol, 1.0 equiv) in DMSO (2 mL) at room temperature, monitored by HPLC. After 16 h, the reaction mixture was diluted with toluene (30 mL) and washed with 11% NaCl solution (8 × 3 mL) and brine (2 × 3 mL) and the organic layer was dried over anhydrous Na_2SO_4. Purified by column chromatography (toluene/MeCN 10→30%). Yield: 95 mg, 80%. Red amorphous solid.

Method J: Anhydrous DMSO (3 mL) was added to a mixture of 2,4-dichloro-6,7-dimethoxyquinazoline (100 mg, 0.39 mmol, 1.0 equiv) and sodium 4-methylbenzene sulfinate (72 mg, 0.41 mmol, 1.05 equiv). Upon full starting material conversion, a 0.5 M solution of NaN_3 in anhydrous DMSO (0.6 mL, 0.31 mmol, 0.8 equiv) was added to the reaction mixture over 2 hours. Piperazin-1-yl(tetrahydrofuran-2-yl)methanone (142 mg, 0.78 mmol, 2 equiv), dissolved in anhydrous DMSO (1 mL), was added to the

reaction mixture and stirred at room temperature overnight. The reaction mixture was diluted with DCM (30 mL), washed with 11% NaCl (8 × 3 mL), back-extracted with DCM (2 × 5 mL) and the combined organic layer washed with brine (5 mL) and then dried over anhydrous Na_2SO_4. The solvent was evaporated, and the solid residue was purified by column chromatography (toluene/MeCN 10→30%). Yield: 64 mg, 41%. Red amorphous solid.

IR (UATR) ν (cm^{-1}): 3468, 3090, 2912, 2877, 2131, 1613, 1502, 1430, 1243, 1215, 1012.

Azide form from a 6:4 mixture in $CDCl_3$:

^1H NMR (500 MHz, $CDCl_3$) δ (ppm): 7.02 (s, 1H, H-C(Ar)), 6.91 (s, 1H, H-C(Ar)), 4.66 (t, 1H, ^3J = 6.5 Hz, H-C(3')), 4.26–4.12 (m, 1H), 4.07–3.58 (m, 15H), 2.45–2.29 (m, 1H), 2.13–1.87 (m, 3H).

^{13}C NMR (126 MHz, $CDCl_3$) δ (ppm): 170.2, 157.8, 156.8, 151.3, 147.0, 106.6, 104.9, 101.8, 76.2, 69.3, 56.4, 56.2, 45.5, 44.0, 42.2, 28.5, 25.9.

HRMS (ESI) m/z: $[M + H]^+$ calcd for $C_{19}H_{24}N_7O_4$, 414.1884; found, 414.1867.

Tautomeric mixture of (4-(4-azido-6,7-dimethoxyquinazolin-2-yl)piperazin-1-yl)(furan-2-yl)methanone and (4-(8,9-dimethoxytetrazolo[1,5-*c*]quinazolin-5-yl)piperazin-1-yl)(furan-2-yl)methanone (17f)

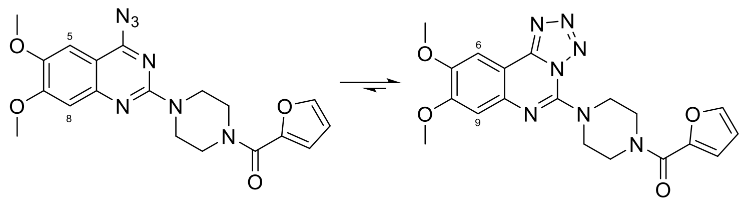

Method I: Piperazin-1-yl(furan-2-yl)methanone (170 mg, 0.94 mmol, 3.0 equiv), 4-azido-6,7-dimethoxy-2-tosylquinazoline (121 mg, 0.31 mmol, 1.0 equiv), DMSO (2 mL), 16 h. Purified by column chromatography (Toluene/MeCN 10→30%). Yield: 96 mg, 75%. Red amorphous solid.

Method J: Anhydrous DMSO (3 mL), 2,4-dichloro-6,7-dimethoxyquinazoline (100 mg, 0.39 mmol, 1.0 equiv), sodium 4-methylbenzenesulfinate (72 mg, 0.41 mmol, 1.05 equiv), a 0.5 M solution of NaN_3 in anhydrous DMSO (0.6 mL, 0.31 mmol, 0.8 equiv), furan-2-yl(piperazin-1-yl)methanone (139 mg, 0.78 mmol, 2.0 equiv). Purified by column chromatography (Toluene/MeCN 10→30%). Yield: 77 mg, 49%. Red amorphous solid.

IR (UATR) ν (cm^{-1}): 3140, 2922, 2851, 2132, 1614, 1477, 1431, 1262, 1215, 1007.

^{1}H NMR (500 MHz, $CDCl_3$) δ (ppm): 7.54–7.50 (m, 1H, H-C(4’)), 7.12–7.03 (m, 2H), 6.92 (s, 1H, H-C(Ar)), 6.52 (m, 1H, H-C(5’)), 4.20–3.86 (m, 14H).

^{13}C NMR (126 MHz, $CDCl_3$) δ (ppm): 160.0, 157.8, 156.9, 151.3, 148.1, 147.0, 143.9, 116.8, 111.5, 107.3, 106.6, 104.9, 101.8, 56.4, 56.2, 48.1, 44.3.

HRMS (ESI) m/z: $[M + H]^+$ calcd for $C_{19}H_{20}N_7O_4$, 410.1571; found, 410.1554.

General procedure for the synthesis of 4-amino-6,7-dimethoxy-2-(piperazin-1-yl)quinazoline hydrochlorides 19:

(4-(4-Amino-6,7-dimethoxyquinazolin-2-yl)piperazin-1-yl)(tetrahydrofuran-2-yl)methanone hydrochloride, terazosin hydrochloride (19a)

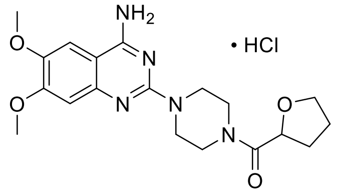

H_2 was bubbled through a suspension of (4-(4-azido-6,7-dimethoxyquinazolin-2-yl)piperazin-1-yl)(tetrahydrofuran-2-yl)methanone (**17e**, 50 mg, 0.12 mmol 1.0 equiv) and 10% Pd/C (5 mg, 10 wt %) in MeOH (30 mL) for 3 h at rt. The reaction mixture was filtered through celite and evaporated. The solid residue was dissolved in DCM (30 mL) and washed with sat. $NaHCO_3$ (3 × 5 mL), the organic layer was evaporated, dissolved in MeOH (5 mL) and acidified with 4 M HCl in iPrOH (3 mL), evaporated,

recrystallized from iPrOH (5 mL), and dried in vacuum. Yield: 38 mg, 71%. Colorless crystalline solid.

^{1}H- and ^{13}C-NMR data correspond to literature [3–5].

(4-(4-Amino-6,7-dimethoxyquinazolin-2-yl)piperazin-1-yl)(furan-2-yl)methanone hydrochloride, prazosin hydrochloride (19b)

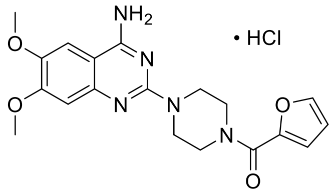

(4-(4-Azido-6,7-dimethoxyquinazolin-2-yl)piperazin-1-yl)(furan-2-yl)methanone (**17f**, 50 mg, 0.12 mmol 1.0 equiv) and 10% Pd/C (5 mg, 10 wt %) in MeOH (30 mL). Yield: 37 mg, 73%. Colorless crystalline solid.

^{1}H- and ^{13}C-NMR data correspond to literature [5–7].

General procedure for the synthesis of 2-amino-6,7-dimethoxy-4-(1,2,3-triazol-1-yl)quinazolines 20:

6,7-Dimethoxy-4-(4-phenyl-1*H*-1,2,3-triazol-1-yl)-2-(pyrrolidin-1-yl)quinazoline (20a)

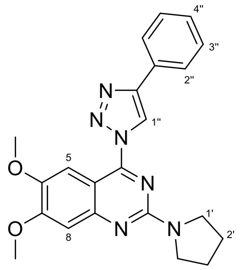

Phenylacetylene (0.04 mL, 34 mg, 0.33 mmol, 2.0 equiv) was added to a suspension of 4-azido-6,7-dimethoxy-2-(pyrrolidine-1-yl)quinazoline (**17a**, 50 mg, 0.17 mmol, 1.0 equiv), tetrakis(acetonitrile)copper(I) hexafluorophosphate (3.1 mg, 0.01 mmol, 5 mol %) and TBTA (4.4 mg, 0.01 mmol, 5 mol %) in toluene (10 mL) and stirred at

110 °C until completion. After 96 h the solvent was evaporated, the solid residue dissolved in DCM (30 mL) and washed with sat. EDTA solution (3 × 10 mL), the organic layer was dried over anhydrous Na_2SO_4 and evaporated. The product was purified by column chromatography (toluene/MeCN 1→5%). Yield: 42 mg, 63%. Orange amorphous solid.

IR (UATR) ν (cm^{-1}): 3155, 2966, 2927, 2866, 1626, 1594, 1458, 1393, 1232, 1027, 1016.

^{1}H NMR (500 MHz, $CDCl_3$) δ (ppm): 8.88 (s, 1H, H-C(1”)), 8.41 (s, 1H, H-C(Ar)), 7.99 (d, 2H, ^3J = 7.4 Hz, 2 x H-C(2”)), 7.49 (t, 2H, ^3J = 7.6 Hz, 2 x H-C(3”)), 7.40 (t, 1H, ^3J = 7.4 Hz, H-C(4”)), 7.03 (s, 1H, H-C(Ar)), 4.03, 4.00 (2s, 6H, 2 x H_3C-O), 3.74–3.68 (m, 4H, 2 x H_2-C(1’)), 2.05 (quintet, 4H, ^3J = 3.4 Hz, 2 x H_2-C(2’)).

^{13}C NMR (126 MHz, $CDCl_3$) δ (ppm): 156.8, 156.5, 155.0, 151.7, 147.4, 146.7, 130.1, 129.1, 128.8, 126.1, 119.6, 105.3, 105.0, 104.8, 56.4, 56.3, 47.0, 25.7.

HRMS (ESI) m/z: $[M+H]^+$ calcd for $C_{22}H_{23}N_6O_2$, 403.1877; found, 403.1869.

6,7-Dimethoxy-4-(4-(4-cyanophenyl)-1*H*-1,2,3-triazol-1-yl)-2-(4-methylpiperazin-1-yl)quinazoline (20b)

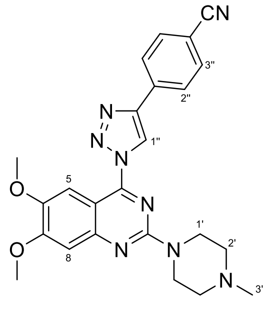

4-Cyanophenylacetylene (0.04 mL, 34 mg, 0.33 mmol, 2.0 equiv), 4-azido-6,7-dimethoxy-2-(4-methylpiperazin-1-yl)quinazoline (**17d**, 50 mg, 0.15 mmol, 1.0 equiv), tetrakis(acetonitrile)copper(I) hexafluorophosphate (3.0 mg, 0.01 mmol, 5 mol %), TBTA (4.2 mg, 0.01 mmol, 5 mol %), toluene (10 mL), 36 h. Purified by column chromatography (DCM/MeOH 1→4%). Yield: 22 mg, 32%. Yellow amorphous solid.

IR (UATR) ν (cm^{-1}): 1599, 1492, 1450, 1355, 1239, 1214, 1174, 1094, 1024, 1005.

^{1}H NMR (500 MHz, $CDCl_3$) δ (ppm): 8.93 (s, 1H, H-C(1'')), 8.31 (s, 1H), 8.10 (d, 2H, ^{3}J = 8.2 Hz, 2 x H-C(2'')), 7.78 (d, 2H, ^{3}J = 8.2 Hz, 2 x H-C(3'')), 7.03 (s, 1H, H-C(Ar)), 4.04, 4.00 (2s, 6H, 2 x H_3C-O), 3.98–3.94 (m, 4H, 2 x H_2-C(1')), 2.57 (t, 4H, ^{3}J = 5.2 Hz, 2 x H_2-C(2')), 2.39 (s, 3H, H_3-C(3')).

^{13}C NMR (126 MHz, $CDCl_3$) δ (ppm): 157.6, 157.2, 154.9, 151.7, 148.2, 145.1, 134.4, 133.0, 126.6, 120.8, 118.8, 112.3, 105.7, 105.2, 104.4, 56.5, 56.3, 55.1, 46.5, 44.3.

HRMS (ESI) m/z: $[M+H]^+$ calcd for $C_{24}H_{25}N_8O_2$, 457.2095; found, 457.2083.

Synthesis of 6,7-dimethoxy-2-(4-methylpiperazin-1-yl)-4-[(triphenyl-phosphoronylidene)amino]quinazoline (21)

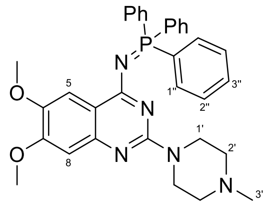

A solution of 4-azido-6,7-dimethoxy-2-(4-methylpiperazin-1-yl)quinazoline (50 mg, 0.15 mmol, 1.0 equiv) and triphenylphosphine (80 mg, 0.30 mmol, 2 equiv) in toluene (5 mL) was stirred at 110 °C until completion, as monitored by HPLC. After 1 h, the solvent was evaporated, and the oily residue was recrystallized from a 1:1 mixture of Et_2O and hexane (30 mL) and dried in vacuum. Yield: 77 mg, 90%. Yellow crystalline solid.

IR (UATR) ν (cm^{-1}): 2958, 2931, 2886, 2840, 2790, 1621, 1570, 1534, 1484, 1421, 1286, 1210, 1182, 1113, 1010.

^{1}H NMR (500 MHz, $CDCl_3$) δ 7.83 (s, 1H, H-C(Ar)), 7.81–7.71 (m, 6H), 7.58–7.49 (m, 3H, 3 x H-C(3'')), 7.44 (td, 6H, ^{3}J = 7.8 Hz, ^{4}J =3.1 Hz, 6 x H-C(2'')), 6.84 (s, 1H, H-

C(Ar)), 4.01 (s, 3H), 3.94 (s, 3H), 3.33 (t, 4H, ^3J = 5.1 Hz, 2 x H_2-C(1')), 2.22 (s, 3H, H_3-C(3')), 2.16 (t, 4H, ^3J = 5.2 Hz, 2 x H_2-C(2')).

^{13}C NMR (126 MHz, $CDCl_3$) δ (ppm): 166.5 (D, $^2J_{C\text{-}P}$ = 7 Hz), 158.8, 154.3, 149.4 (d, $^3J_{C\text{-}P}$ = 3 Hz), 145.1, 133.1 (D, $^3J_{C\text{-}P}$ = 10 Hz), 132.0 (D, $^4J_{C\text{-}P}$ = 3 Hz), 129.8 (D, $^1J_{C\text{-}P}$ = 100 Hz), 128.6 (D, $^2J_{C\text{-}P}$ = 12 Hz), 111.5 (D, $^3J_{C\text{-}P}$ = 22 Hz), 105.9, 105.0, 56.1, 56.0, 55.3, 46.4, 43.7.

^{31}P NMR (202 MHz, $CDCl_3$) δ (ppm): 18.80.

HRMS (ESI) m/z: $[M+H]^+$ calcd for $C_{33}H_{35}N_5O_2P$, 564.2523; found, 564.2484.

Copies of ^{1}H, ^{13}C, and ^{31}P NMR spectra for all compounds

4-Azido-2-tosylquinazoline (**4a**)

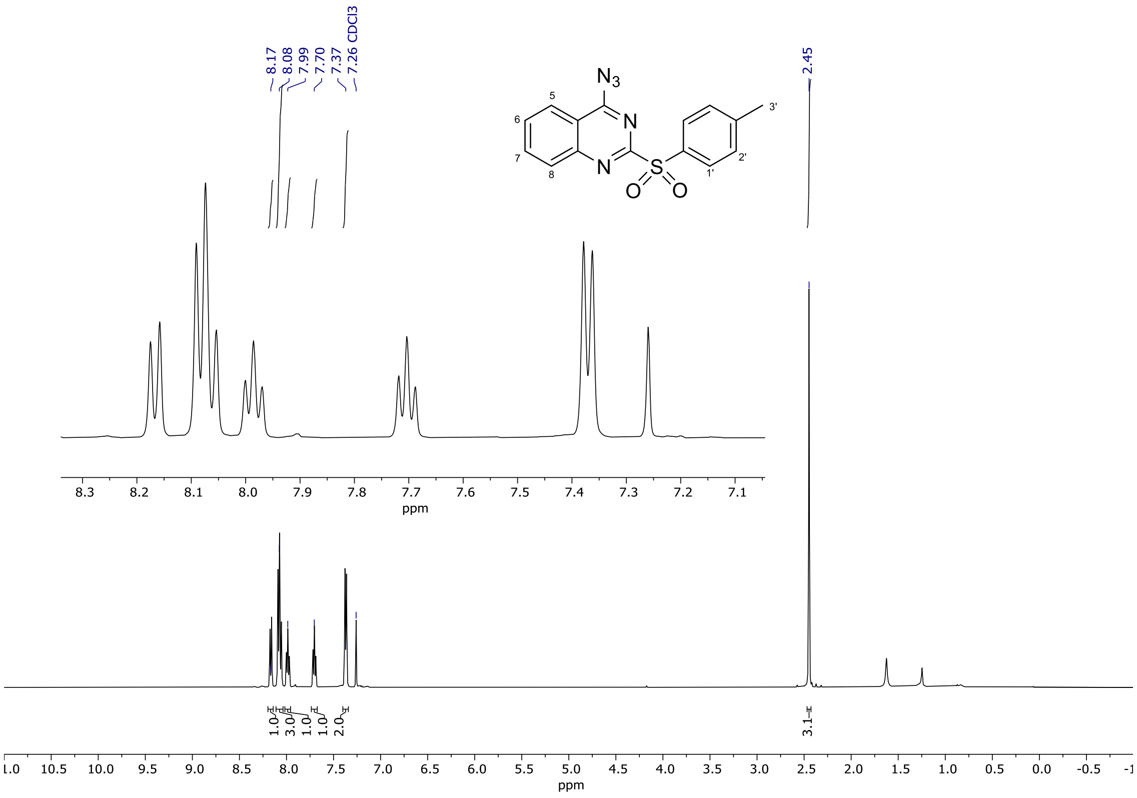

Figure S1: ^{1}H NMR (500 MHz, $CDCl_3$) spectrum.

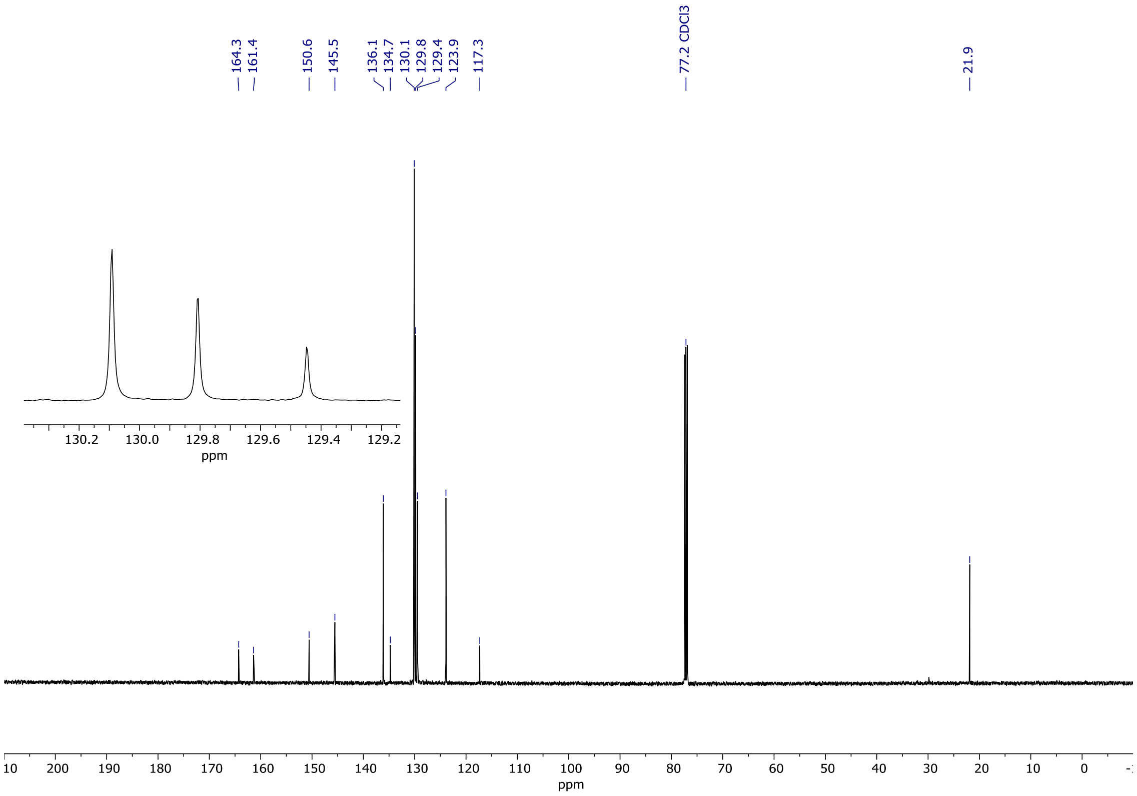

Figure S2: ^{13}C NMR (126 MHz, $CDCl_3$) spectrum.

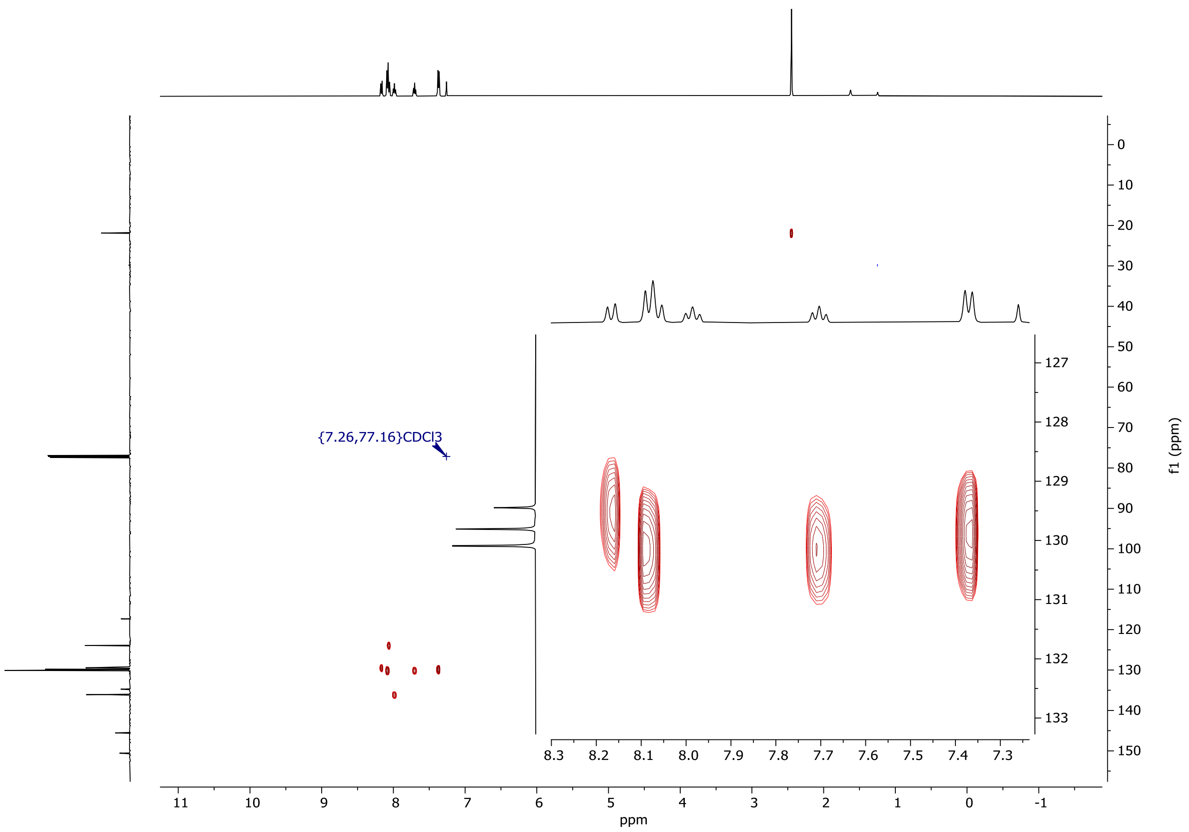

Figure S3: ^{1}H-^{13}C HSQC spectrum.

4-Azido-8-methoxy-2-tosylquinazoline (**4b**)

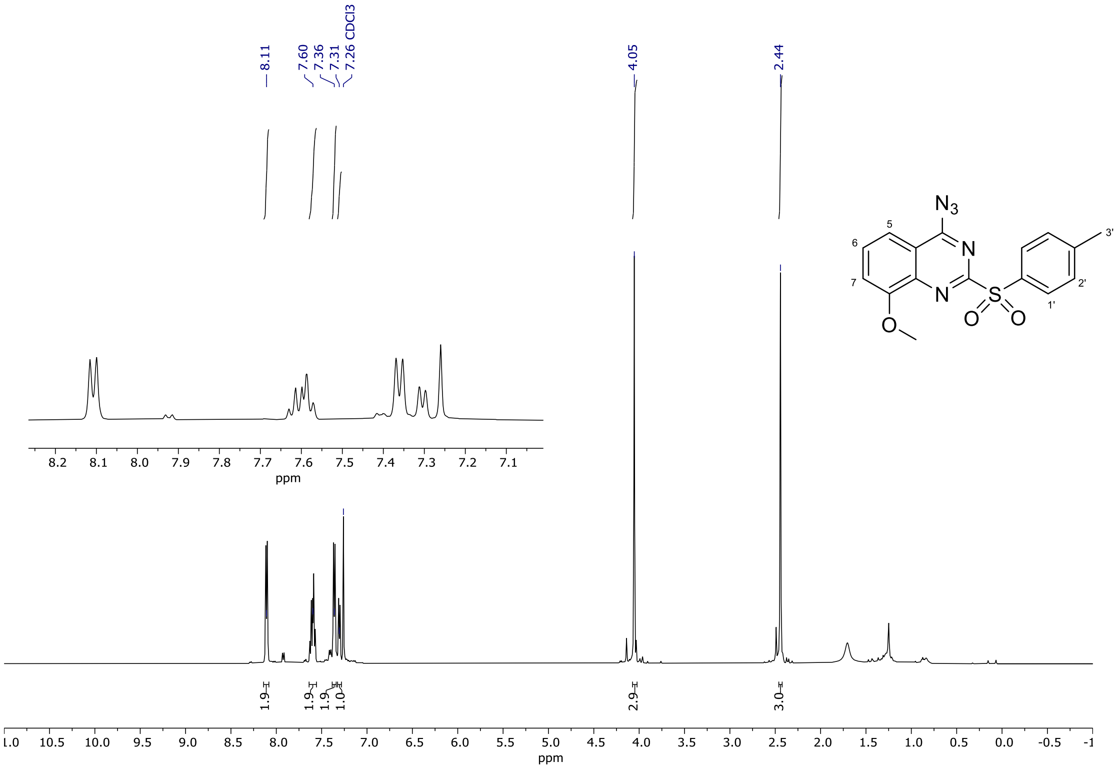

Figure S4: ^{1}H NMR (500 MHz, $CDCl_3$) spectrum.

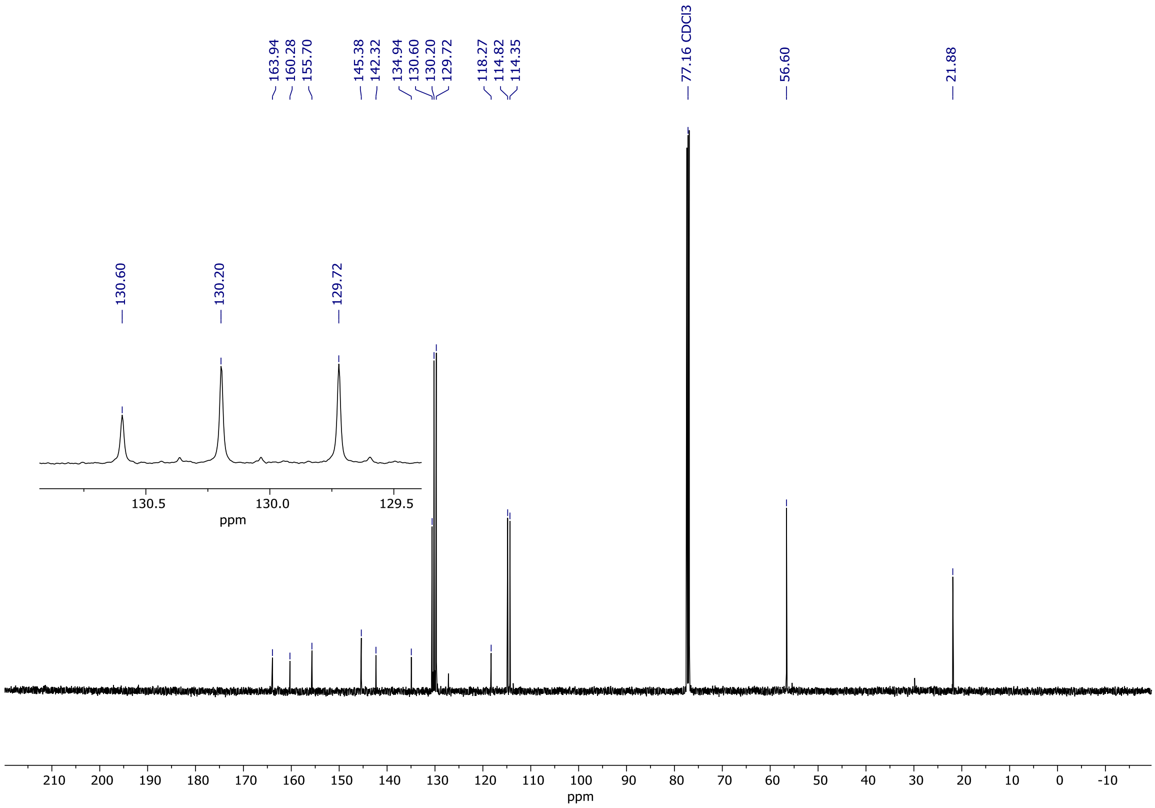

Figure S5: ^{13}C NMR (126 MHz, $CDCl_3$) spectrum.

4-Azido-6-bromo-2-tosylquinazoline (**4c**)

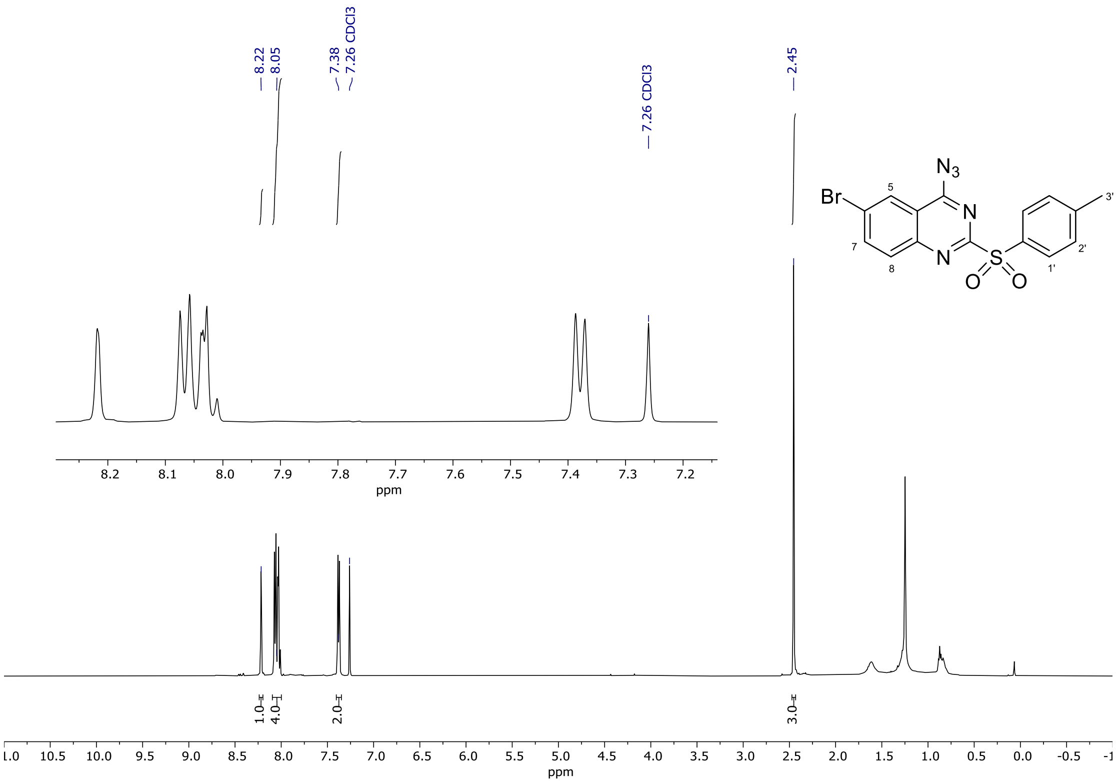

Figure S6: ^{1}H NMR (500 MHz, $CDCl_3$) spectrum.

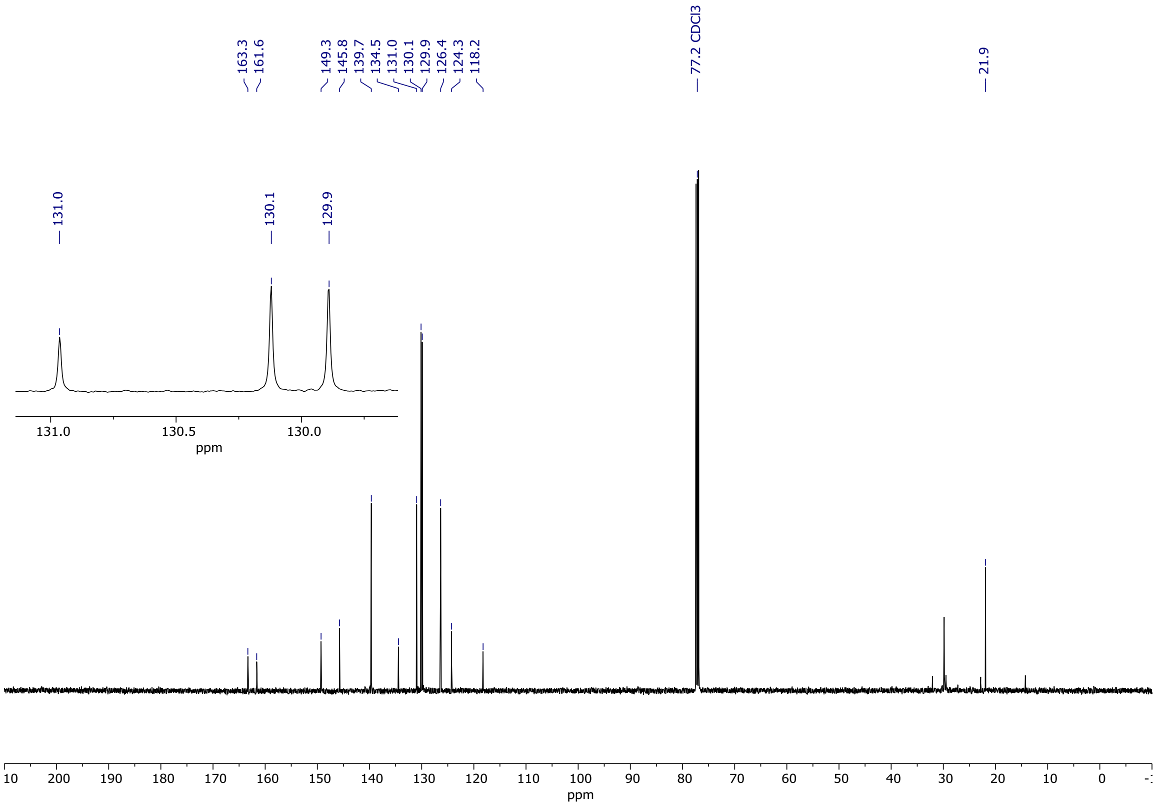

Figure S7: ^{13}C NMR (126 MHz, $CDCl_3$) spectrum.

2-Chloro-4-(dodecylsulfonyl)-6,7-dimethoxyquinazoline (**8b**)

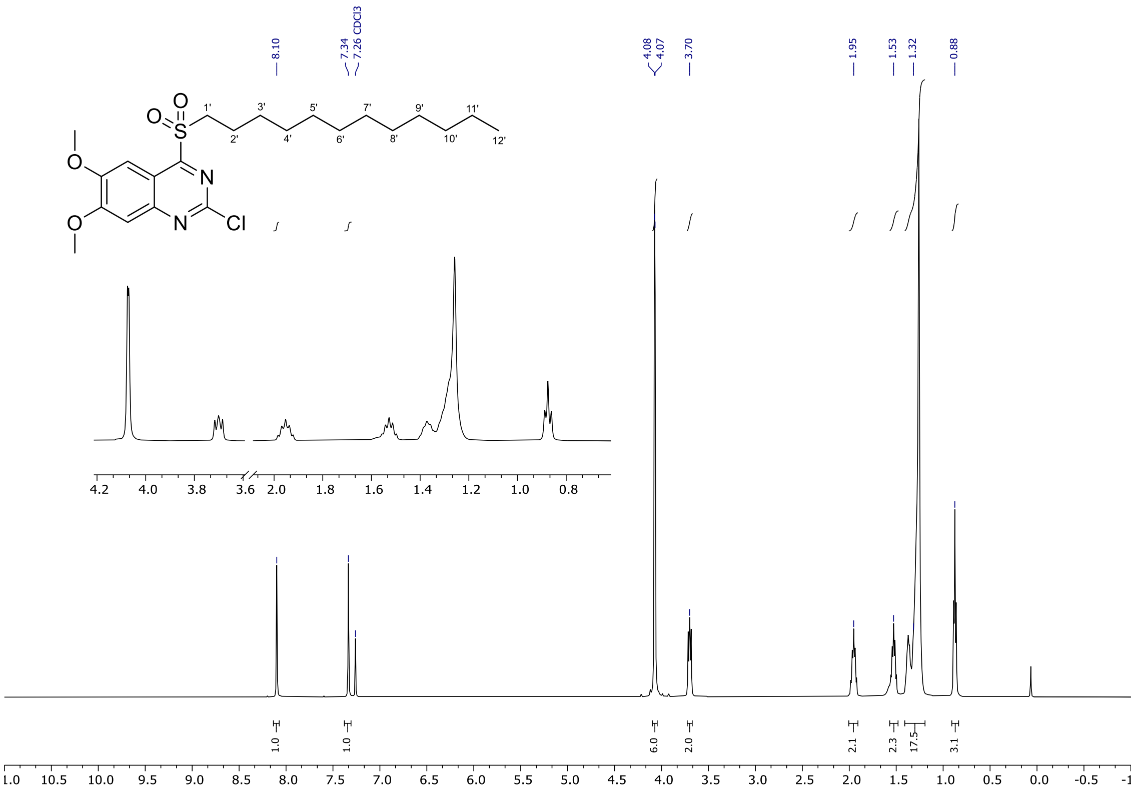

Figure S8: ^{1}H NMR ($CDCl_3$, 500 MHz) spectrum.

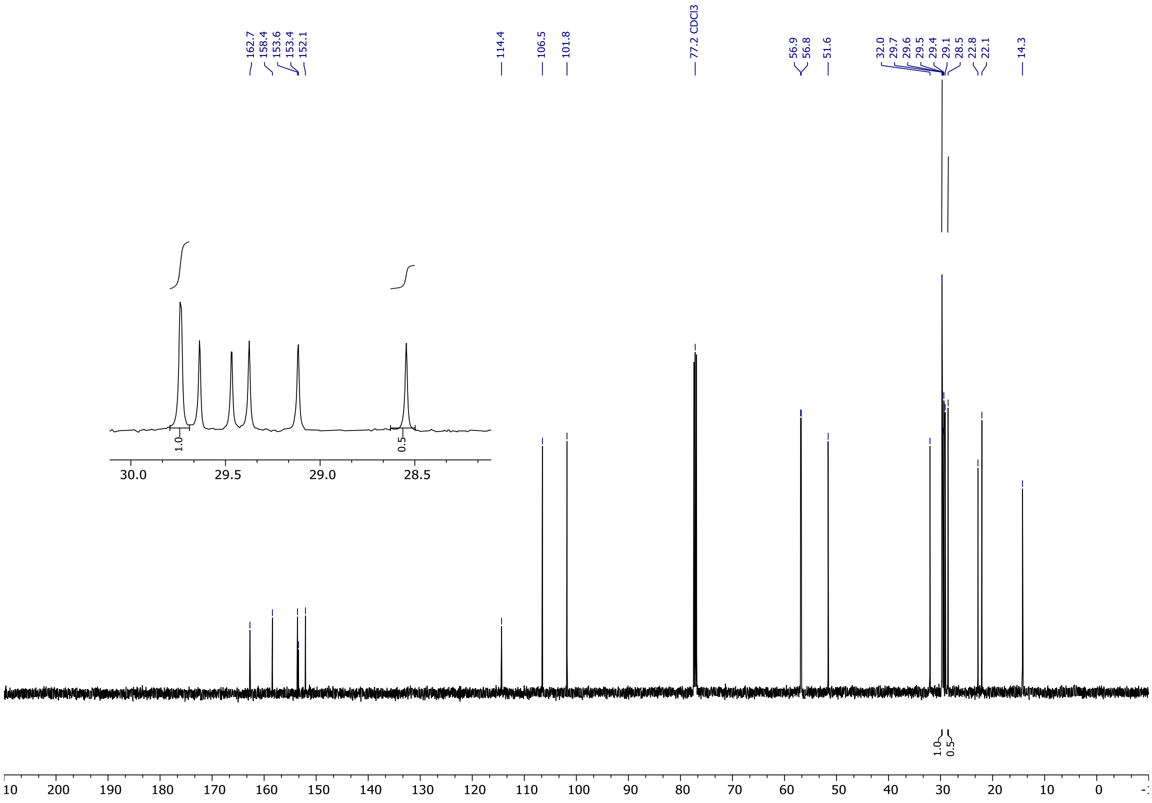

Figure S9: ^{13}C NMR ($CDCl_3$, 126 MHz) spectrum.

2-Chloro-6,7-dimethoxy-4-(phenylsulfonyl)quinazoline (**8c**)

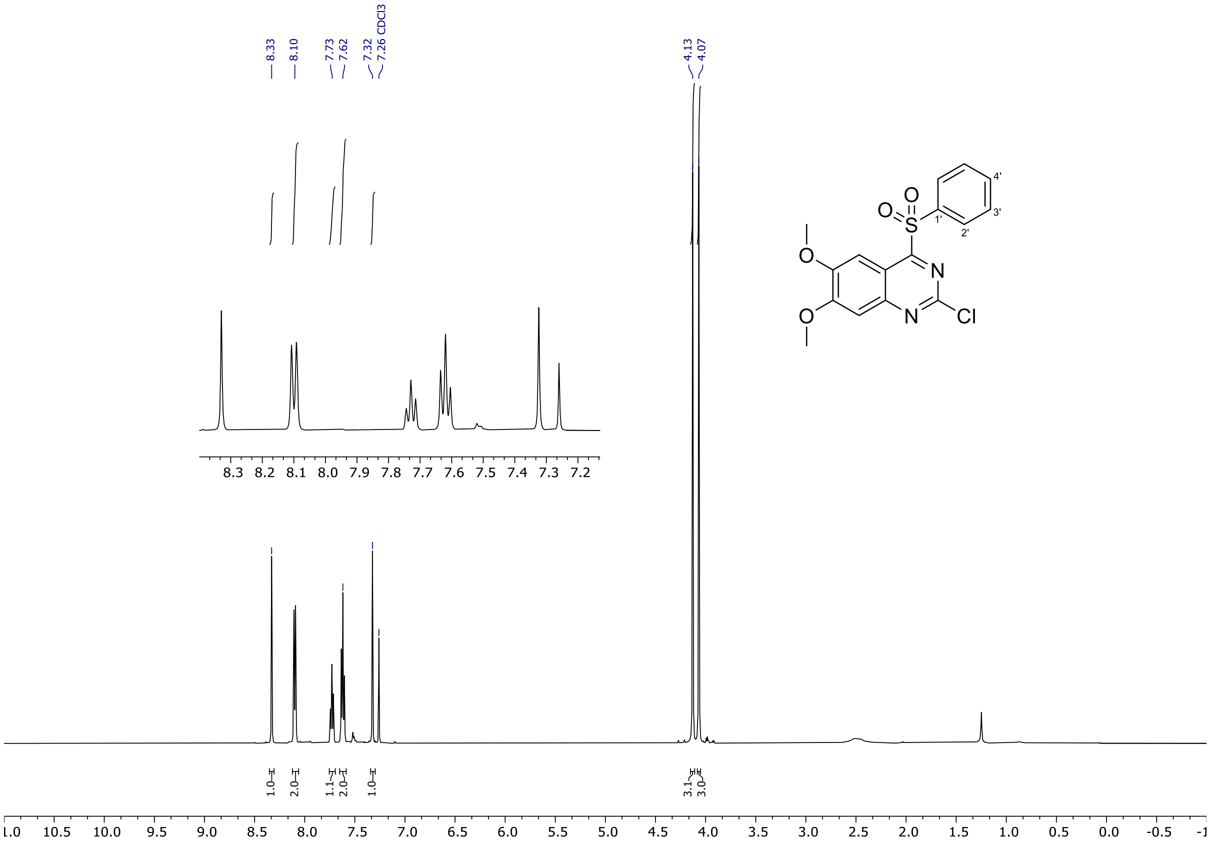

Figure S10: ^{1}H NMR ($CDCl_3$, 500 MHz) spectrum.

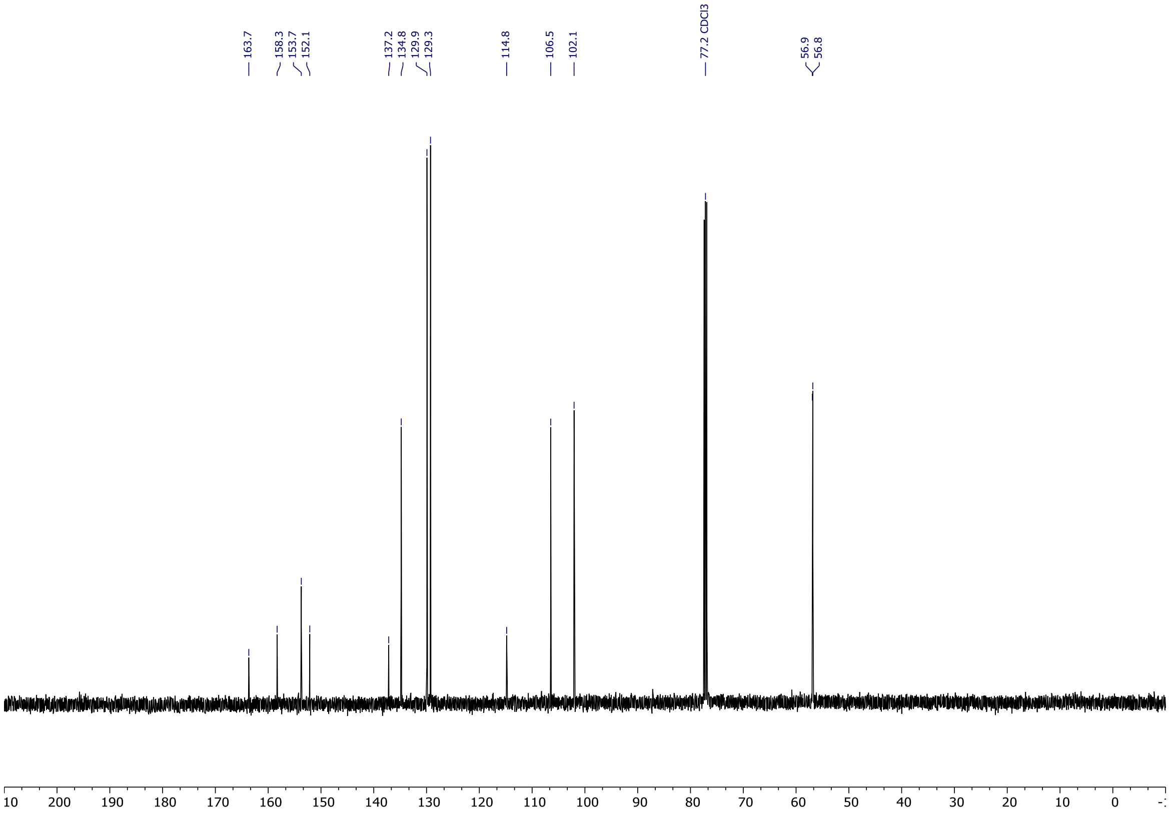

Figure S11: ^{13}C NMR (126 MHz, $CDCl_3$) spectrum.

2-Chloro-4-(isopropylsulfonyl)-6,7-dimethoxyquinazoline (**8d**)

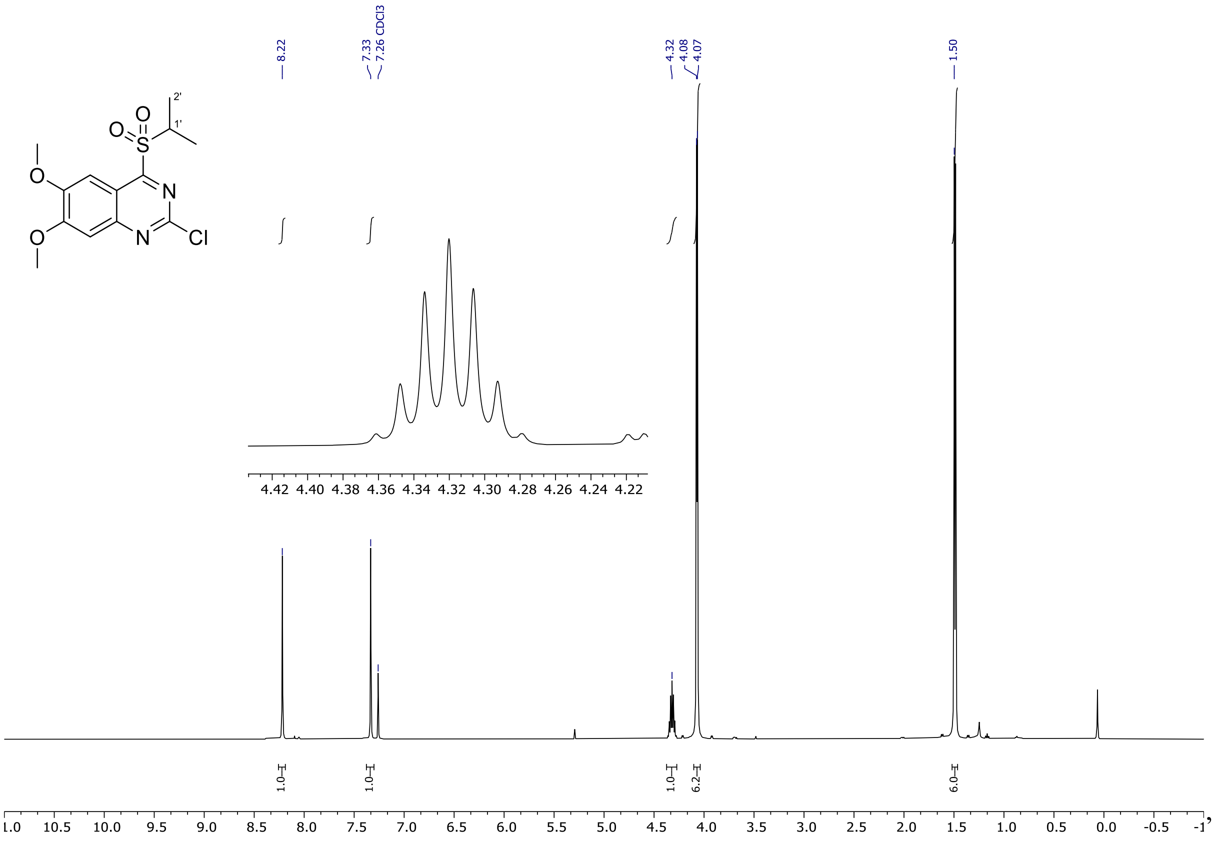

Figure S12: ^{1}H NMR (500 MHz, $CDCl_3$) spectrum.

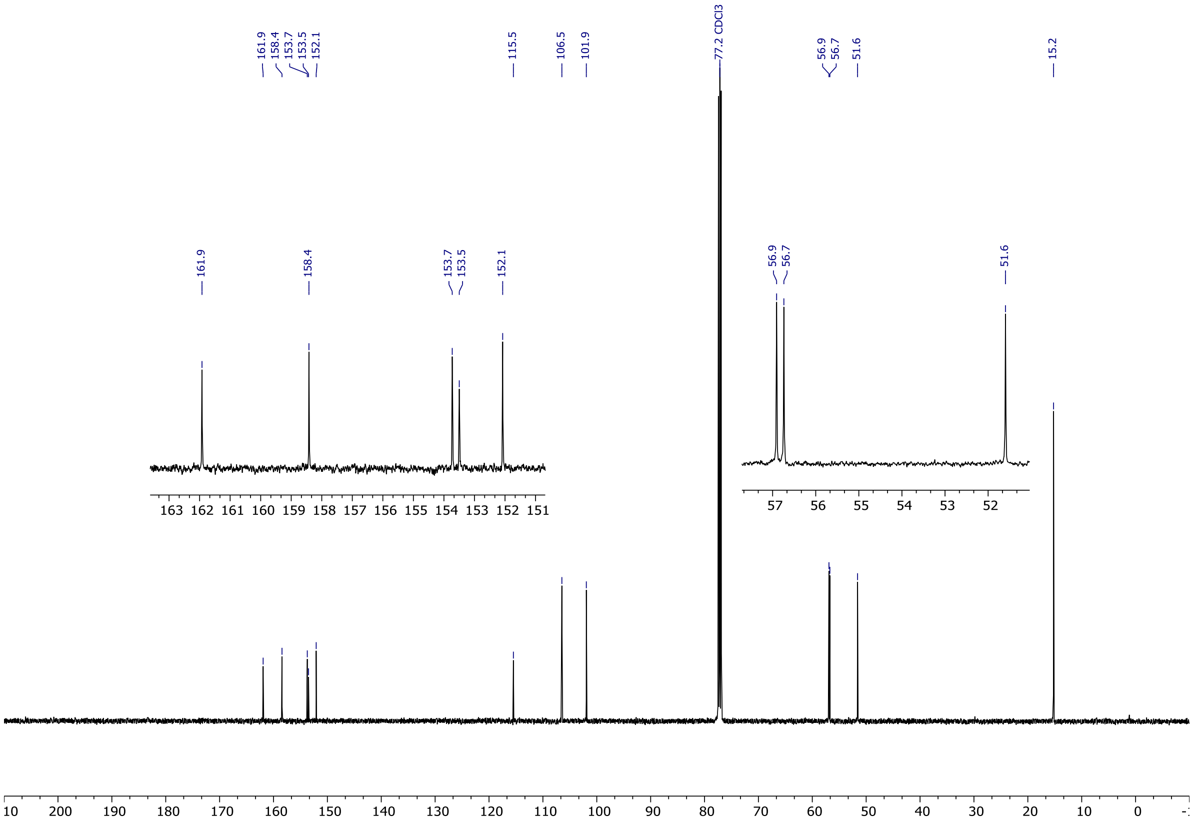

Figure S13: ^{13}C NMR (126 MHz, $CDCl_3$) spectrum.

2-Chloro-6,7-dimethoxy-4-(*p*-tolylthio)quinazoline (**10a**)

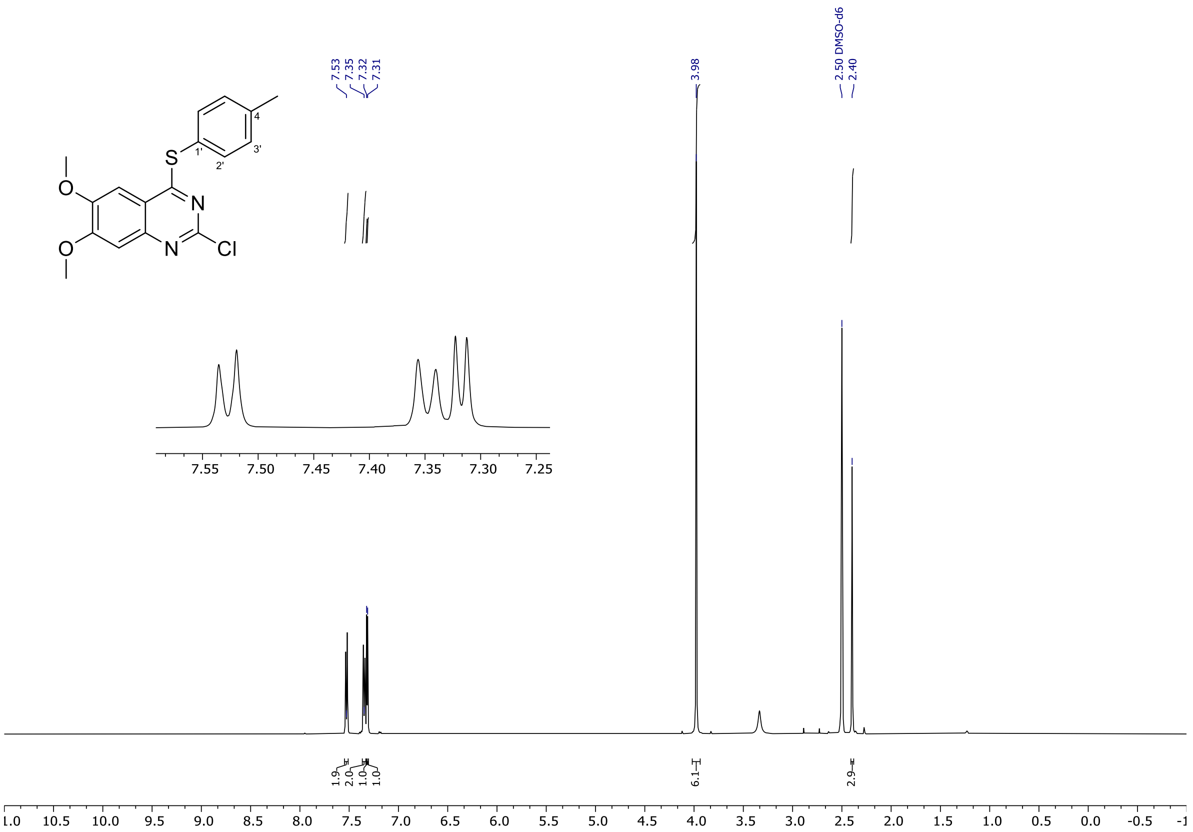

Figure S14: ^{1}H NMR (500 MHz, DMSO-d_6) spectrum.

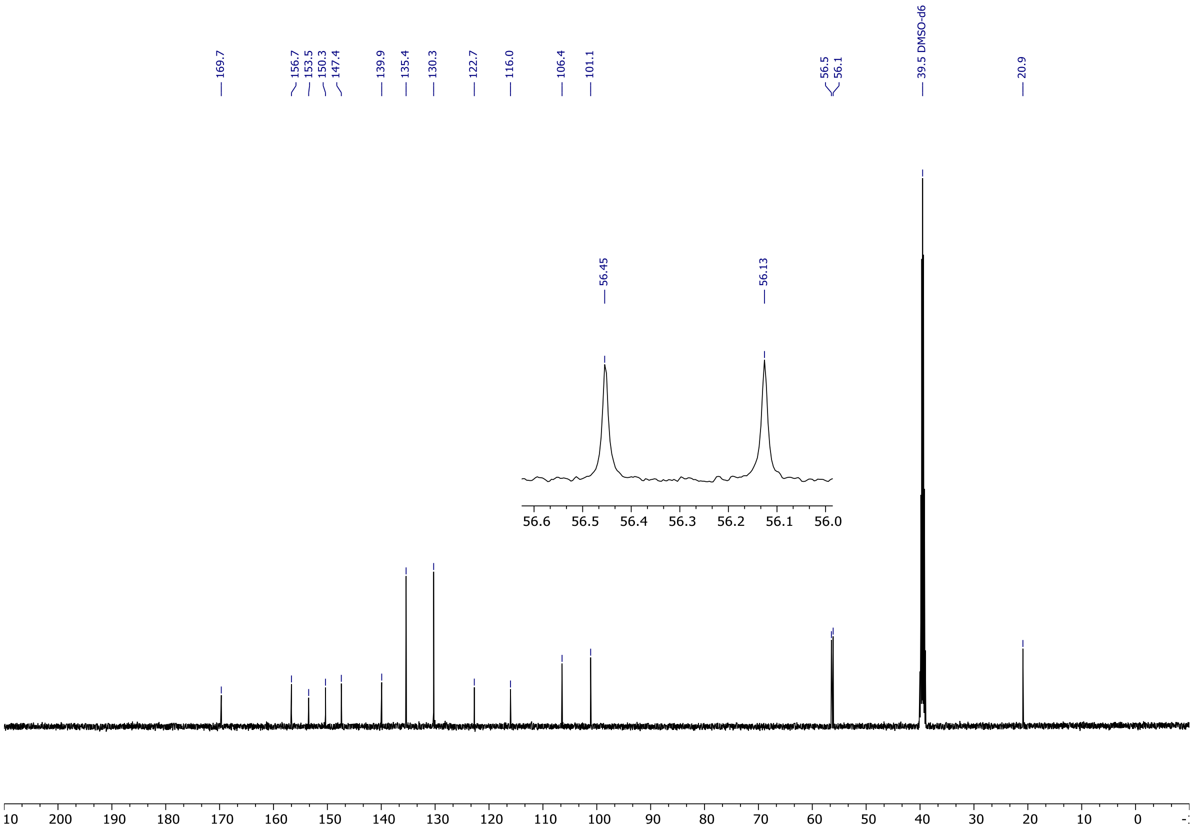

Figure S15: ^{13}C NMR (126 MHz, DMSO-d_6) spectrum.

2-Chloro-4-(dodecylthio)-6,7-dimethoxyquinazoline (**10b**)

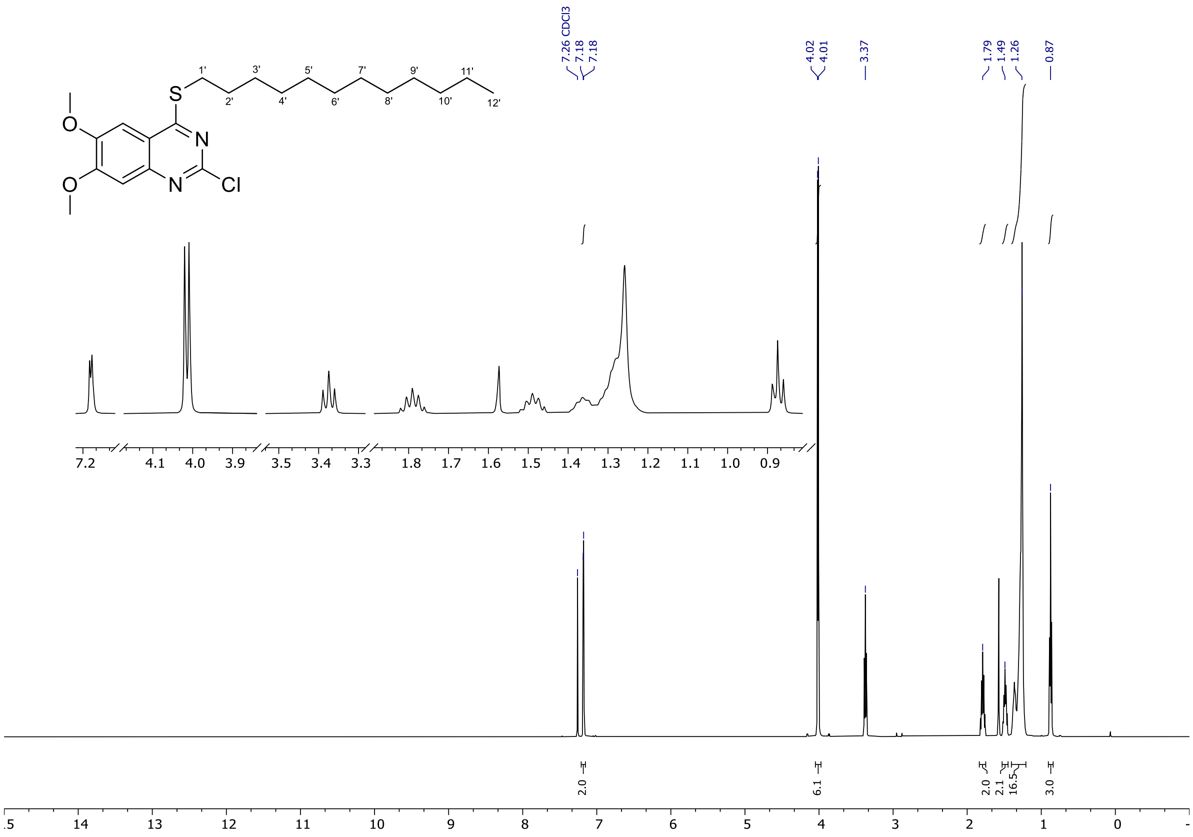

Figure S16: ^{1}H NMR (500 MHz, $CDCl_3$) spectrum.

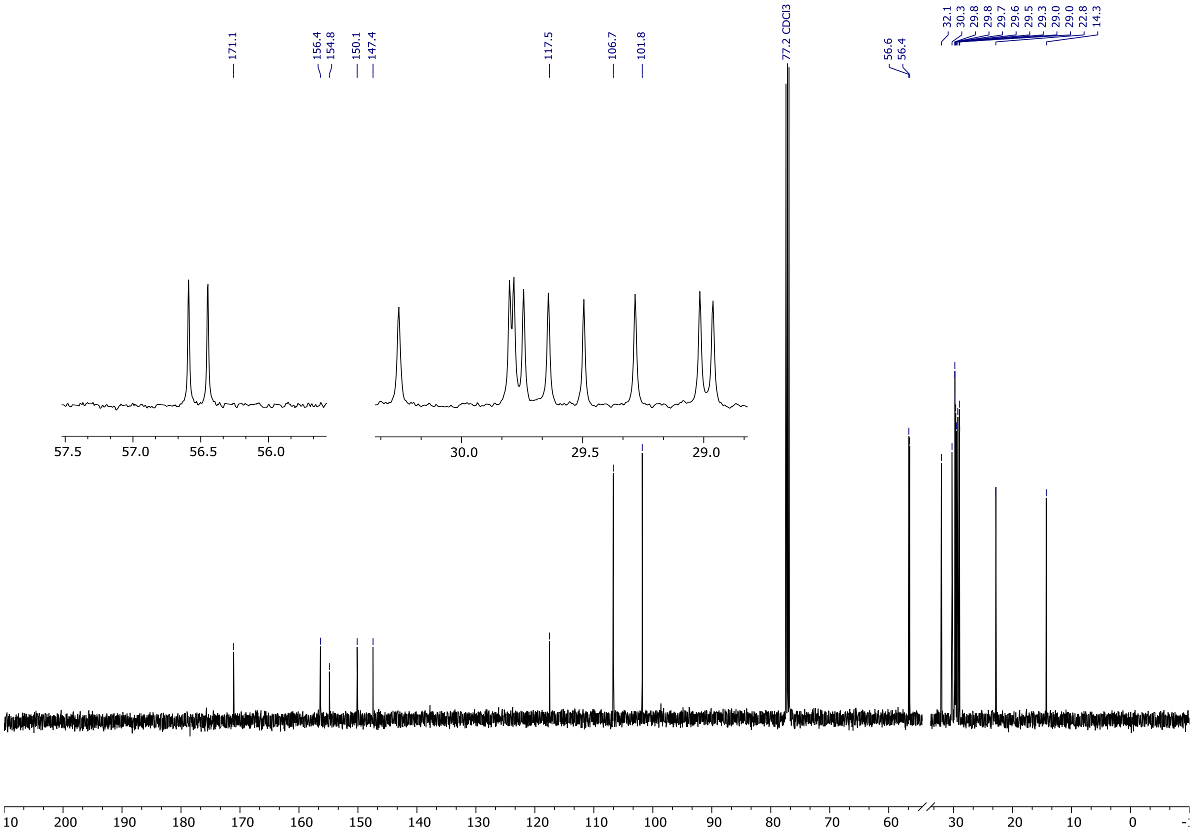

Figure S17: ^{13}C NMR (126 MHz, $CDCl_3$) spectrum.

2-Chloro-6,7-dimethoxy-4-(phenylthio)quinazoline (**10c**)

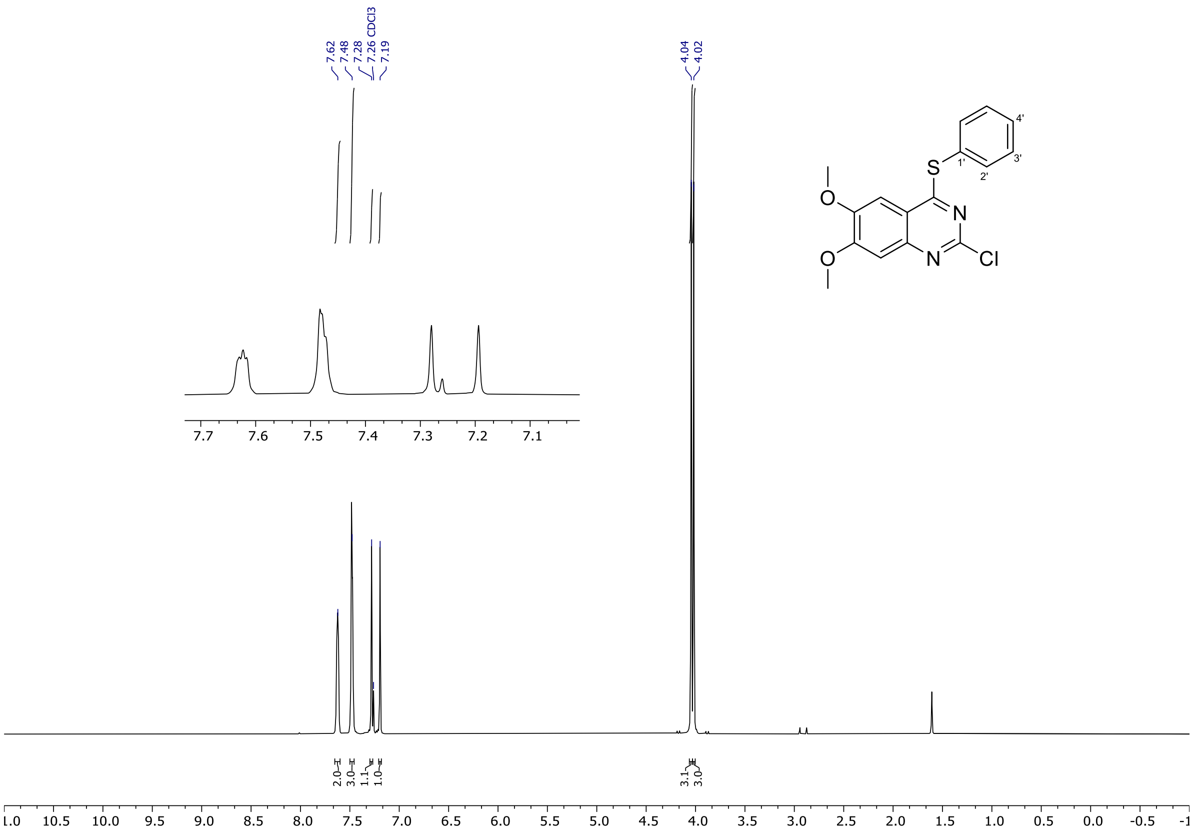

Figure S18: ^{1}H NMR (500 MHz, $CDCl_3$) spectrum.

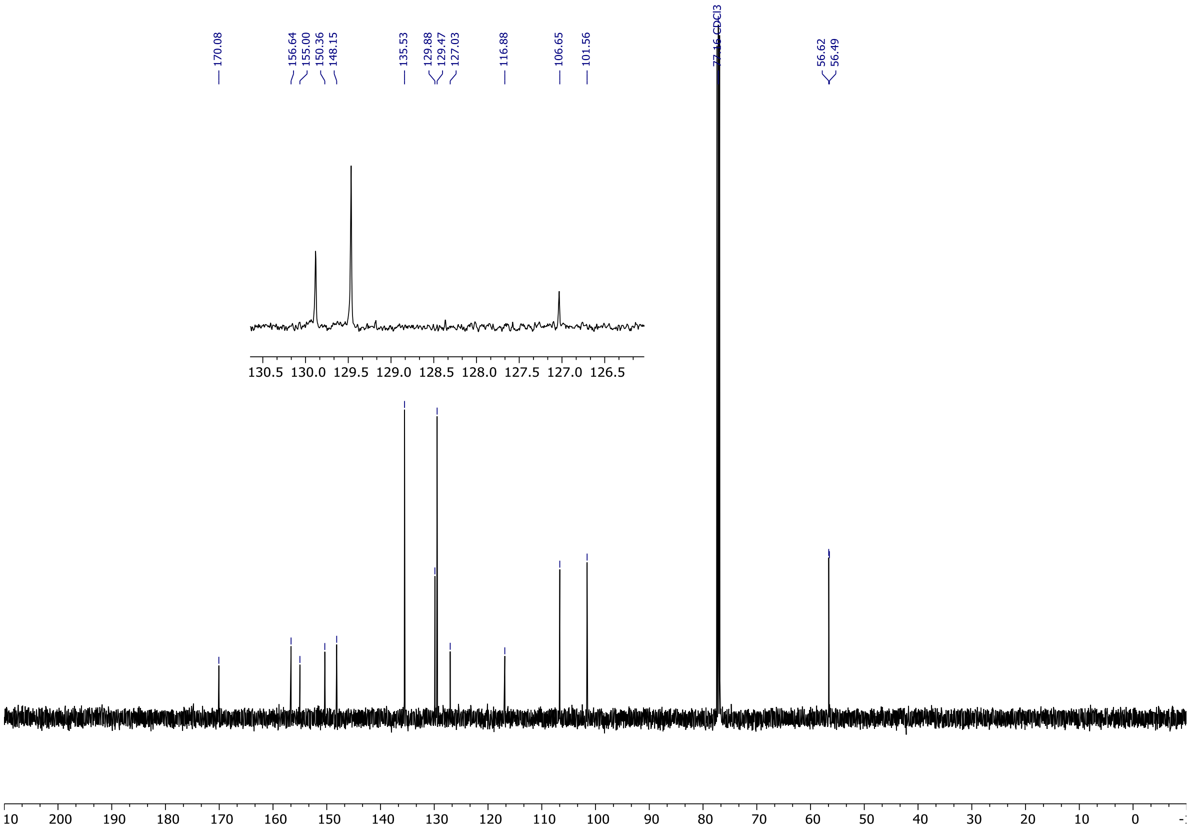

Figure S19: ^{13}C NMR (126 MHz, $CDCl_3$) spectrum.

2-Chloro-4-(isopropylthio)-6,7-dimethoxyquinazoline (**10d**)

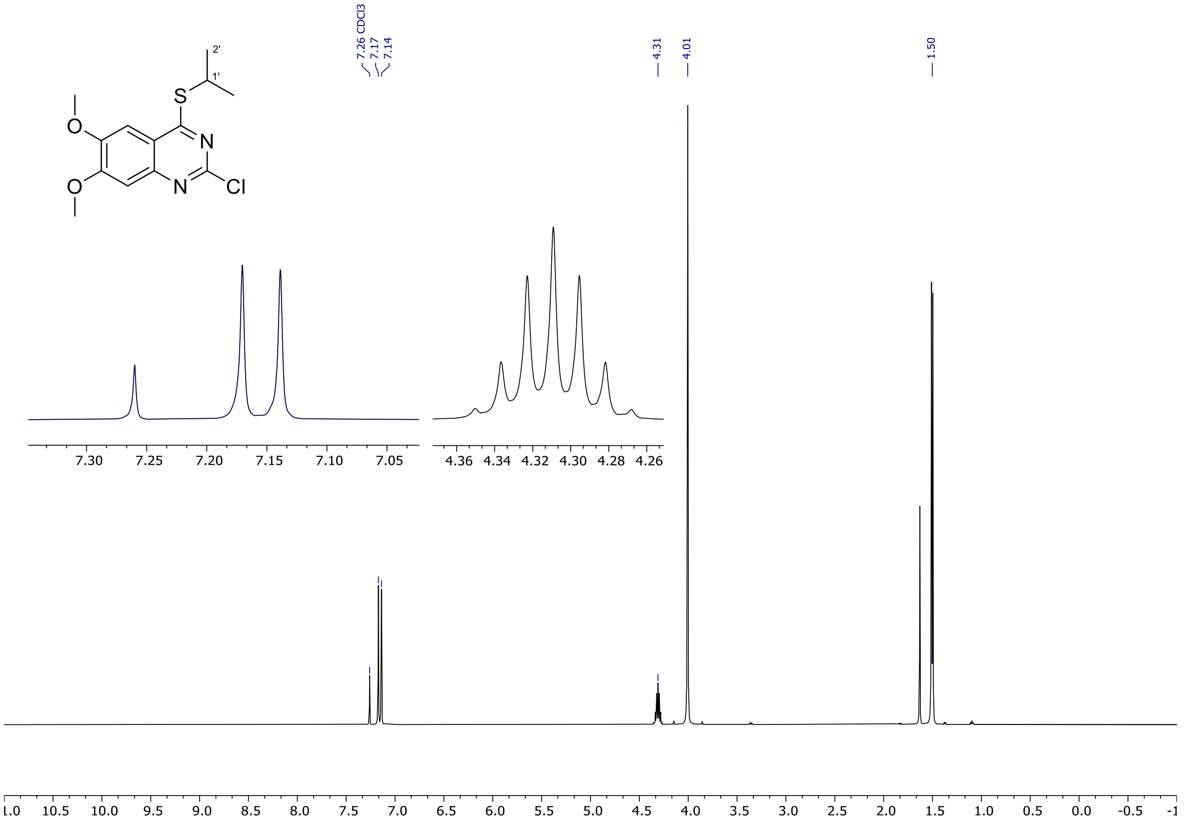

Figure S20: ^{1}H NMR (500 MHz, $CDCl_3$) spectrum.

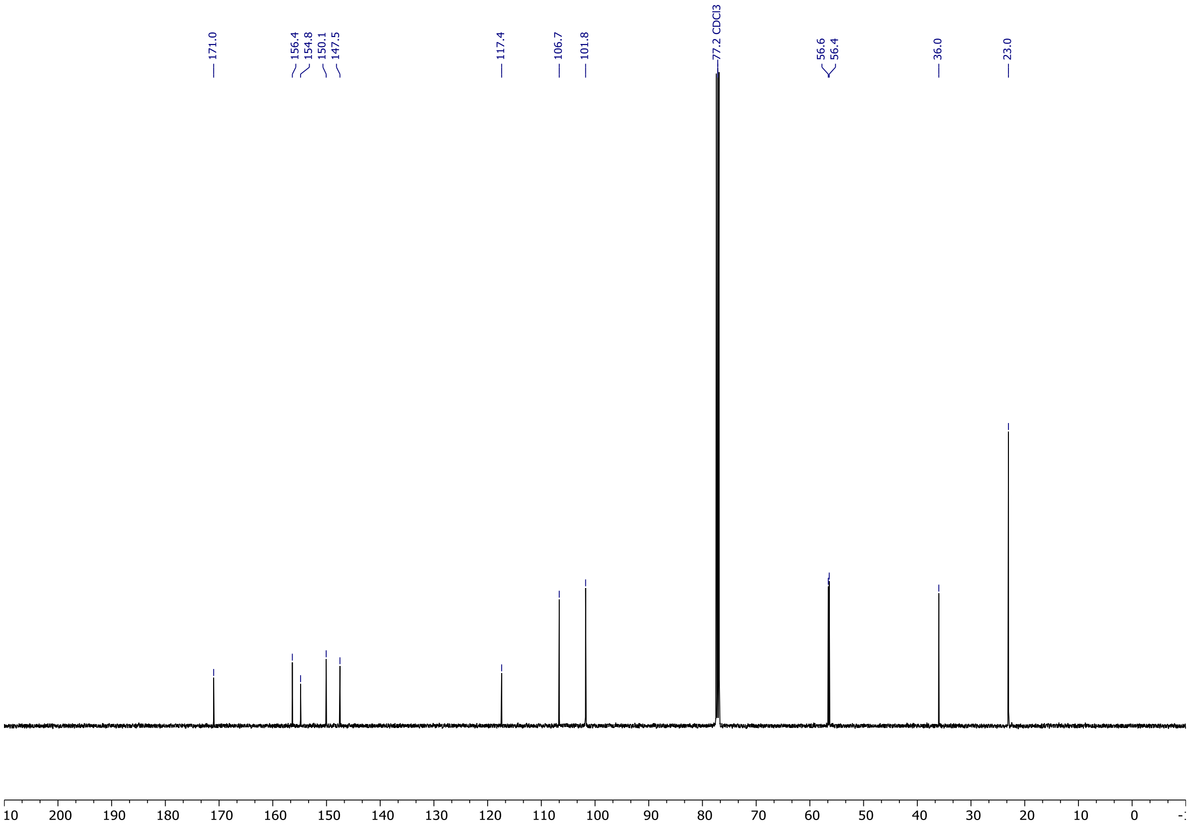

Figure S21: ^{13}C NMR (126 MHz, $CDCl_3$) spectrum.

4-Azido-6,7-dimethoxy-2-tosylquinazoline (**12a**)

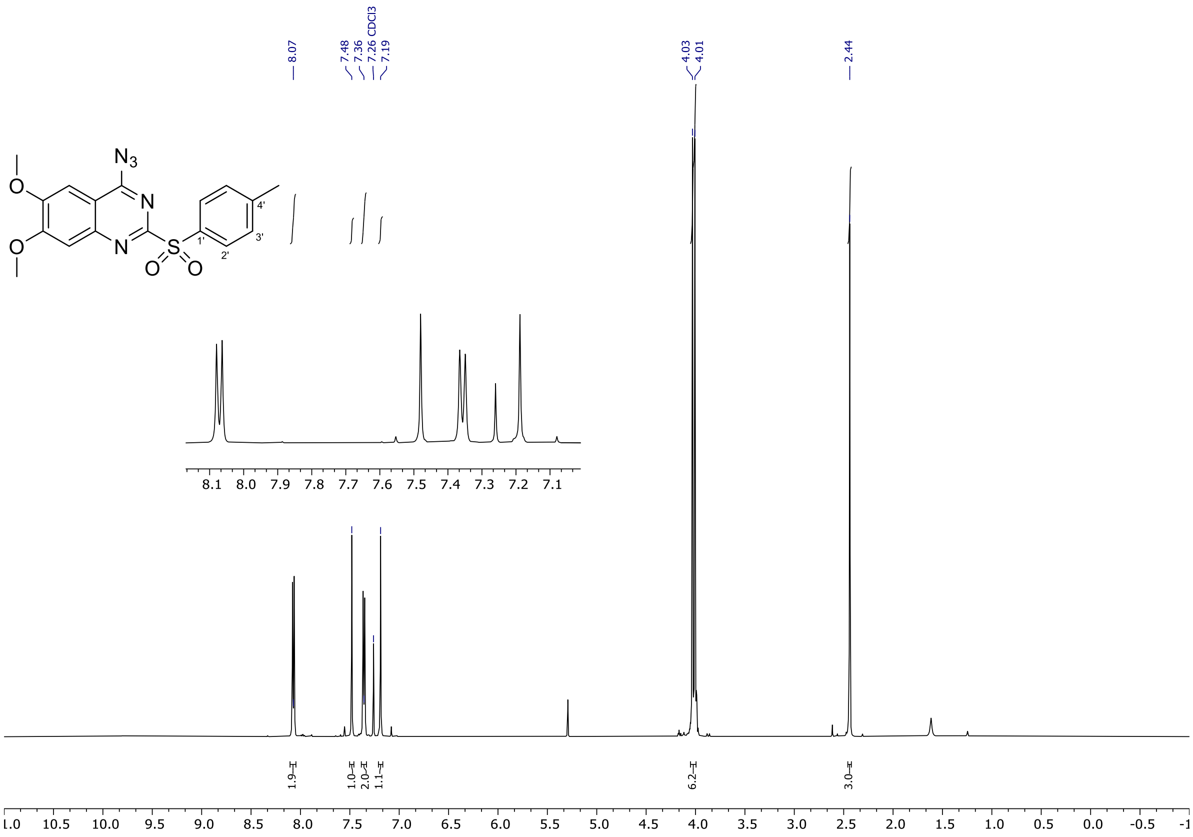

Figure S22: ^{1}H NMR (500 MHz, $CDCl_3$) spectrum.

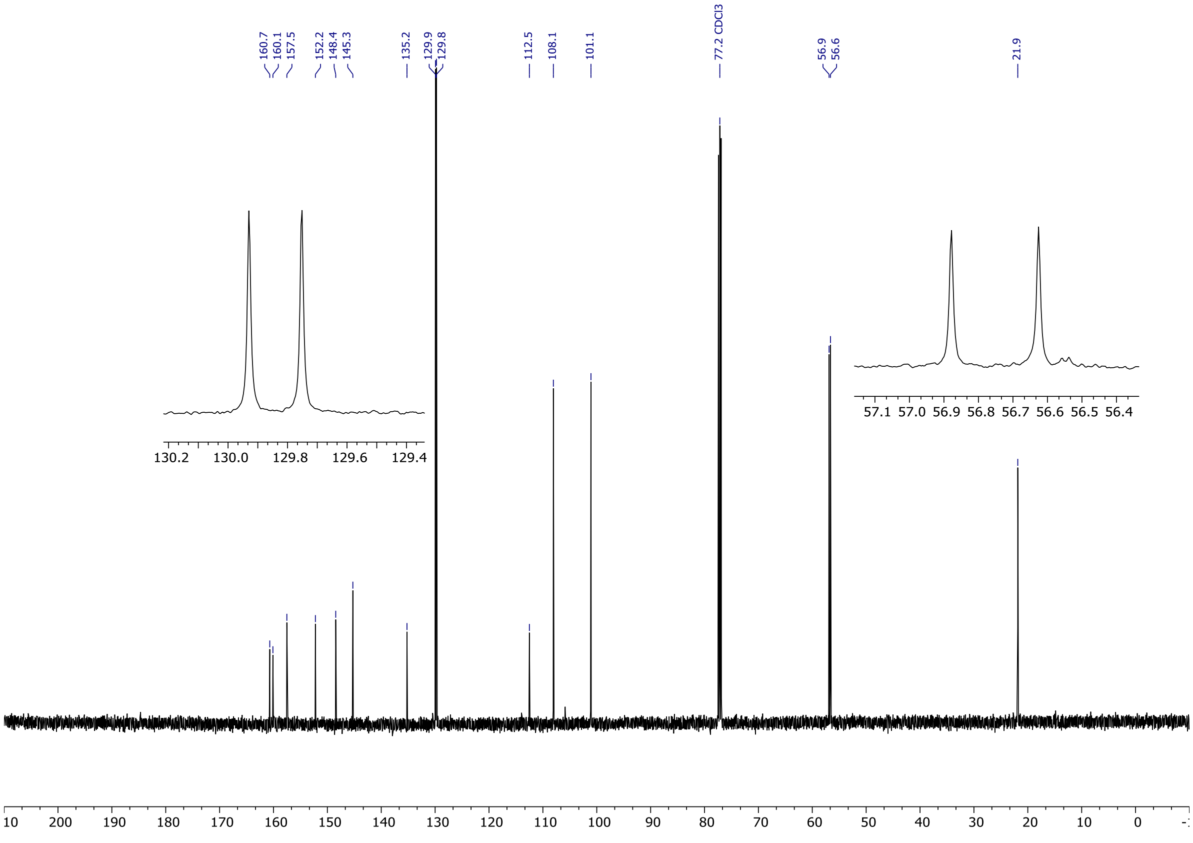

Figure S23: ^{13}C NMR (126 MHz, $CDCl_3$) spectrum.

4-Azido-2-(dodecylsulfonyl)-6,7-dimethoxyquinazoline (**12b**)

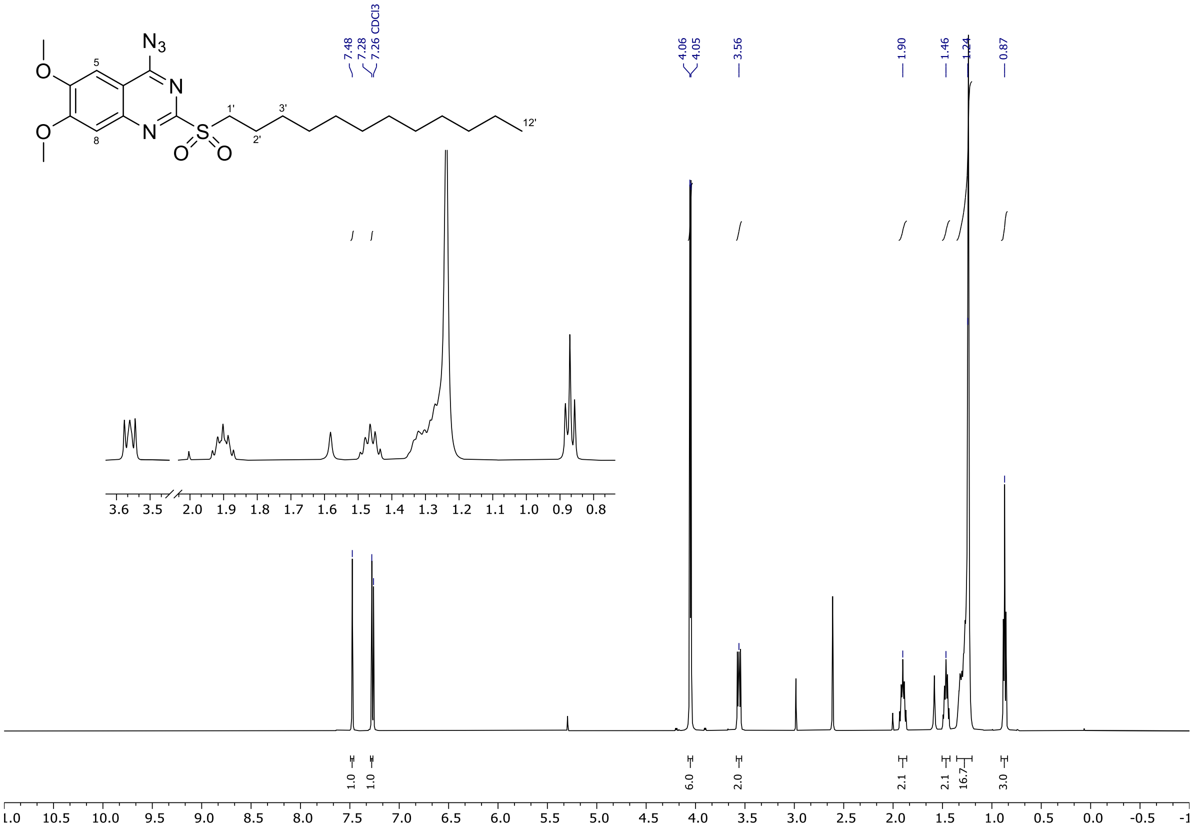

Figure S24: ^{1}H NMR (500 MHz, $CDCl_3$) spectrum.

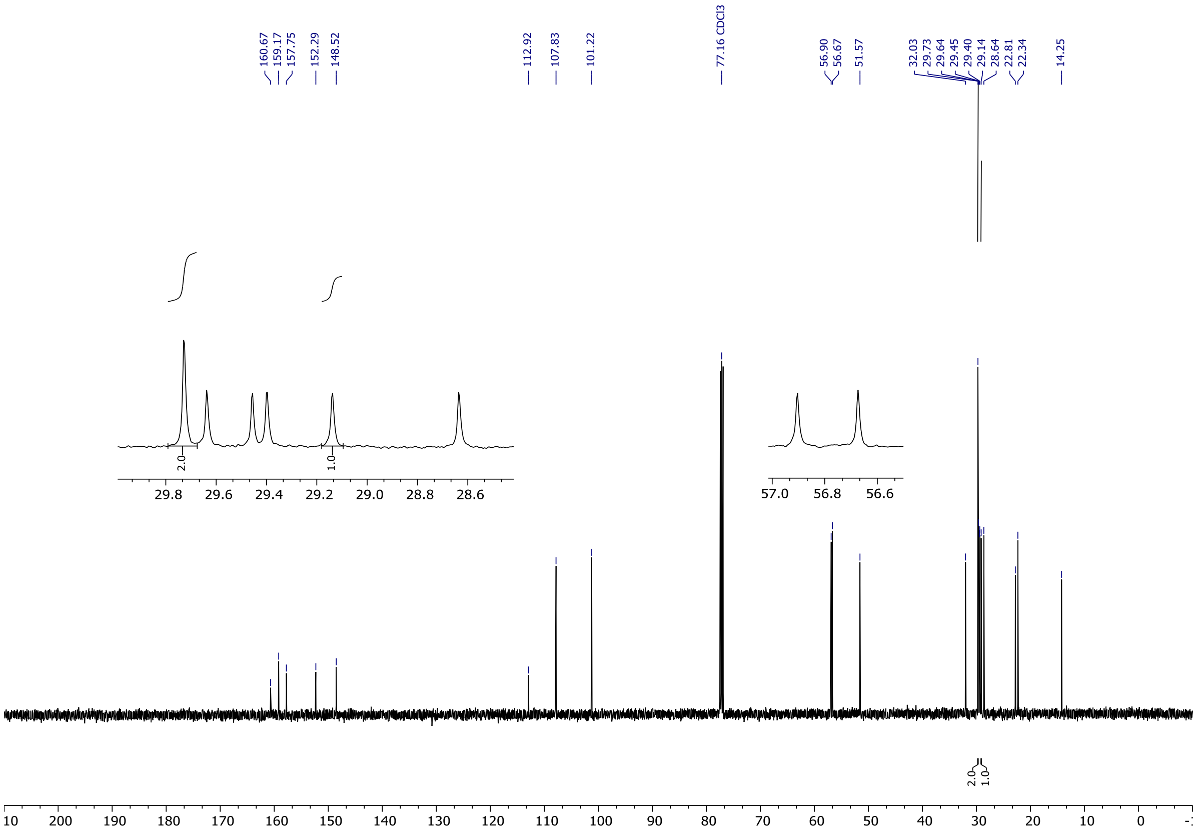

Figure S25: ^{13}C NMR (126 MHz, $CDCl_3$) spectrum.

4-Azido-6,7-dimethoxy-2-(phenylsulfonyl)quinazoline (**12c**)

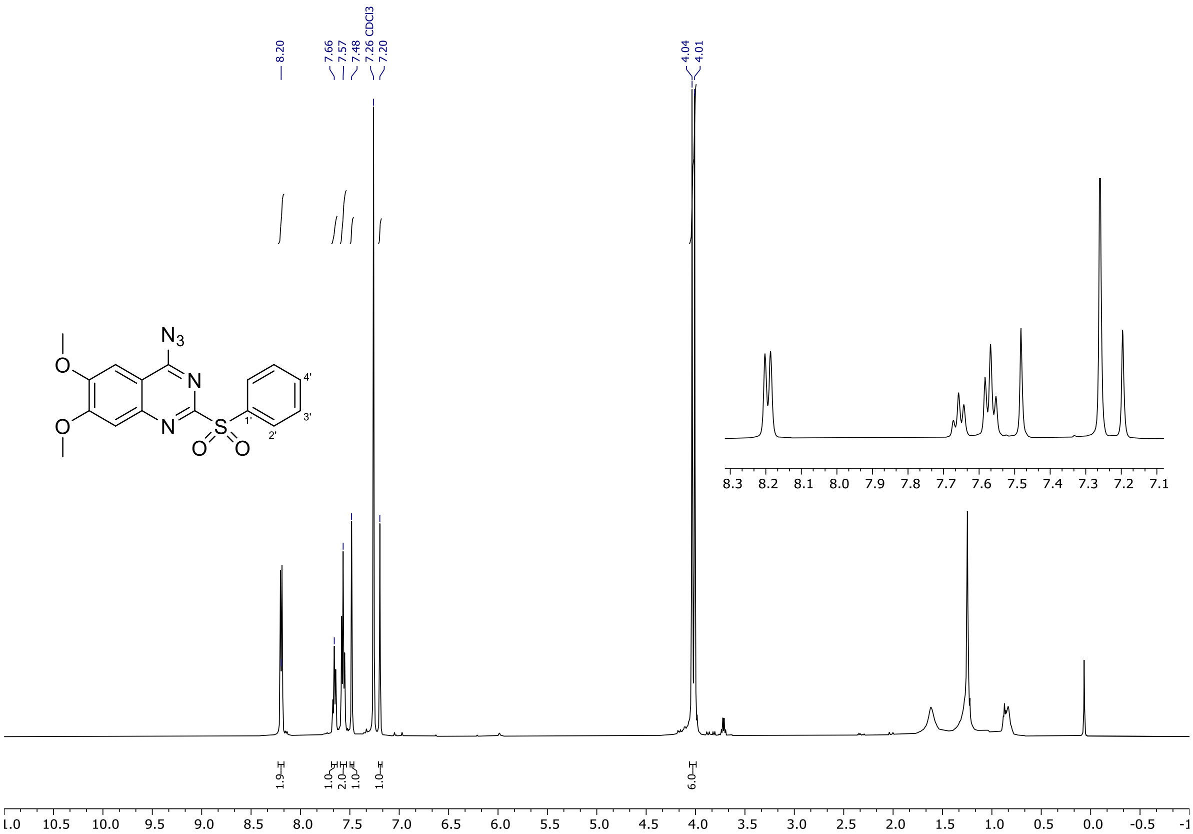

Figure S26: ^{1}H NMR (500 MHz, $CDCl_3$) spectrum.

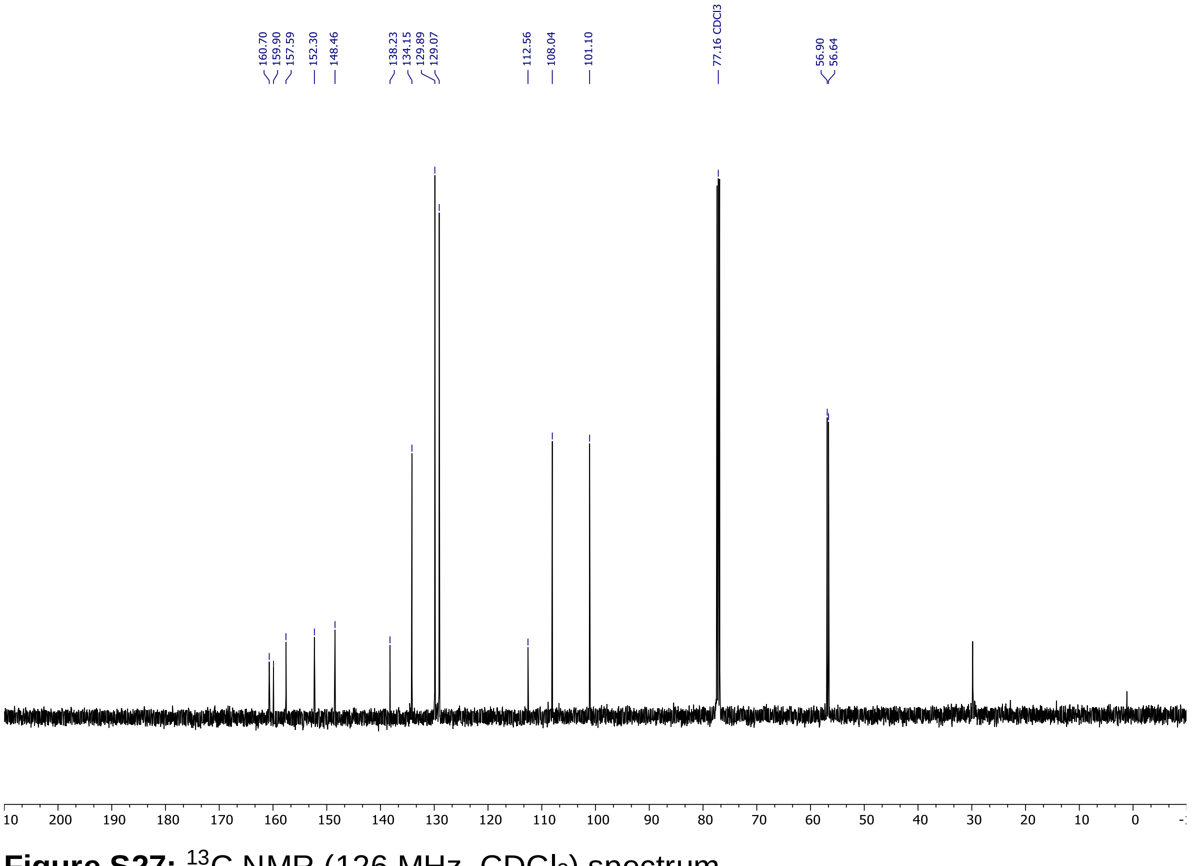

Figure S27: ^{13}C NMR (126 MHz, $CDCl_3$) spectrum.

4-Azido-2-(isopropylsulfonyl)-6,7-dimethoxyquinazoline (**12d**)

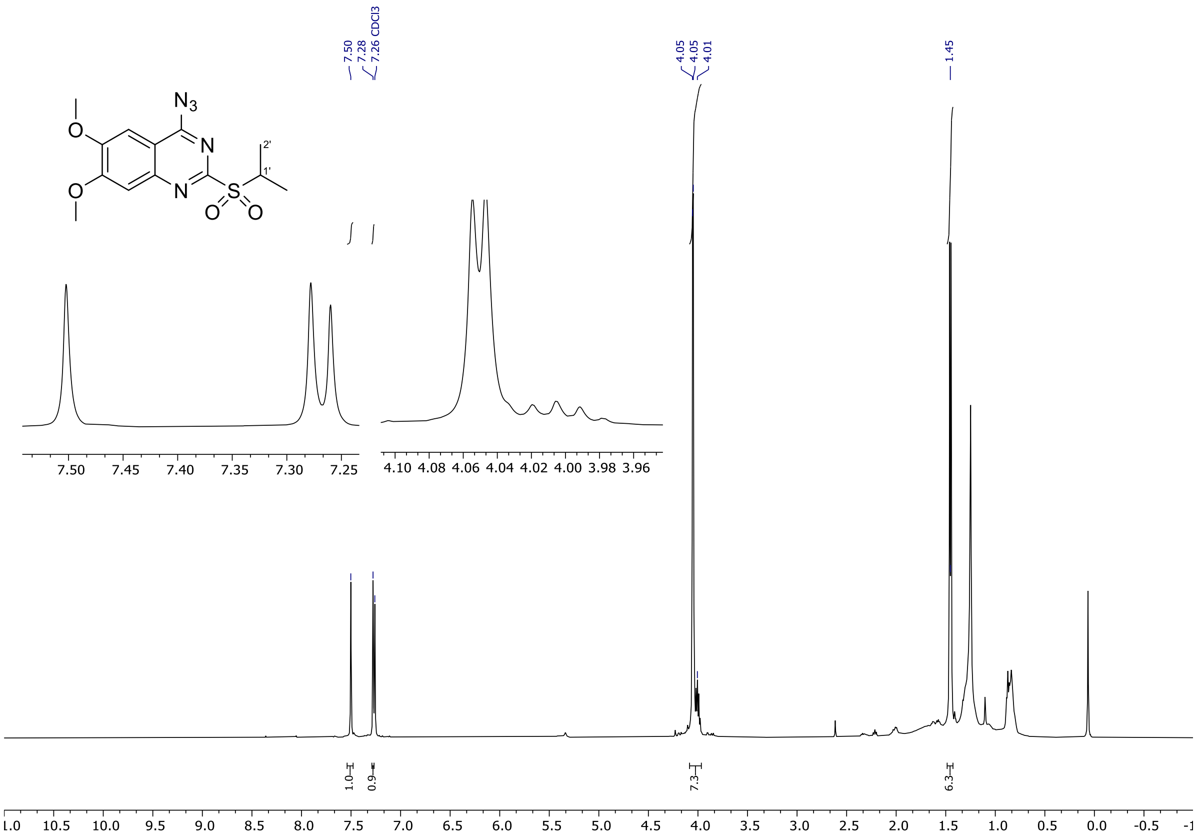

Figure S28: ^{1}H NMR (500 MHz, $CDCl_3$) spectrum.

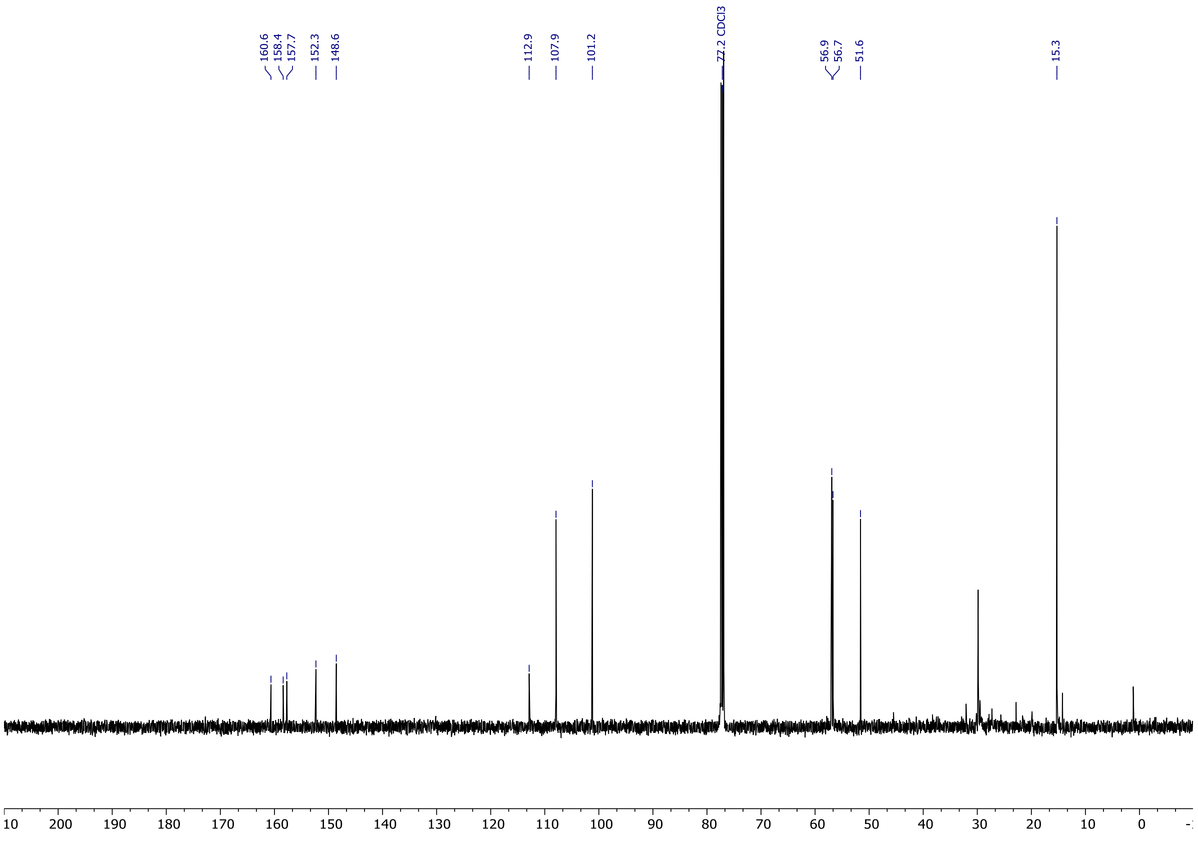

Figure S29: ^{13}C NMR (126 MHz, $CDCl_3$) spectrum.

2,4-Diazido-6,7-dimethoxyquinazoline (**13**)

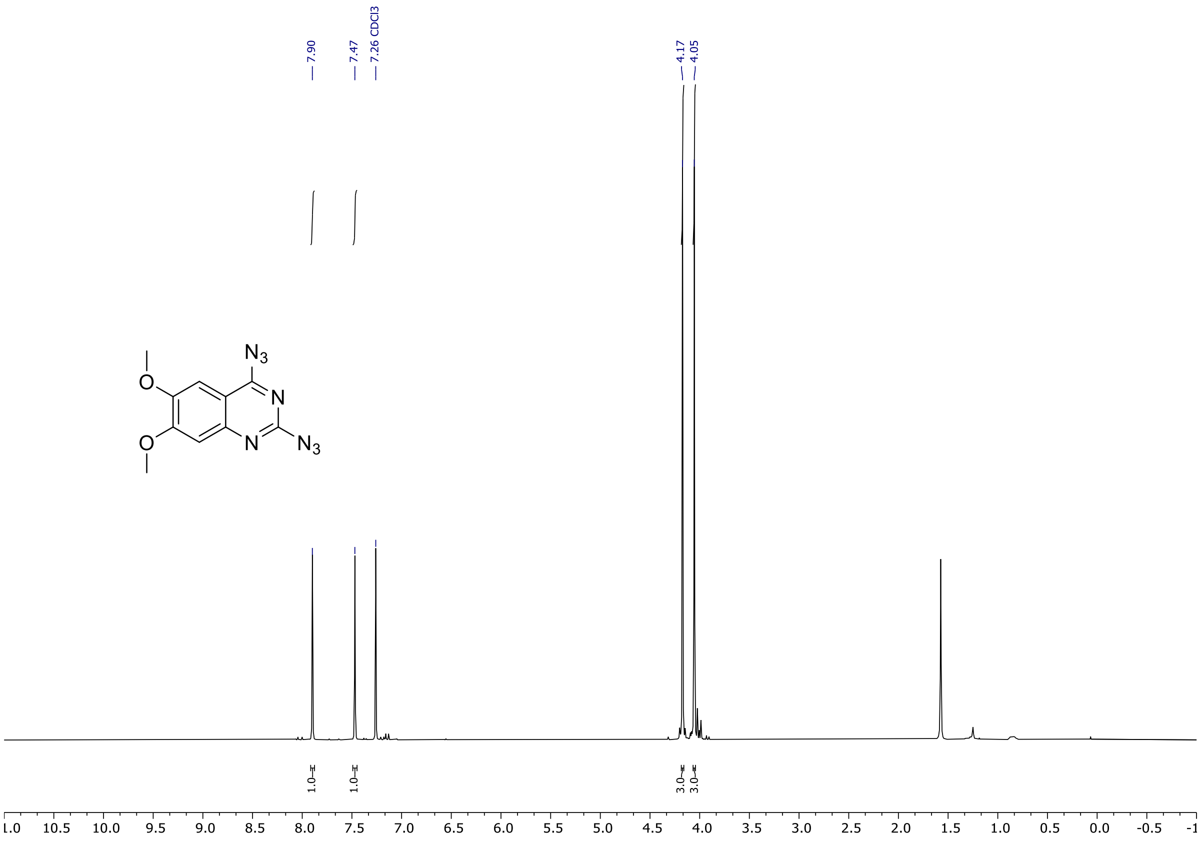

Figure S30: ^{1}H NMR (500 MHz, $CDCl_3$) spectrum.

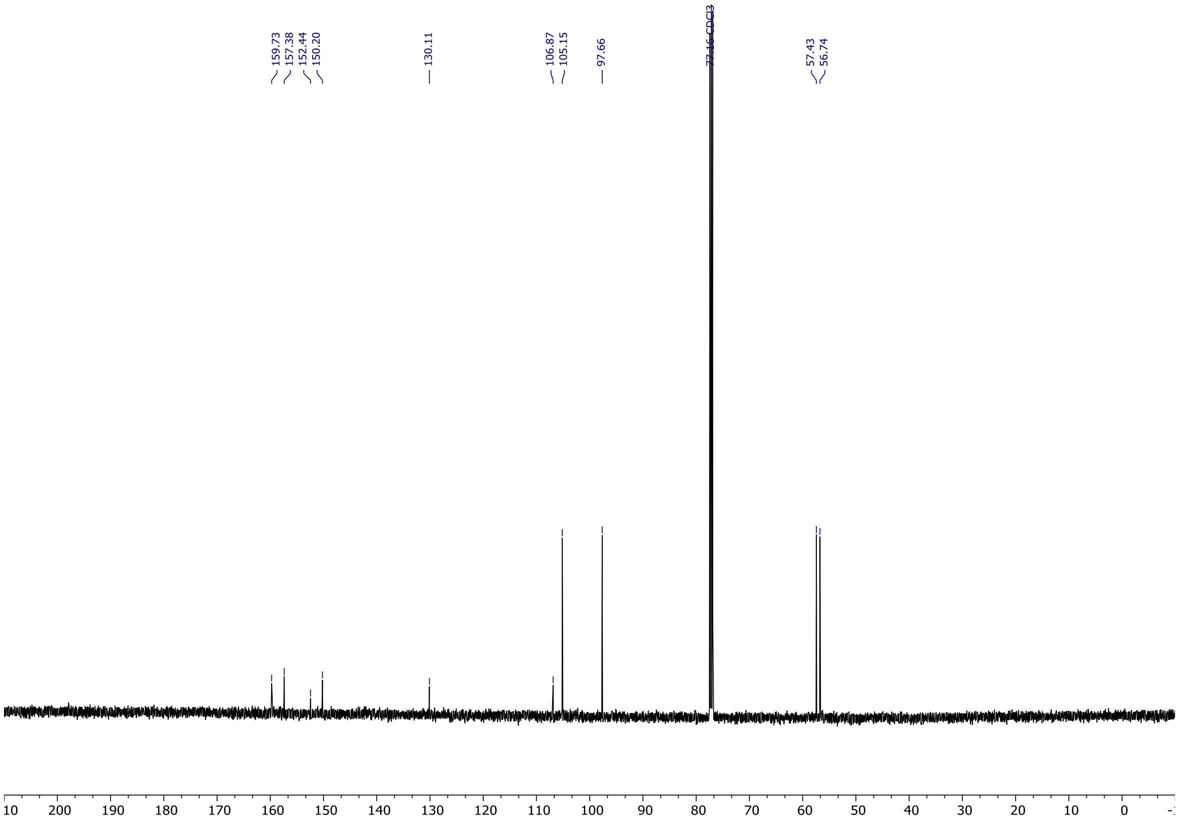

Figure S31: ^{13}C NMR (126 MHz, $CDCl_3$) spectrum.

2-Azido-6,7-dimethoxy-4-(*p*-tolylthio)quinazoline (**14a**)

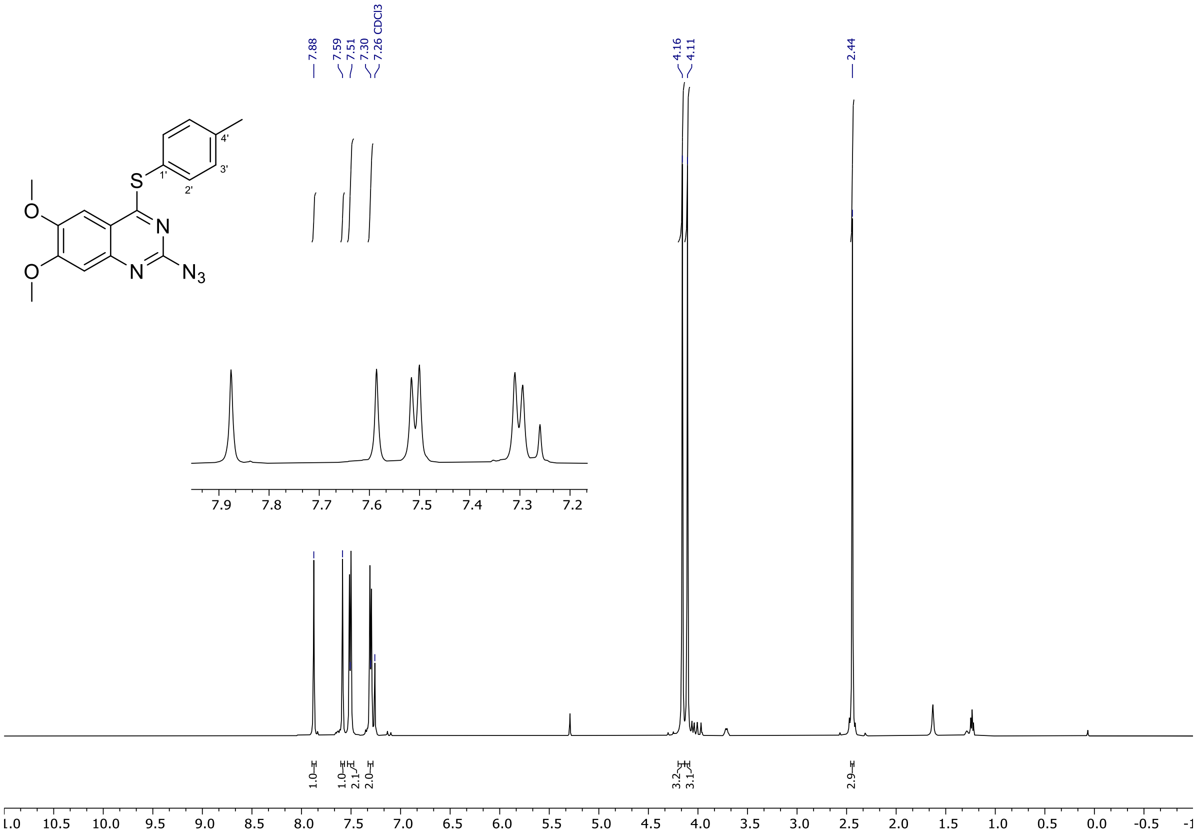

Figure S32: ^{1}H NMR (500 MHz, $CDCl_3$) spectrum.

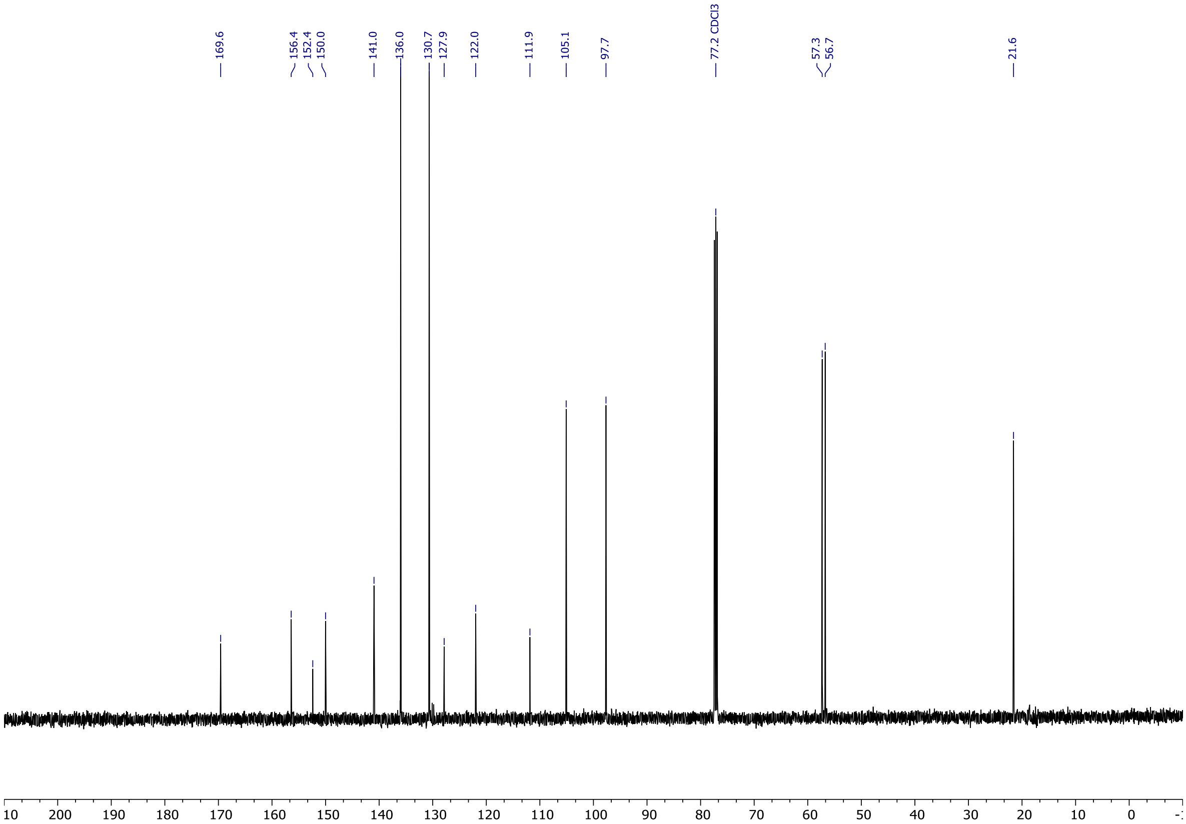

Figure S33: ^{13}C NMR (126 MHz, $CDCl_3$) spectrum.

2-Azido-4-(dodecylthio)-6,7-dimethoxyquinazoline (**14b**)

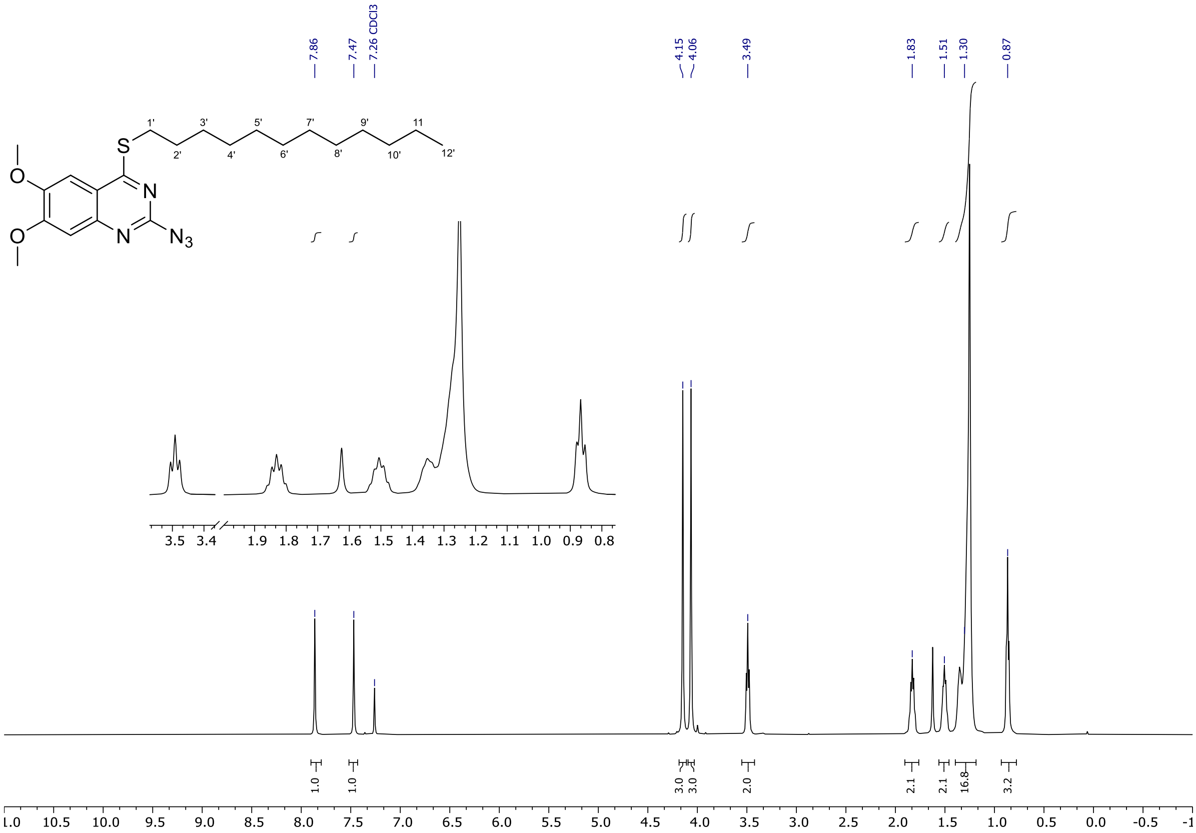

Figure S34: ^{1}H NMR (500 MHz, $CDCl_3$) spectrum.

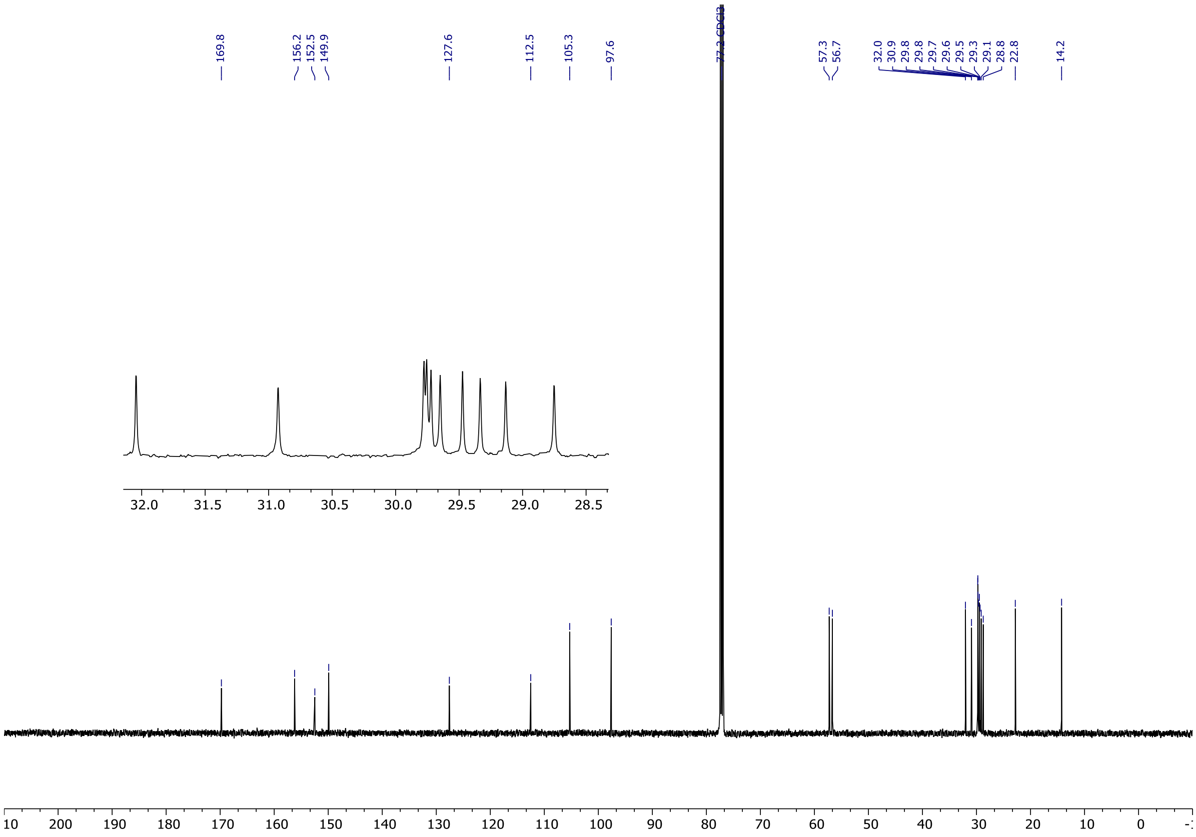

Figure S35: ^{13}C NMR (126 MHz, $CDCl_3$) spectrum.

2-Azido-6,7-dimethoxy-4-(phenylthio)quinazoline (**14c**)

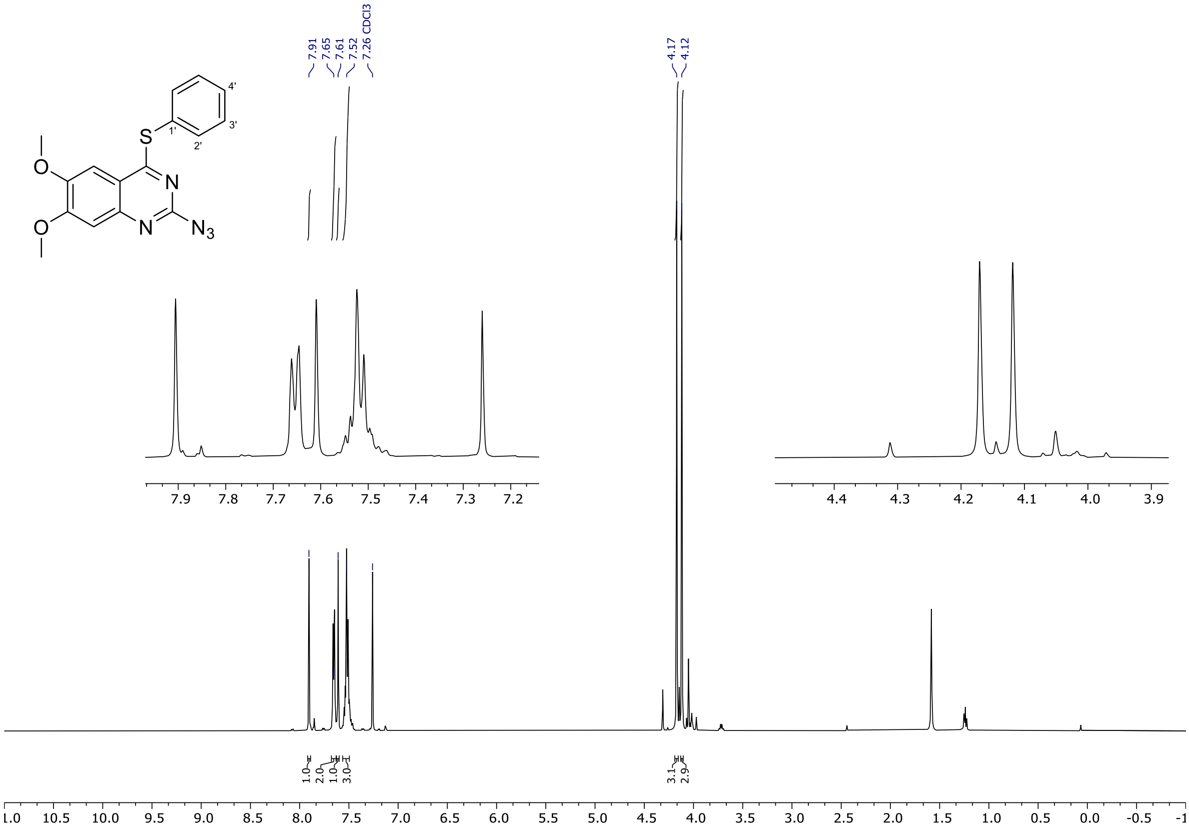

Figure S36: ^{1}H NMR (500 MHz, $CDCl_3$) spectrum.

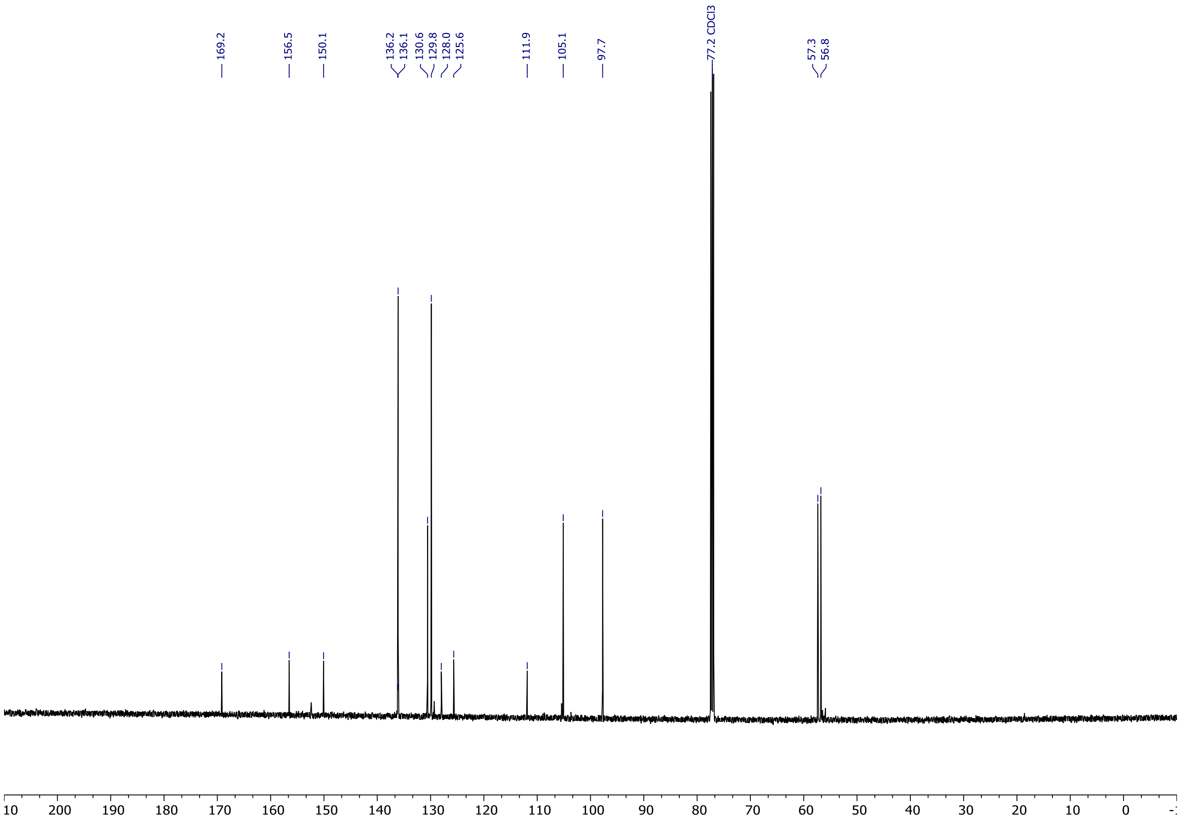

Figure S37: ^{13}C NMR (126 MHz, $CDCl_3$) spectrum.

2-Azido-4-(isopropylthio)-6,7-dimethoxyquinazoline (**14d**)

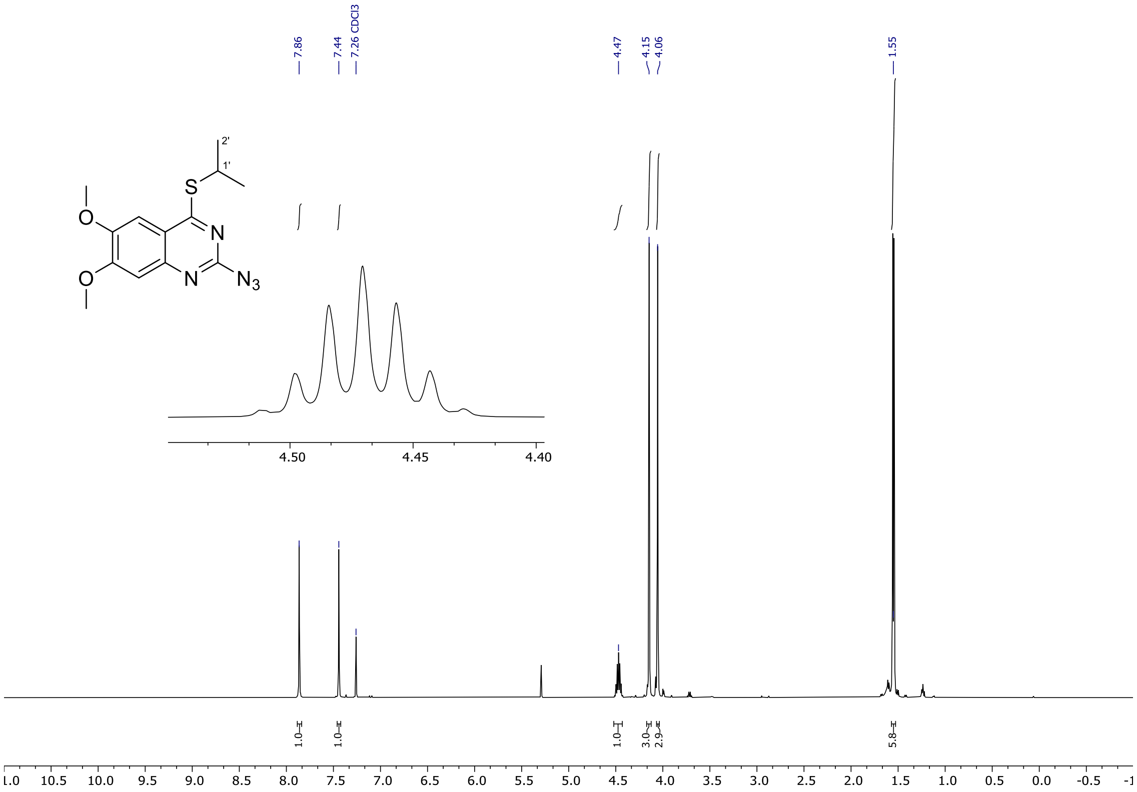

Figure S38: ^{1}H NMR (500 MHz, $CDCl_3$) spectrum.

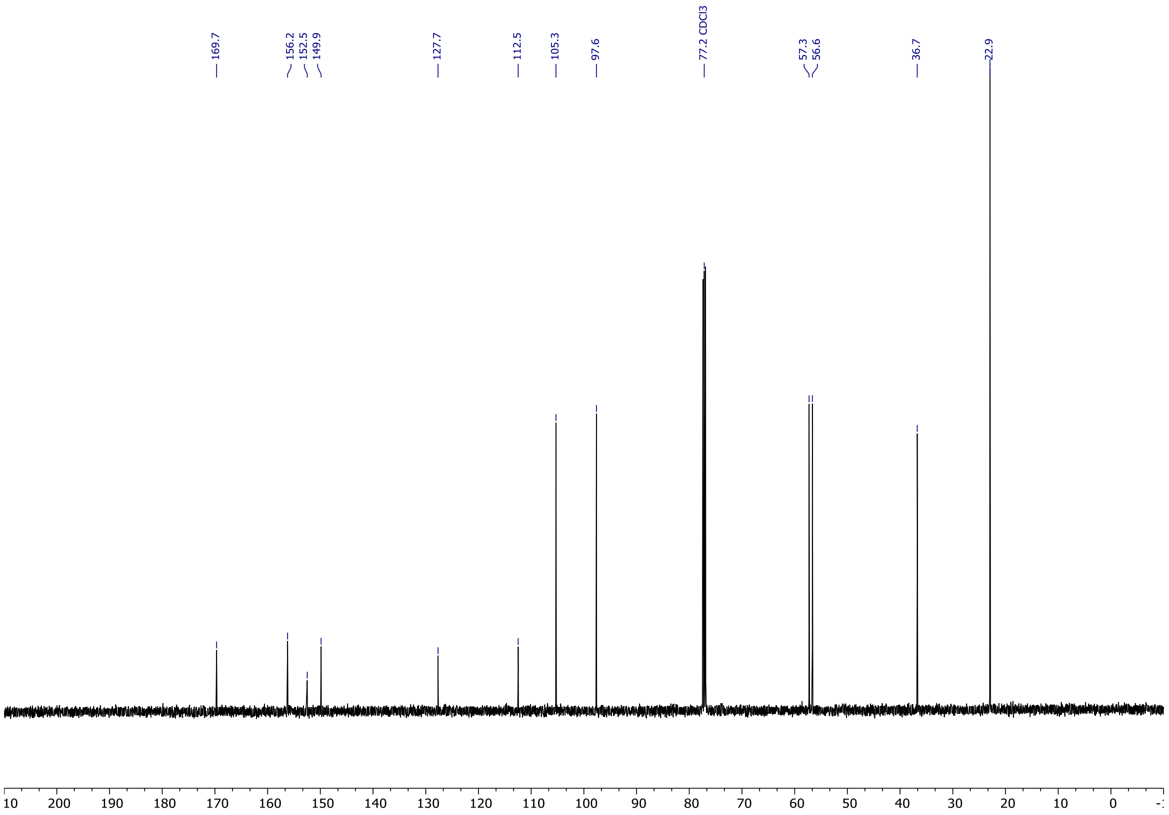

Figure S39: ^{13}C NMR (126 MHz, $CDCl_3$) spectrum.

Tautomeric mixture of 2-azido-6,7-dimethoxy-4-tosylquinazoline and 7,8-dimethoxy-5-tosyltetrazolo[1,5-*a*]quinazoline (**15a**)

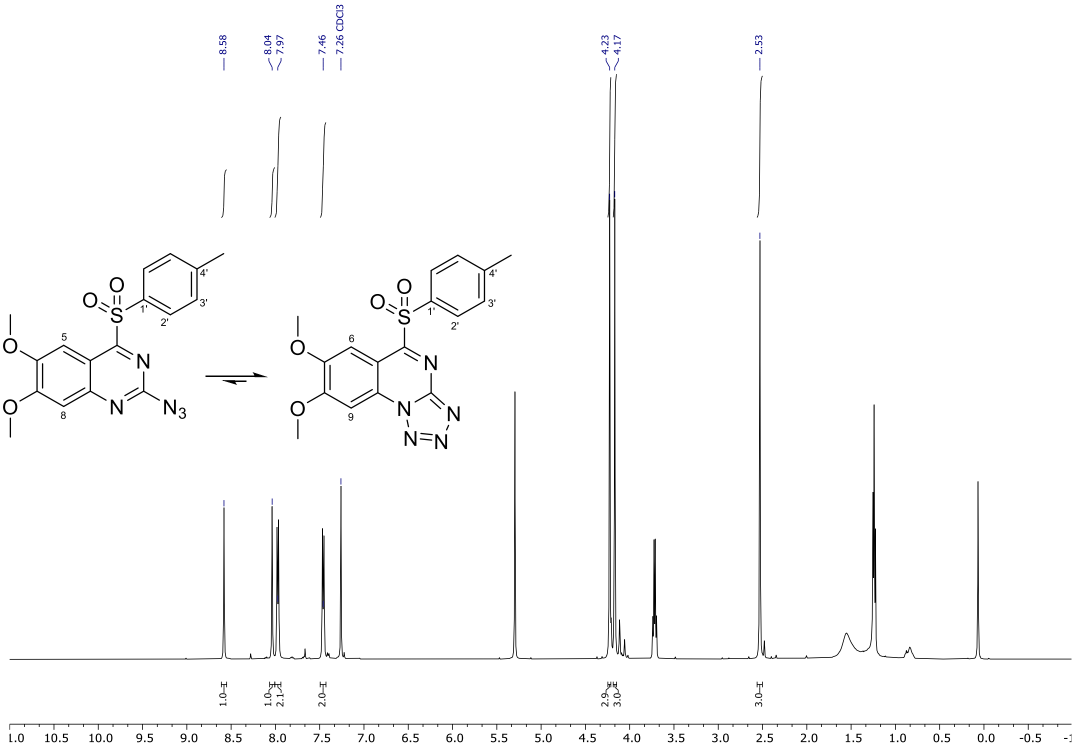

Figure S40: ^{1}H NMR (500 MHz, $CDCl_3$) spectrum.

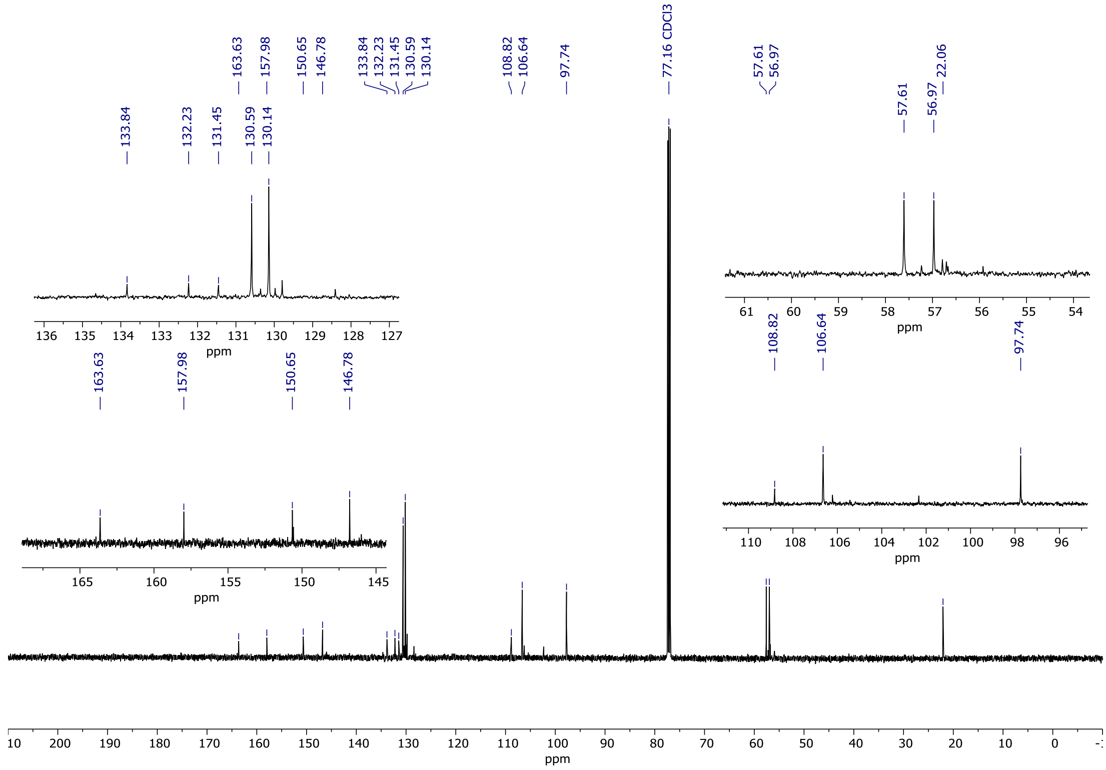

Figure S41: ^{13}C NMR (126 MHz, $CDCl_3$) spectrum.

Tautomeric mixture of 2-azido-4-(dodecylsulfonyl)-6,7-dimethoxyquinazoline and 5-(dodecylsulfonyl)-7,8-dimethoxytetrazolo[1,5-*a*]quinazoline (**15b**)

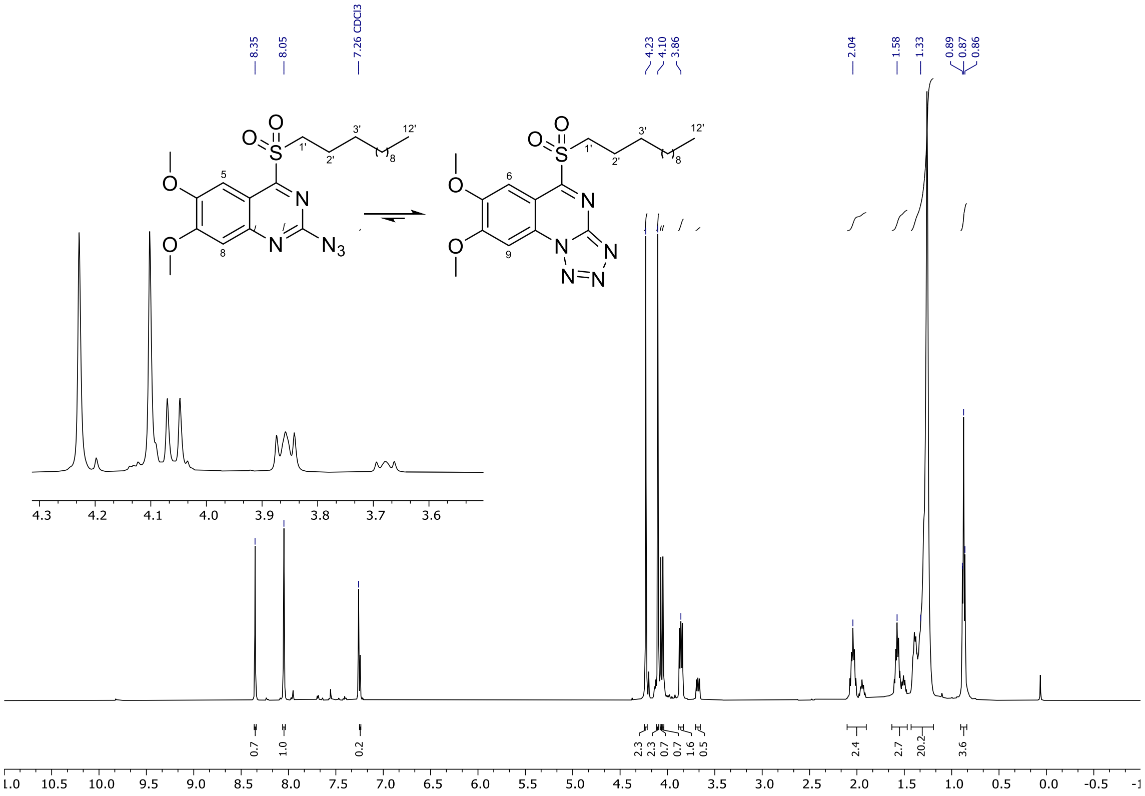

Figure S42: ^{1}H NMR (500 MHz, $CDCl_3$) spectrum.

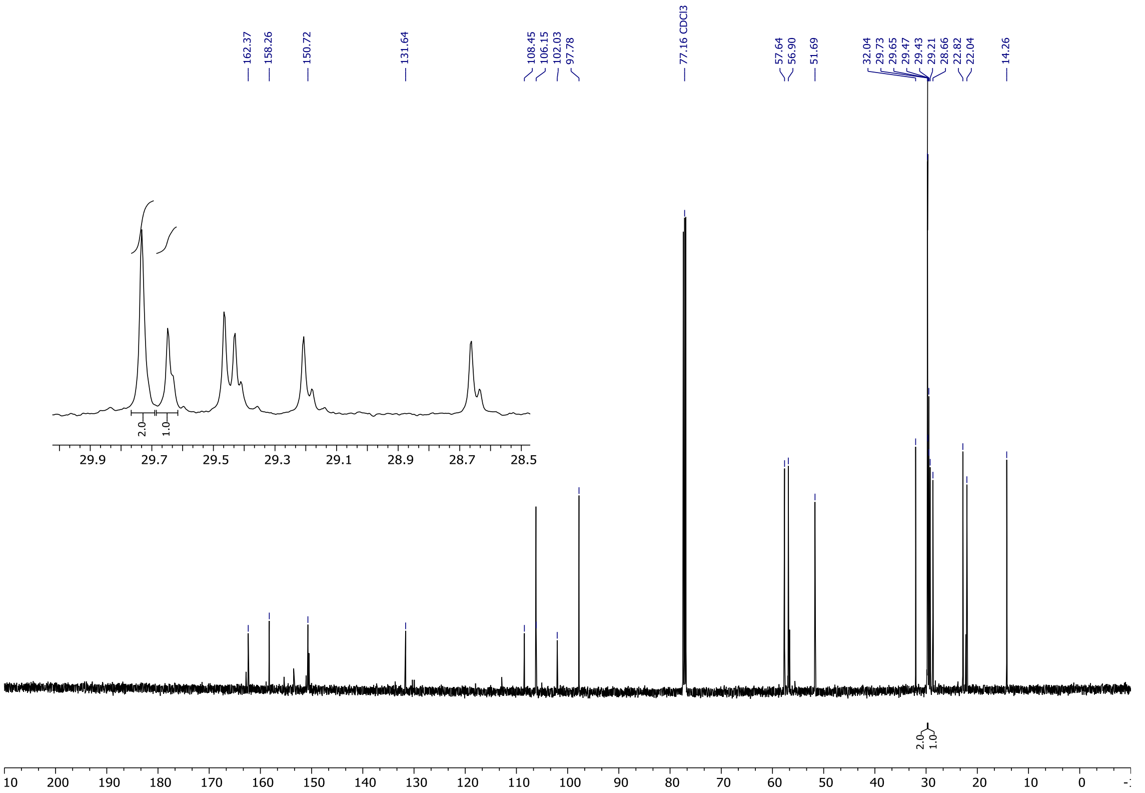

Figure S43: ^{13}C NMR (126 MHz, $CDCl_3$) spectrum.

Tautomeric mixture of 2-azido-6,7-dimethoxy-4-(phenylsulfonyl)quinazoline and 7,8-dimethoxy-5-(phenylsulfonyl)tetrazolo[1,5-*a*]quinazoline (**15c**)

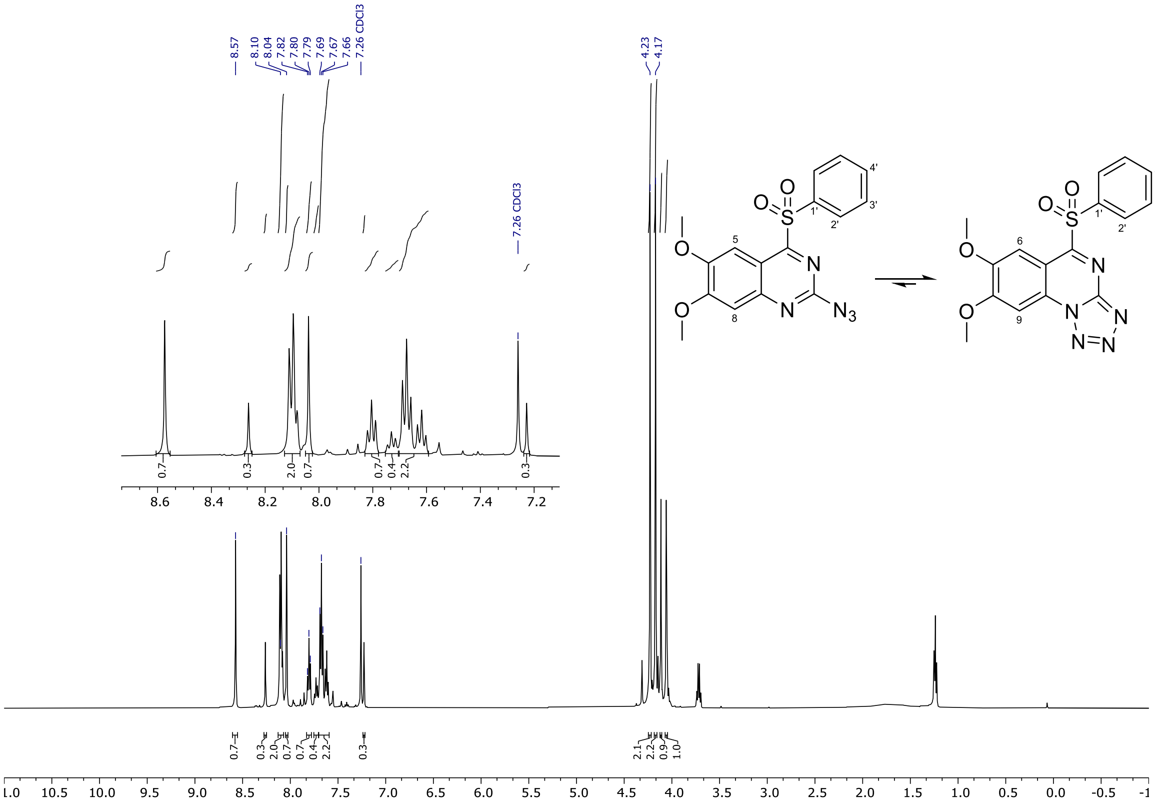

Figure S44: ^{1}H NMR (500 MHz, $CDCl_3$) spectrum.

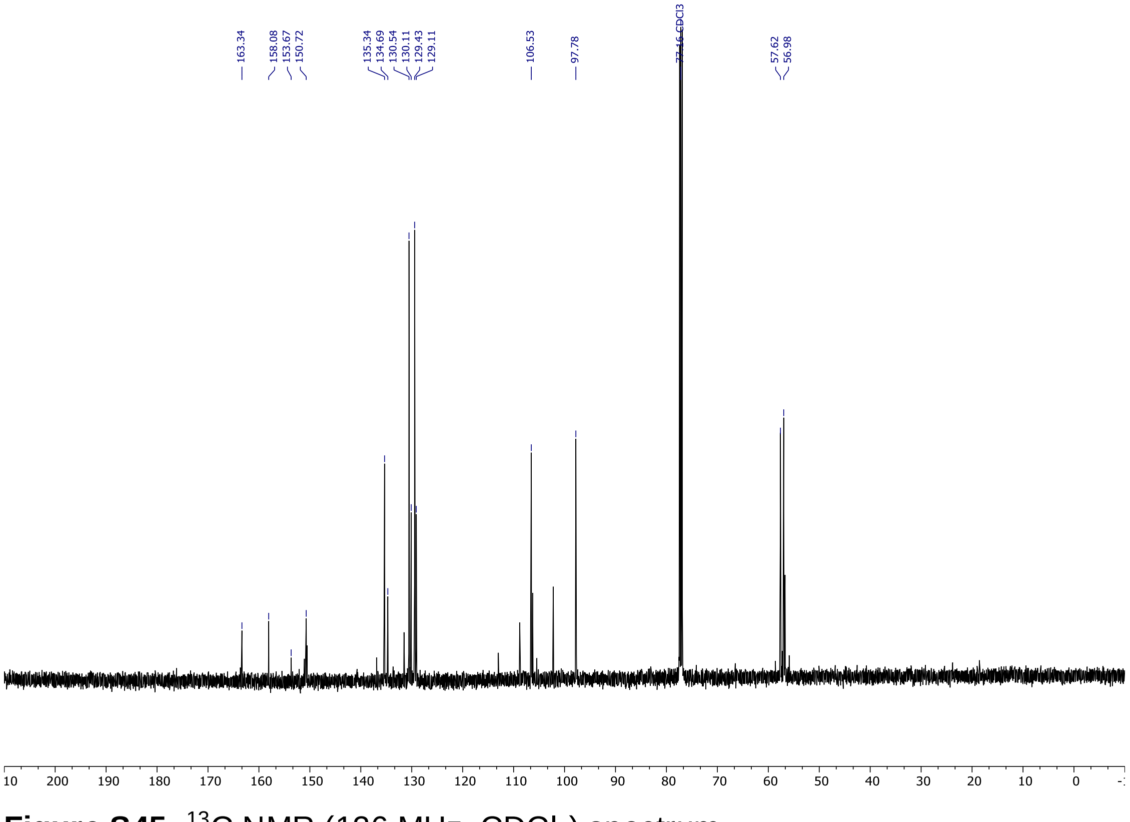

Figure S45: ^{13}C NMR (126 MHz, $CDCl_3$) spectrum.

Tautomeric mixture of 2-azido-4-(isopropylsulfonyl)-6,7-dimethoxyquinazoline and 5-(isopropylsulfonyl)-7,8-dimethoxytetrazolo[1,5-*a*]quinazoline (**15d**)

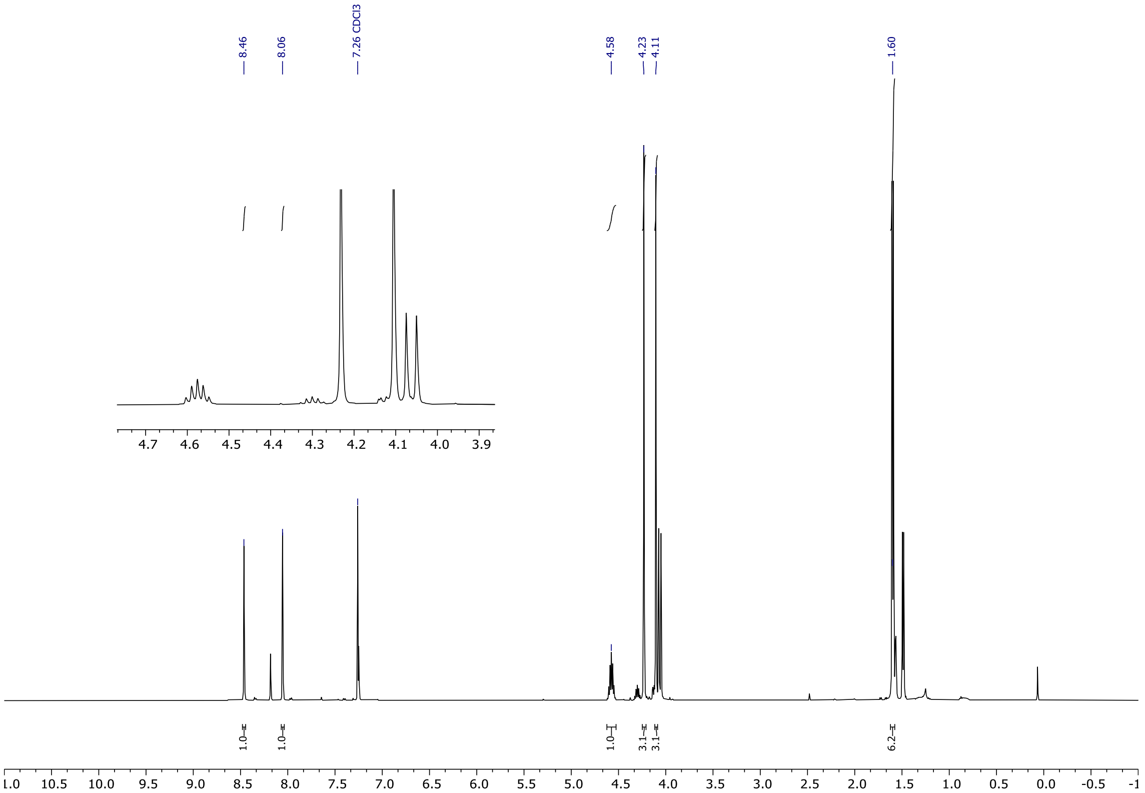

Figure S46: ^{1}H NMR (500 MHz, $CDCl_3$) spectrum.

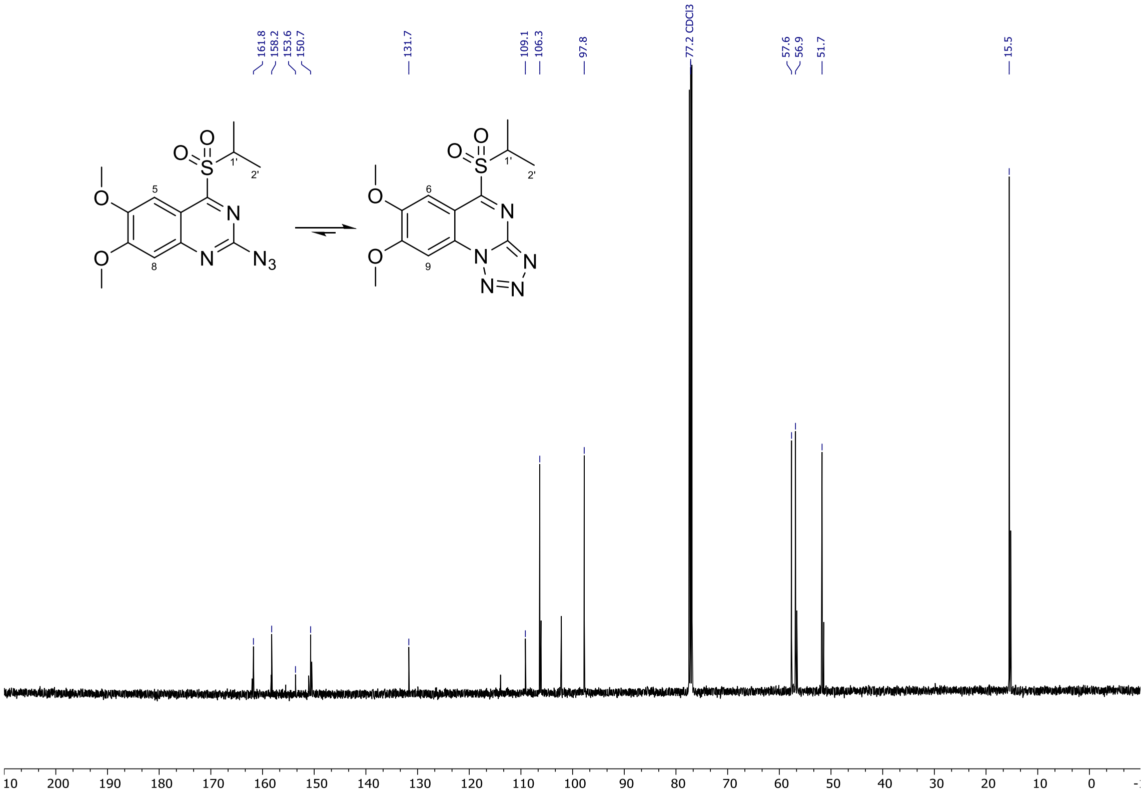

Figure S47: ^{13}C NMR (126 MHz, $CDCl_3$) spectrum.

2-Azido-6,7-dimethoxy-4-(pyrrolidin-1-yl)quinazoline (**16**)

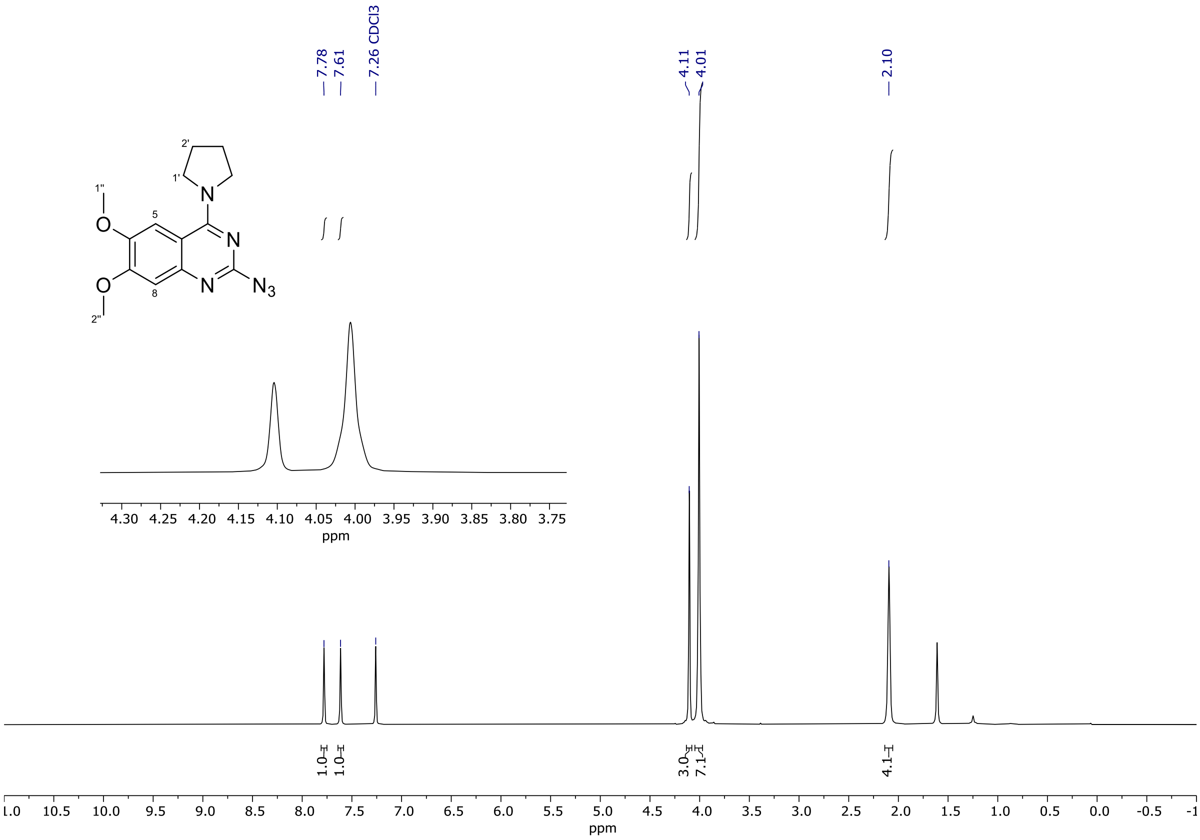

Figure S48: ^{1}H NMR (500 MHz, CDCl$_3$) spectrum.

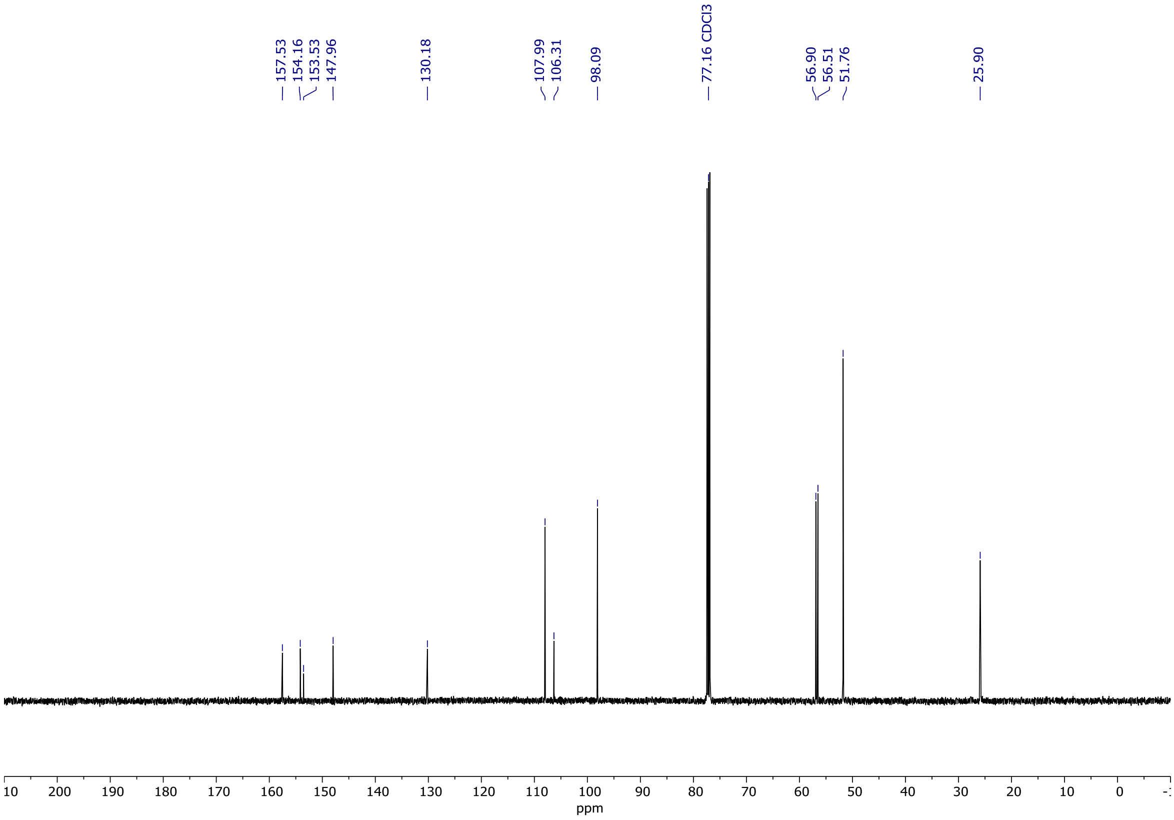

Figure S49: ^{13}C NMR (126 MHz, CDCl$_3$) spectrum.

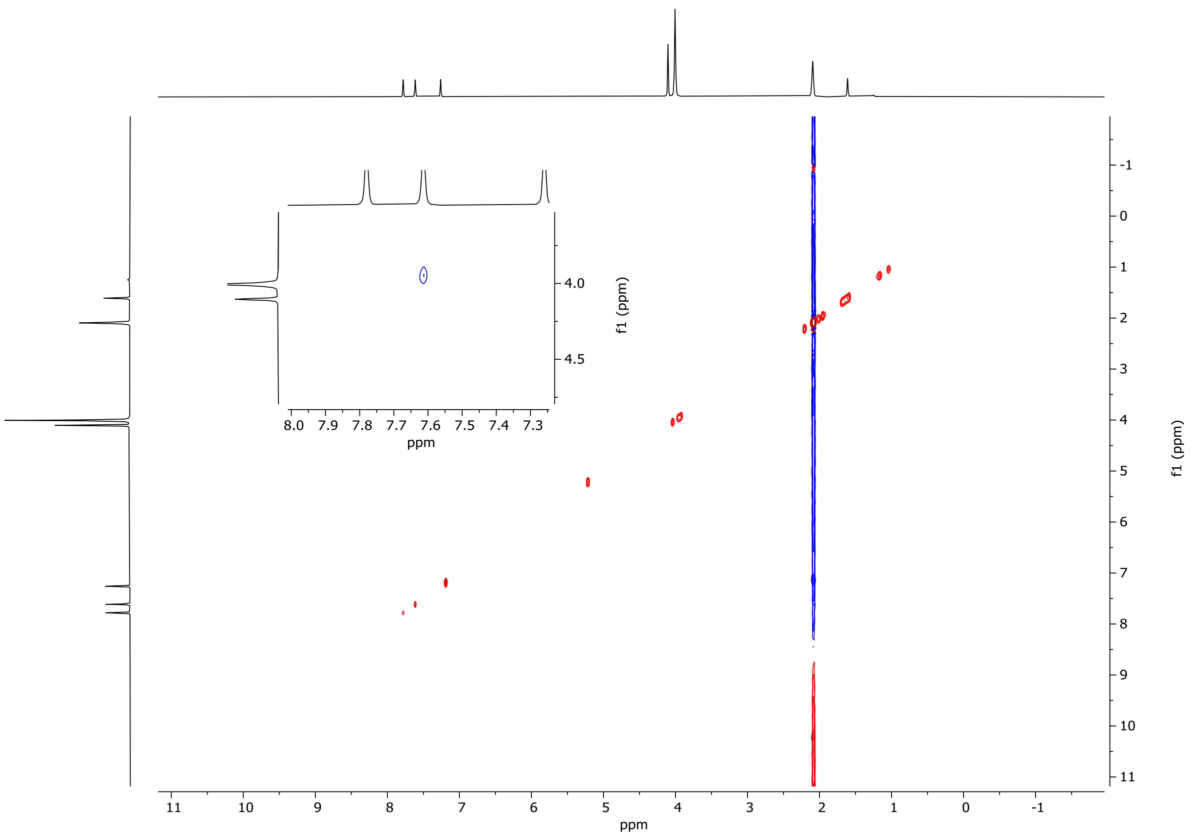

Figure S50: ^{1}H-^{1}H NOESY spectrum.

6,7-Dimethoxy-4-(pyrrolidin-1-yl)-2-tosylquinazoline (**18**)

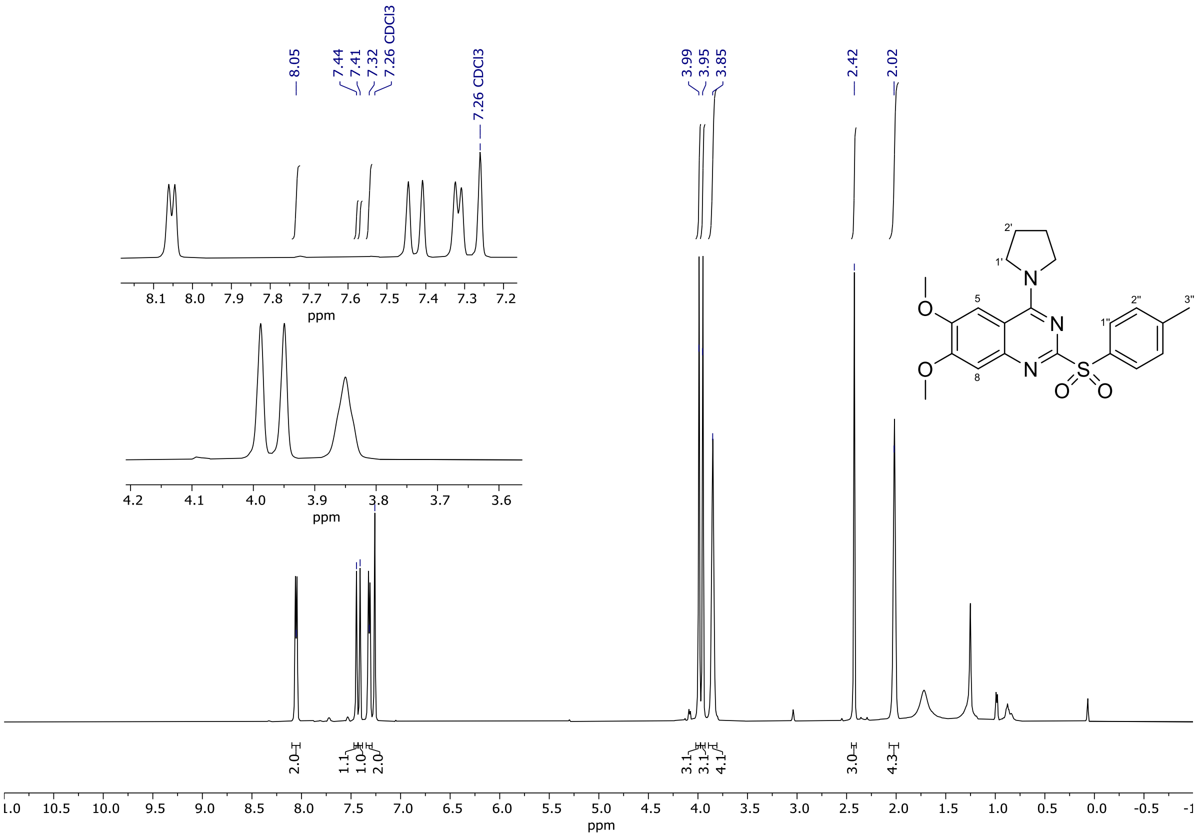

Figure S51: ^{1}H NMR (500 MHz, $CDCl_3$) spectrum.

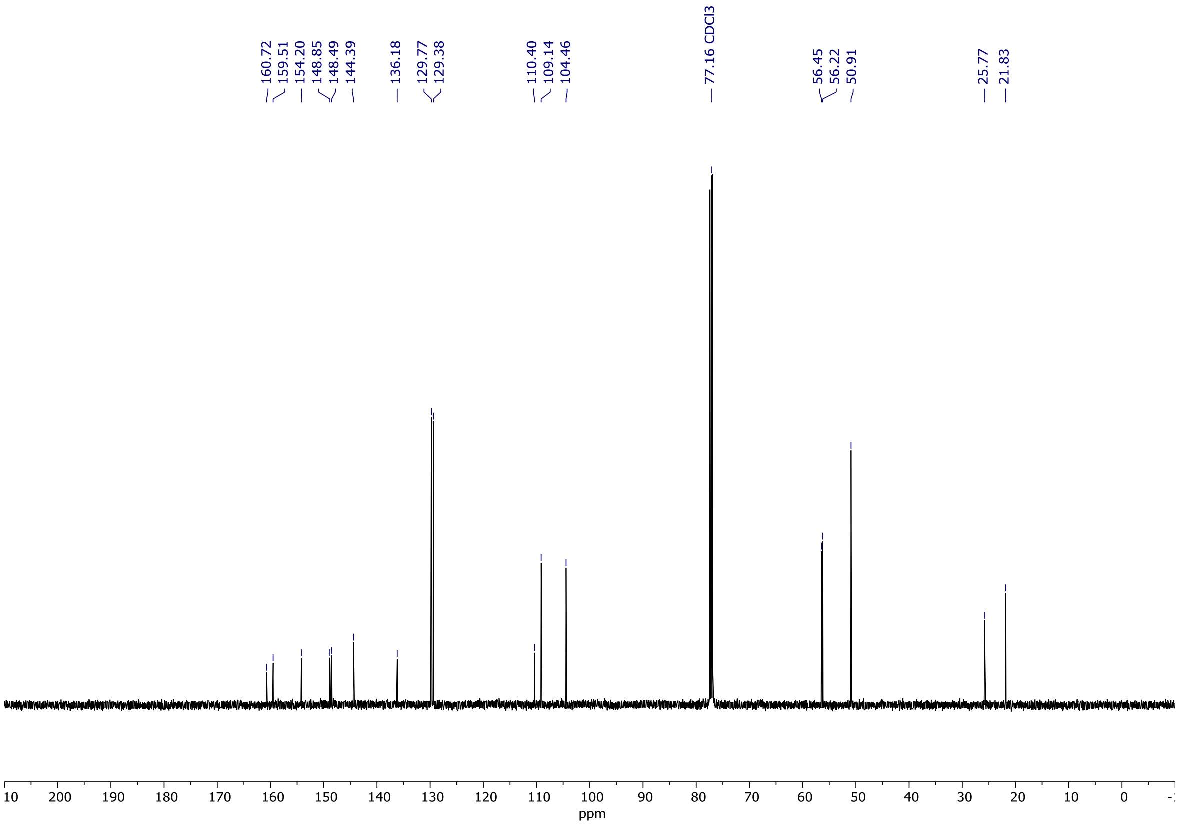

Figure S52: ^{13}C NMR (126 MHz, $CDCl_3$) spectrum.

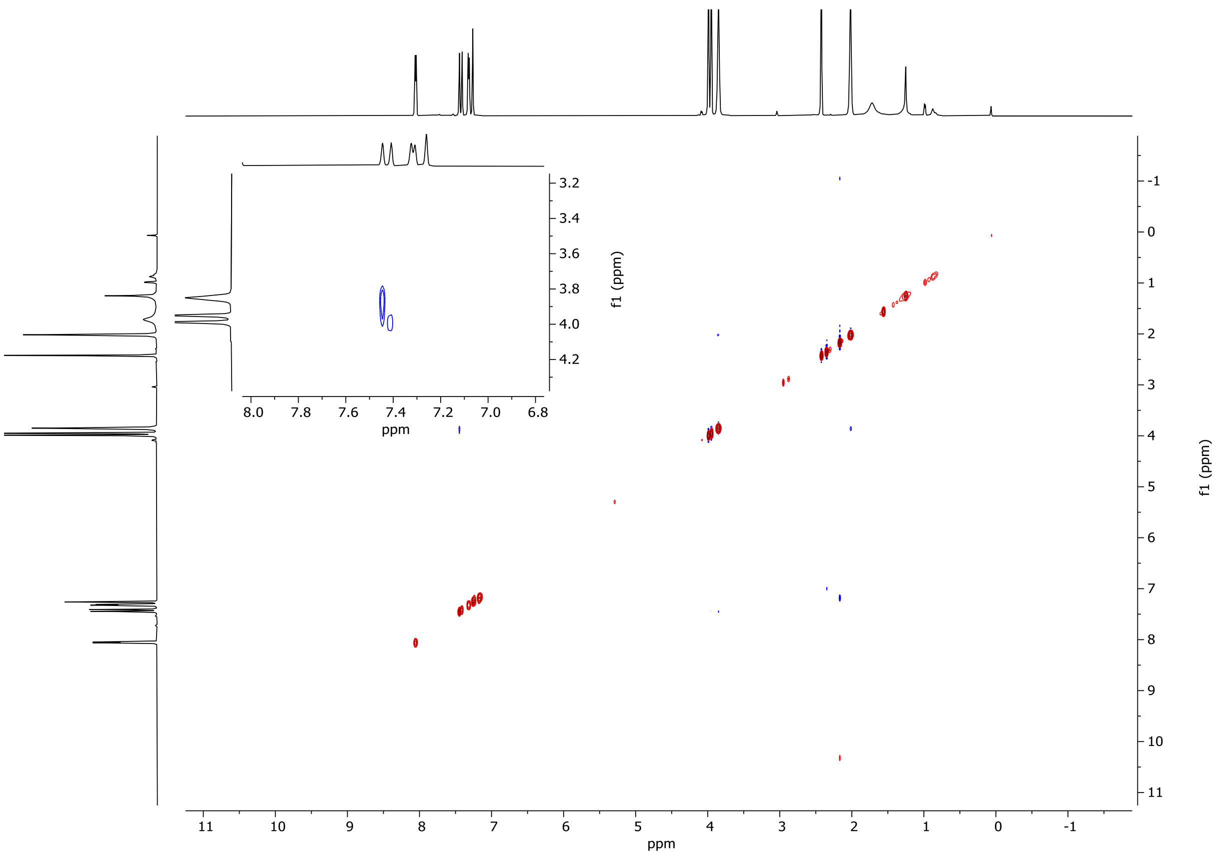

Figure S53: ^{1}H-^{1}H NOESY spectrum.

Tautomeric mixture of 4-azido-6,7-dimethoxy-2-(pyrrolidin-1-yl)quinazoline and 8,9-dimethoxy-5-(pyrrolidin-1-yl)tetrazolo[1,5-*c*]quinazoline (**17a**)

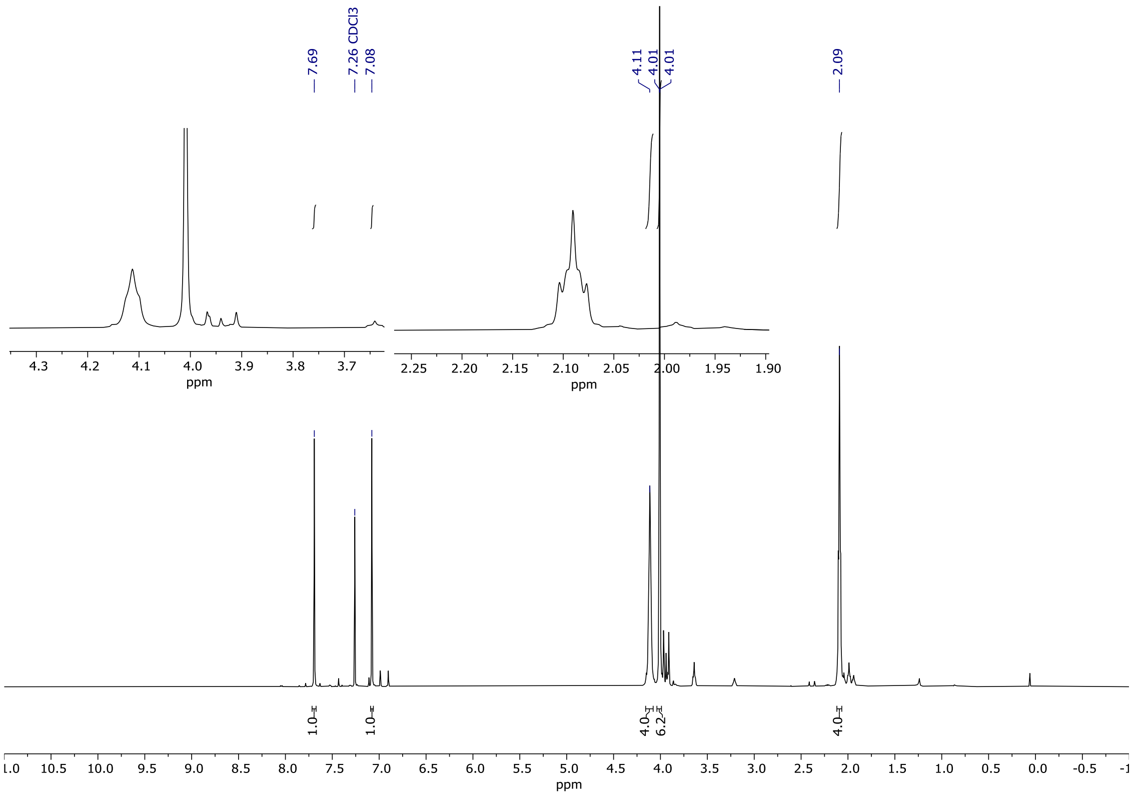

Figure S54: ^{1}H NMR (500 MHz, $CDCl_3$) spectrum.

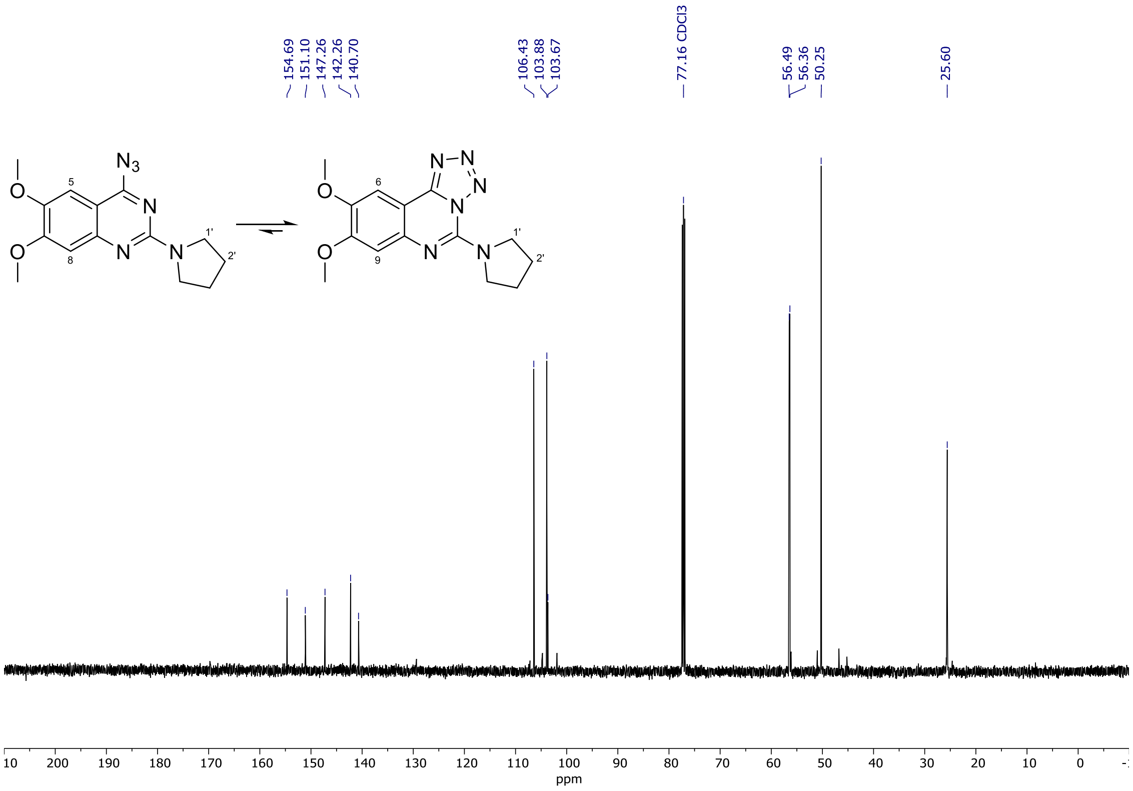

Figure S55: ^{13}C NMR (126 MHz, $CDCl_3$) spectrum.

Tautomeric mixture of 4-azido-6,7-dimethoxy-2-(piperidin-1-yl)quinazoline and 8,9-dimethoxy-5-(piperidin-1-yl)tetrazolo[1,5-*c*]quinazoline (**17b**)

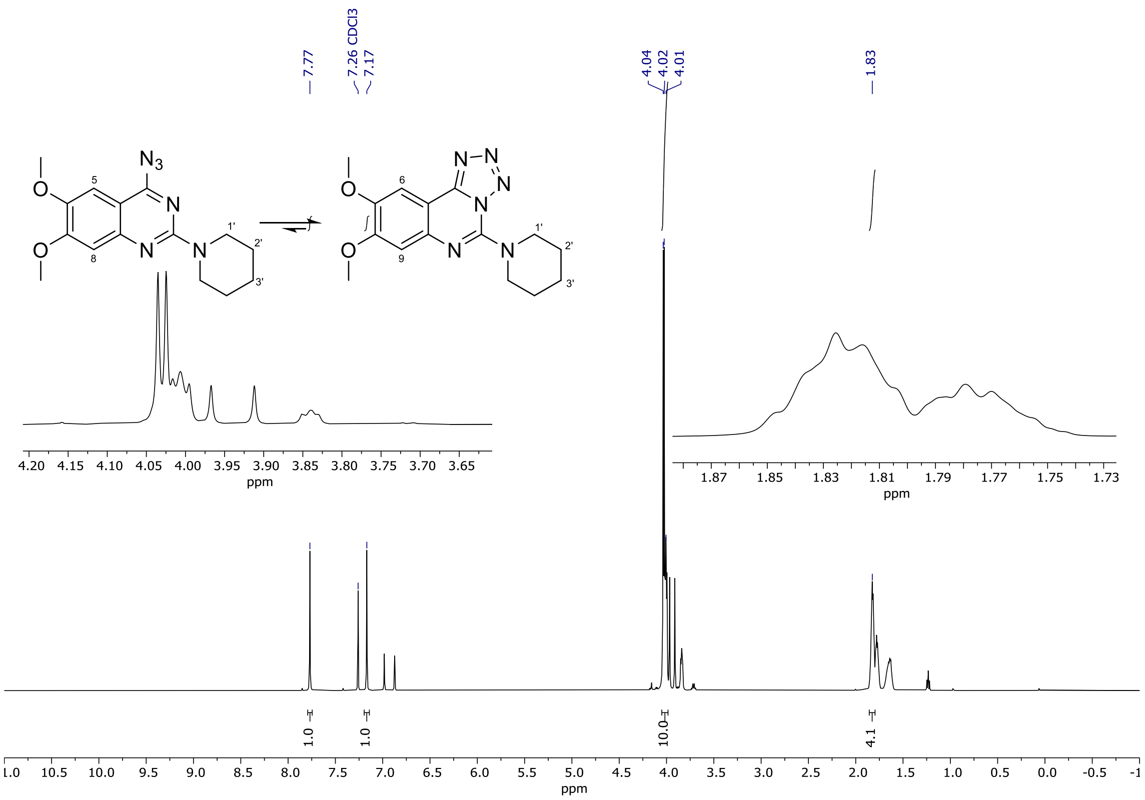

Figure S56: ^{1}H NMR (500 MHz, $CDCl_3$) spectrum.

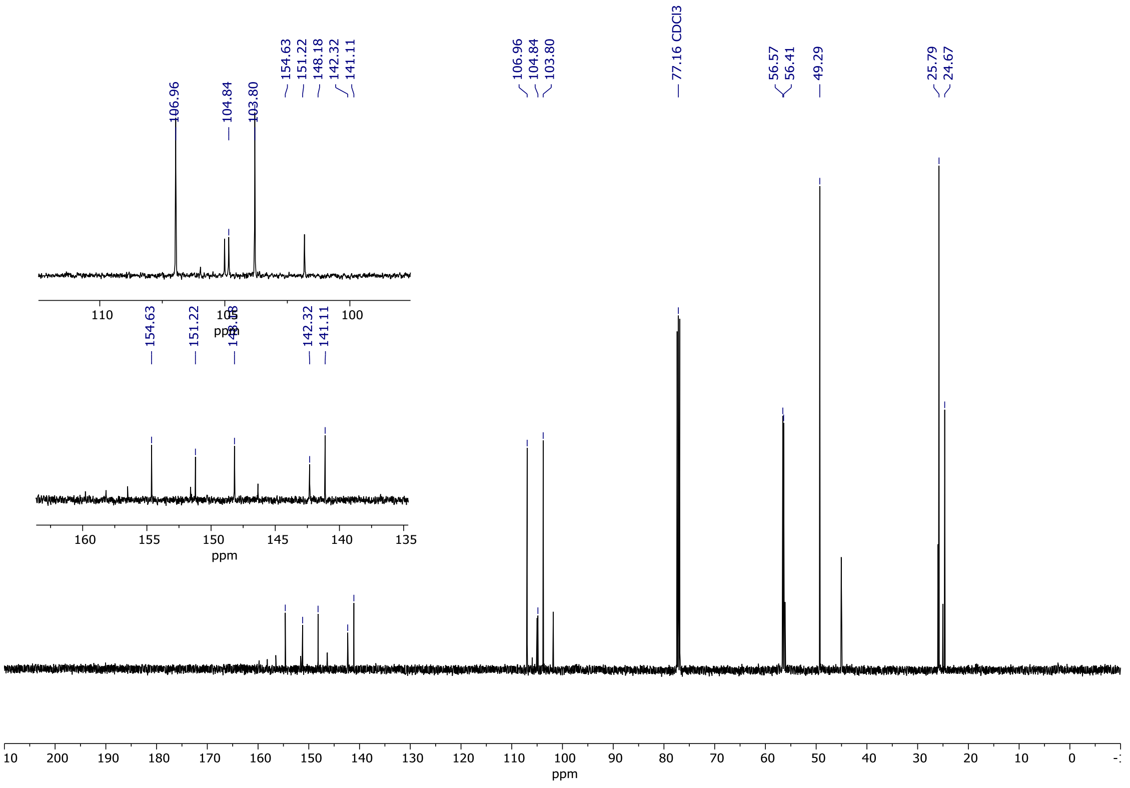

Figure S57: ^{13}C NMR (126 MHz, $CDCl_3$) spectrum.

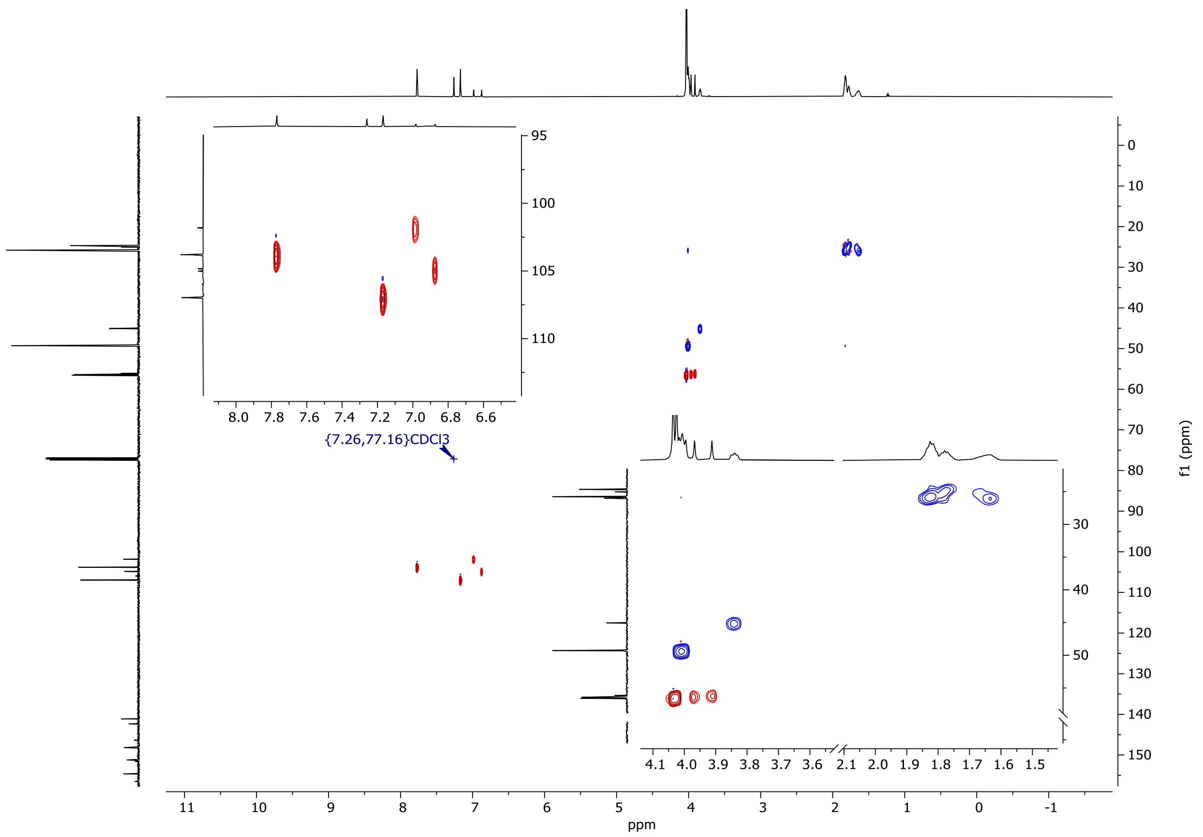

Figure S58: ^{1}H-^{13}C HSQC spectrum.

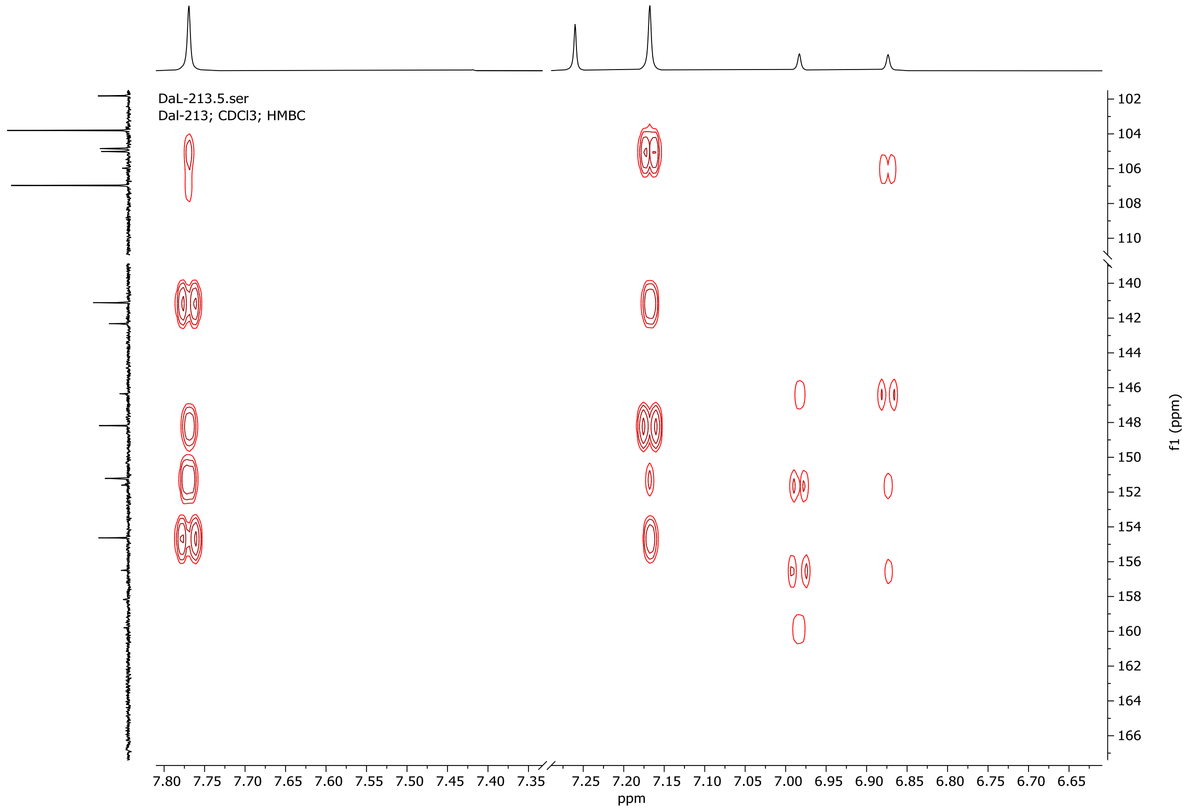

Figure S59: ^{1}H-^{13}C HMBC spectrum.

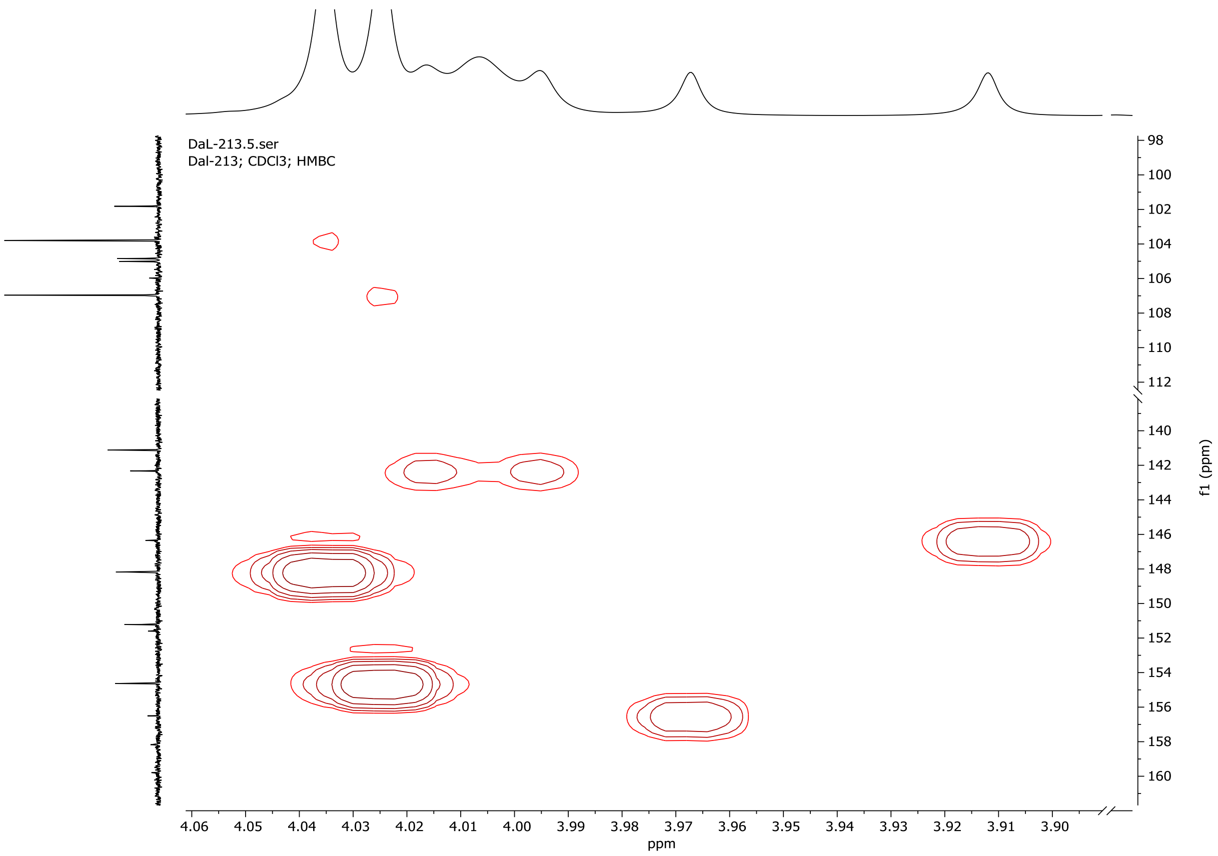

Figure S60: ^{1}H-^{13}C HMBC spectrum.

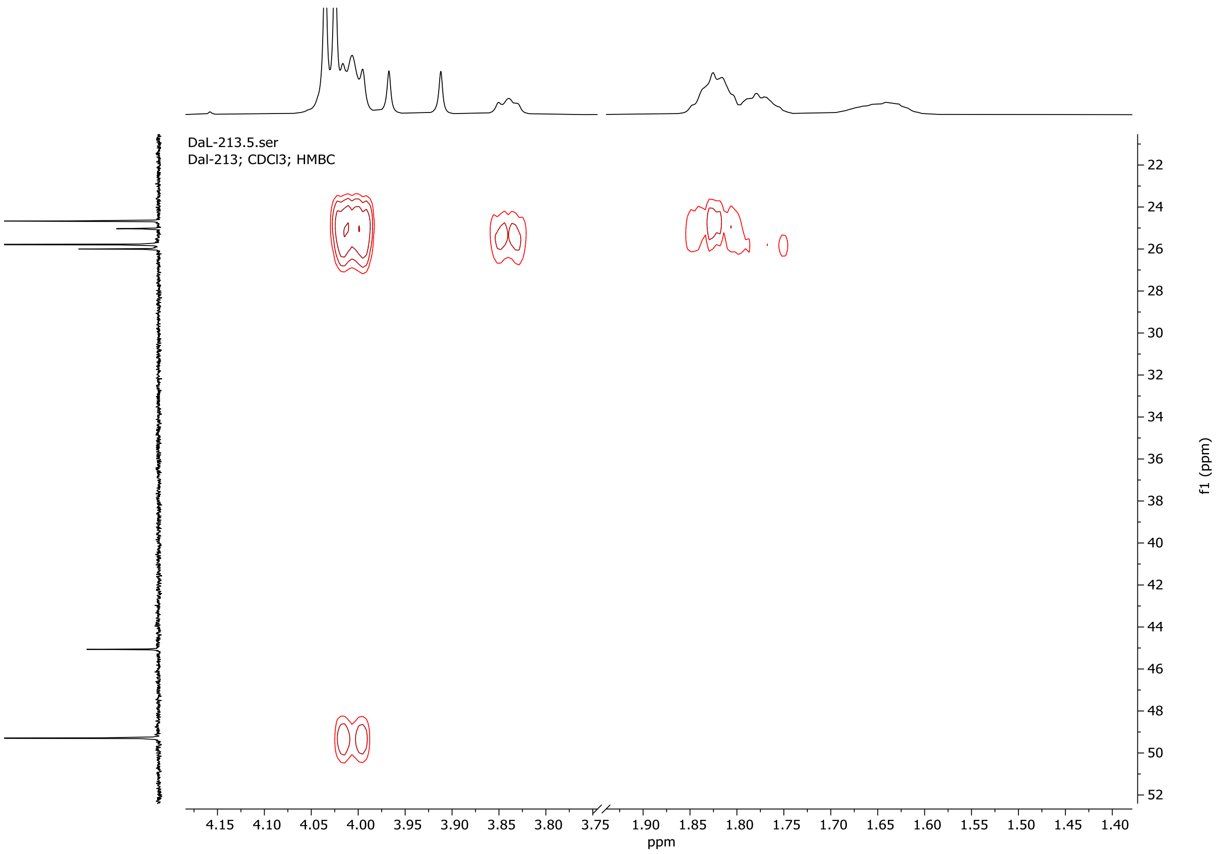

Figure S61: ^{1}H-^{13}C HMBC spectrum.

Tautomeric mixture of 4-azido-6,7-dimethoxy-2-(morphon-1-yl)quinazoline and 8,9-dimethoxy-5-(morphon-1-yl)tetrazolo[1,5-*c*]quinazoline (**17c**)

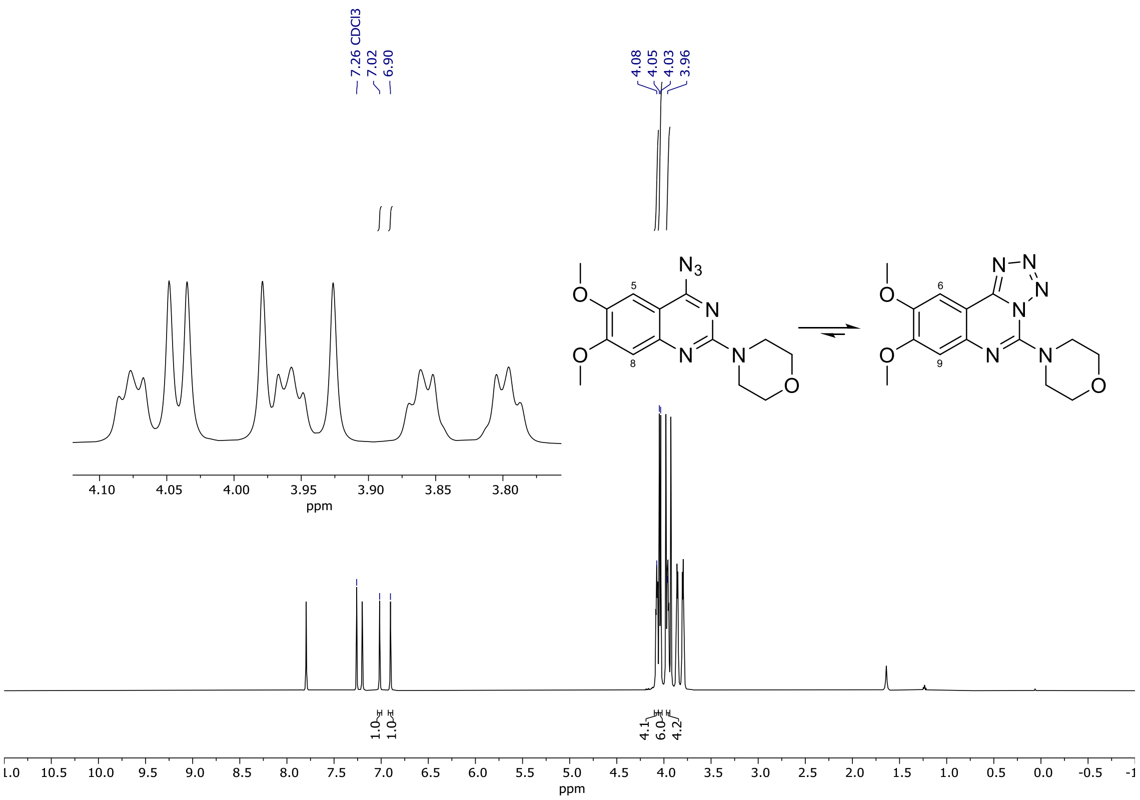

Figure S62: ^{1}H NMR (500 MHz, $CDCl_3$) spectrum.

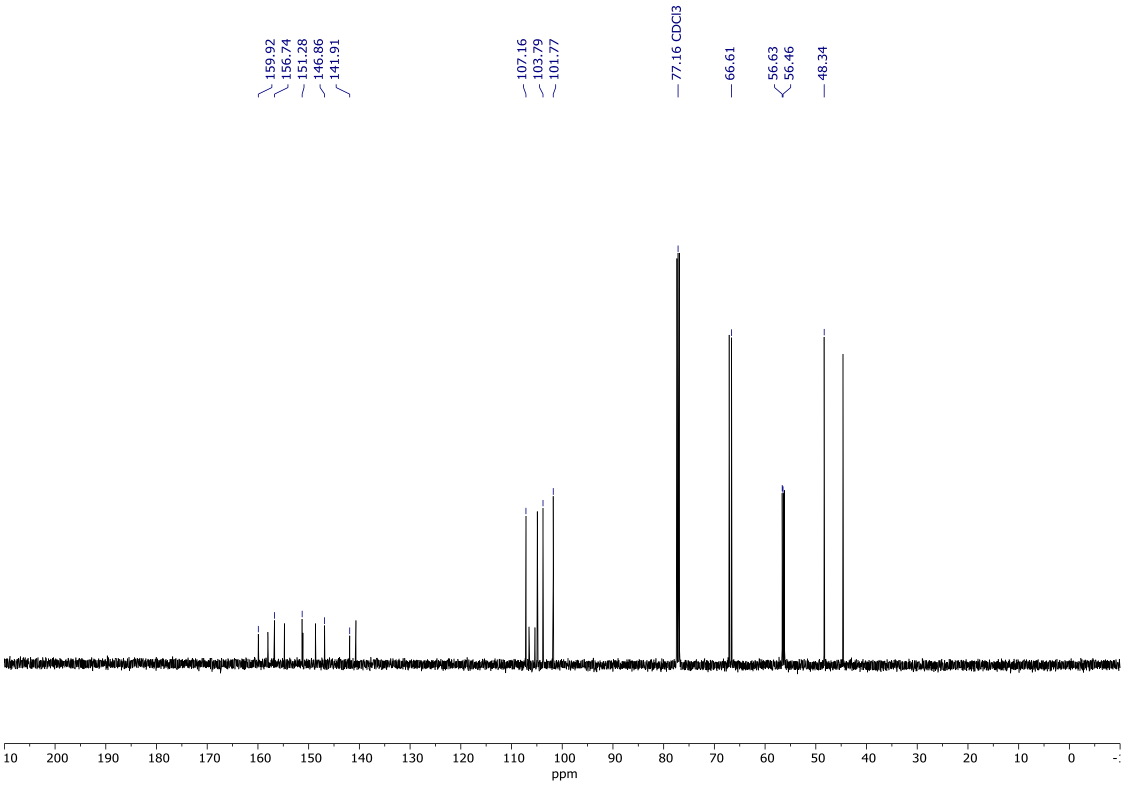

Figure S63: ^{13}C NMR (126 MHz, $CDCl_3$) spectrum.

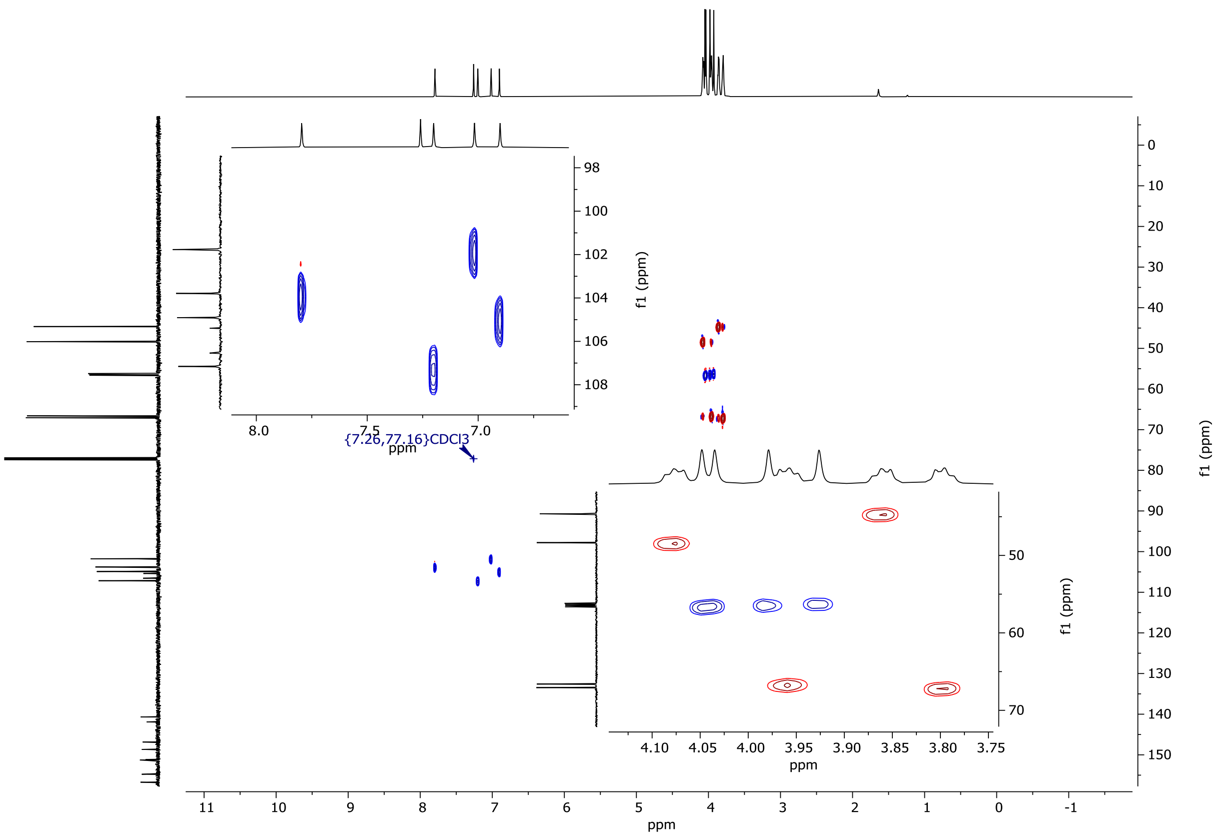

Figure S64: ^{1}H-^{13}C HSQC spectrum.

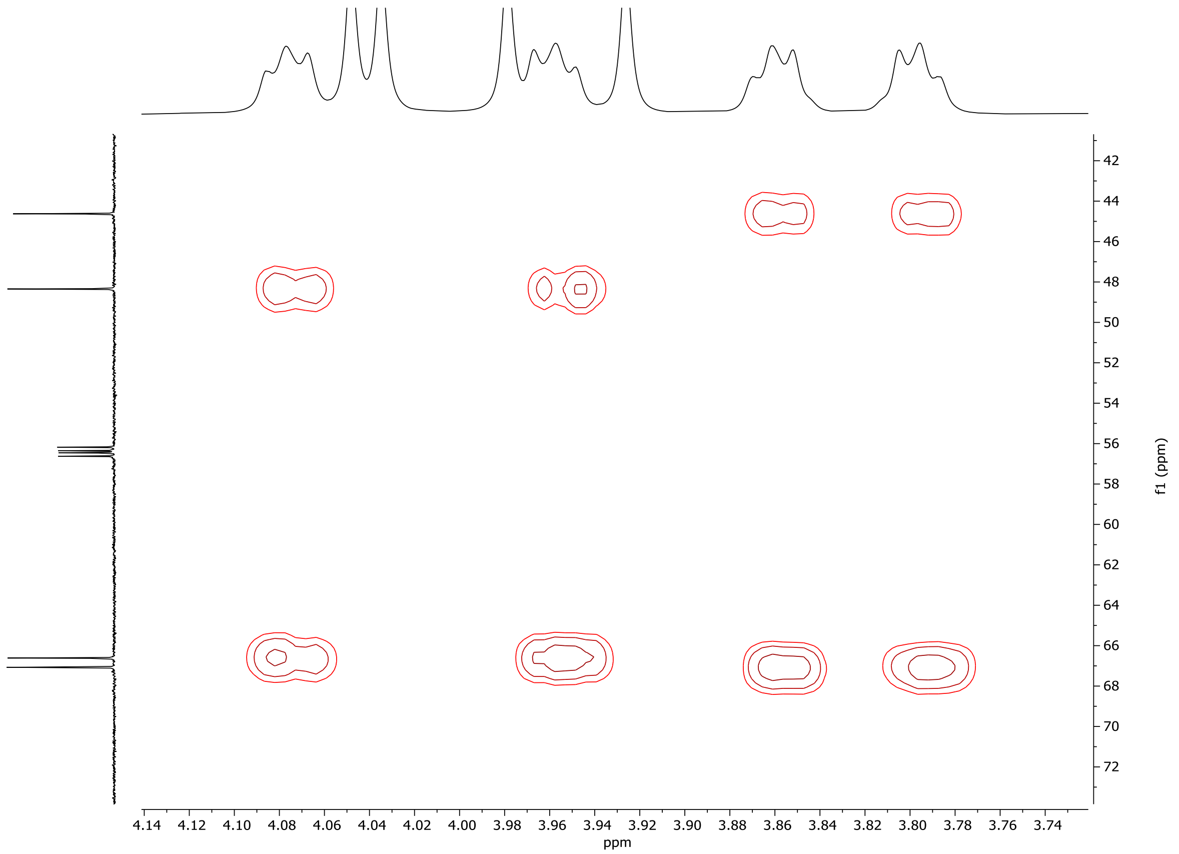

Figure S65: ^{1}H-^{13}C HMBC spectrum.

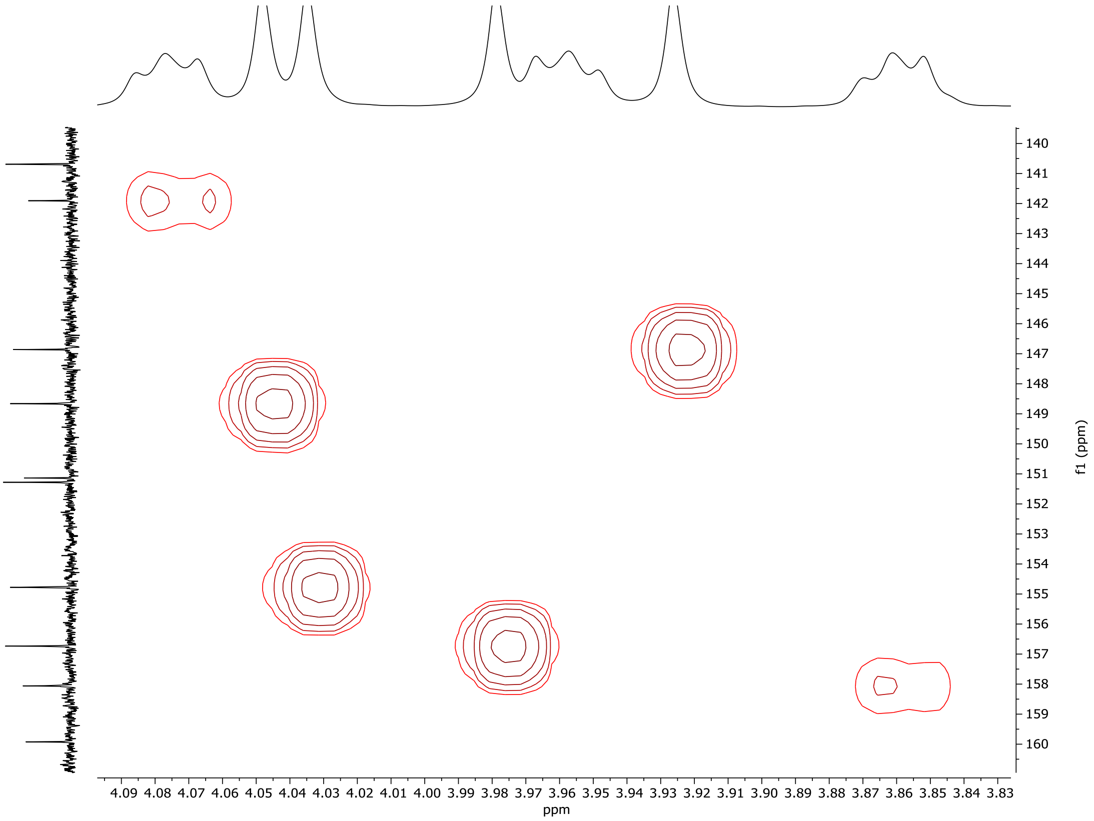

Figure S66: ^{1}H-^{13}C HMBC spectrum.

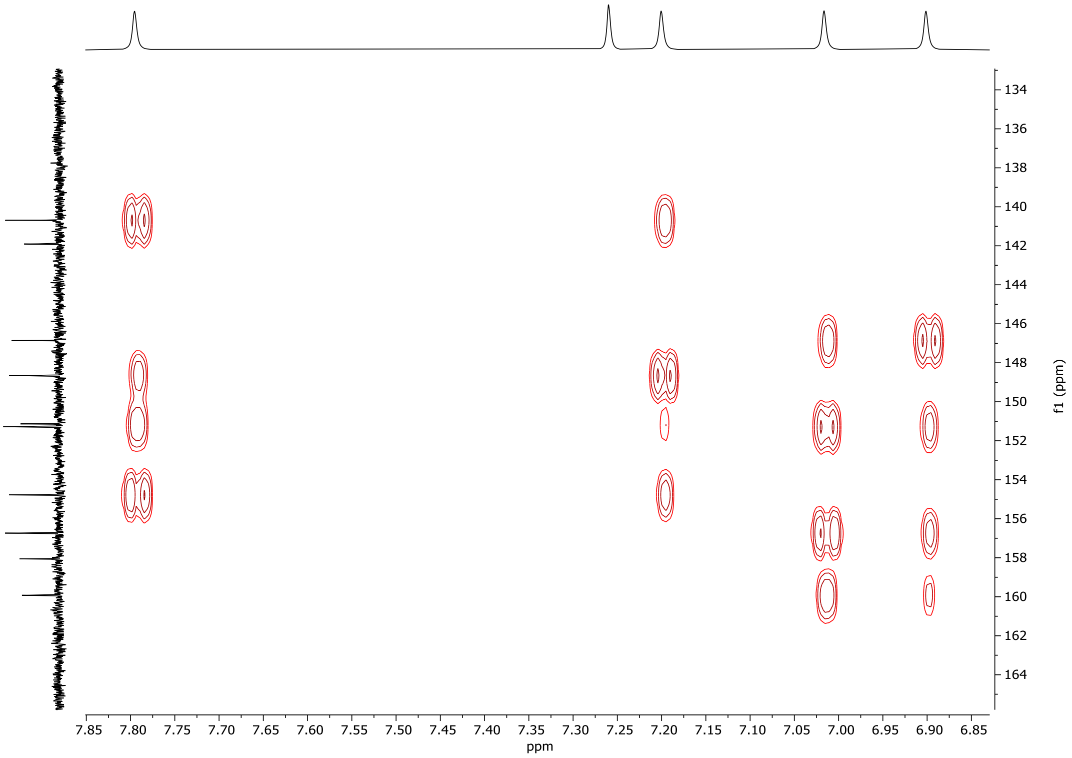

Figure S67: ^{1}H-^{13}C HMBC spectrum.

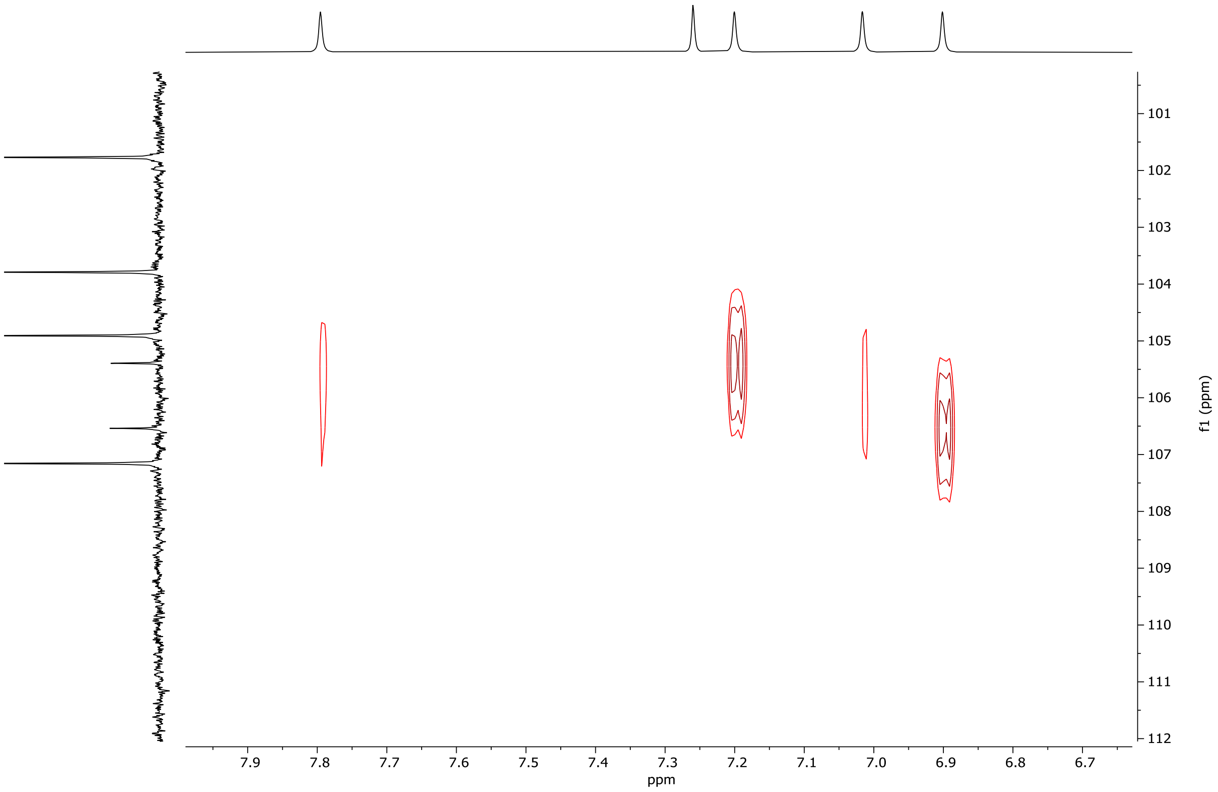

Figure S68: ^{1}H-^{13}C HMBC spectrum.

Tautomeric mixture of 4-azido-6,7-dimethoxy-2-(4-methylpiperazin-1-yl)quinazoline and 8,9-dimethoxy-5-(4-methylpiperazin-1-yl)tetrazolo[1,5-*c*]quinazoline (**17d**)

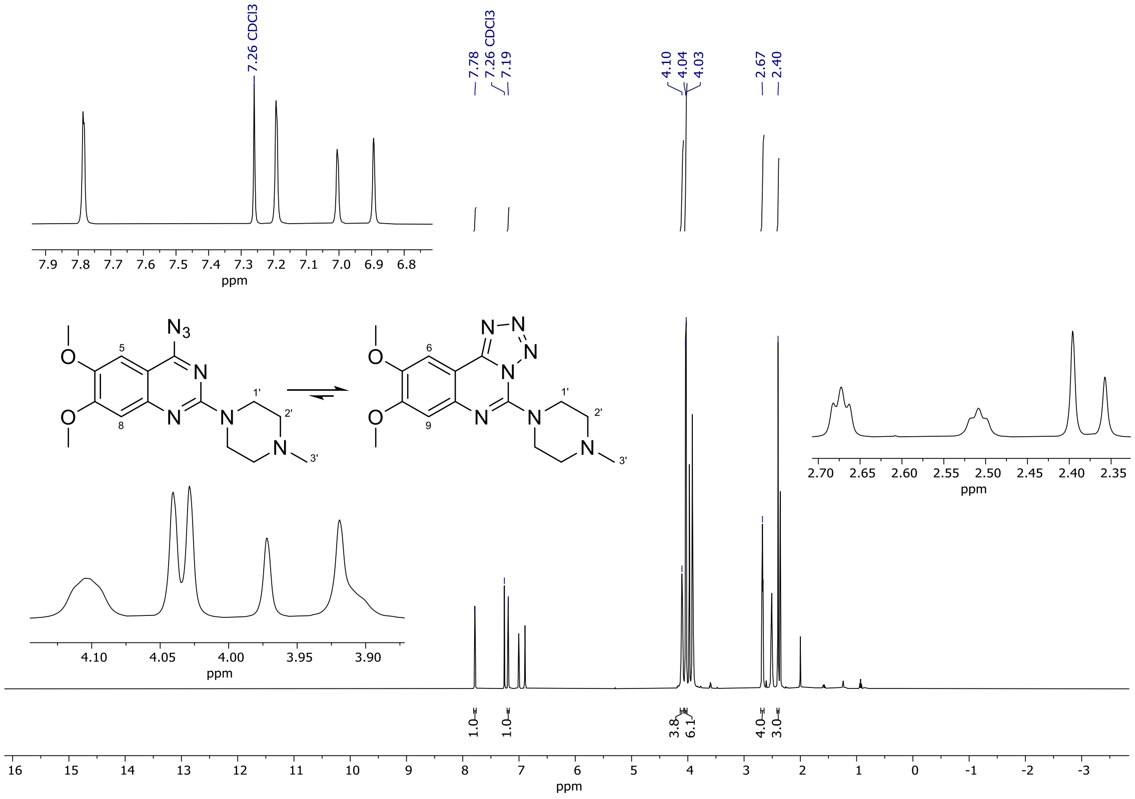

Figure S69: ^{1}H NMR (500 MHz, $CDCl_3$) spectrum.

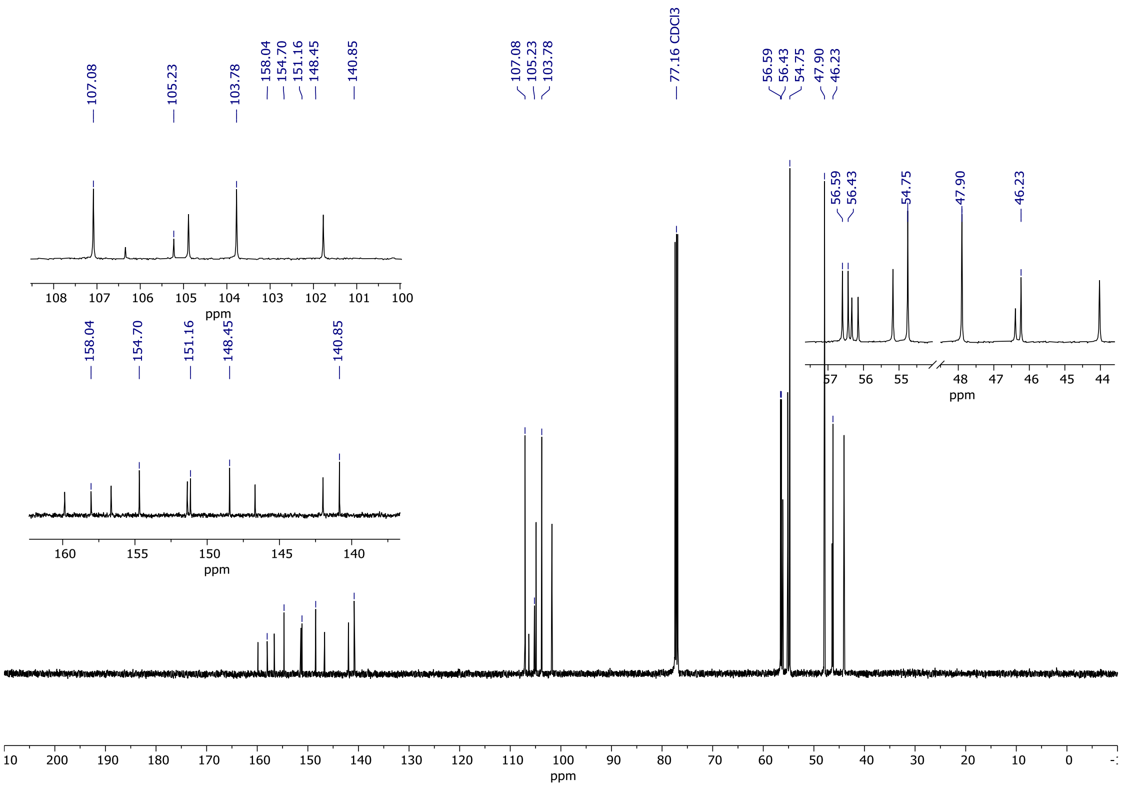

Figure S70: ^{13}C NMR (126 MHz, $CDCl_3$) spectrum.

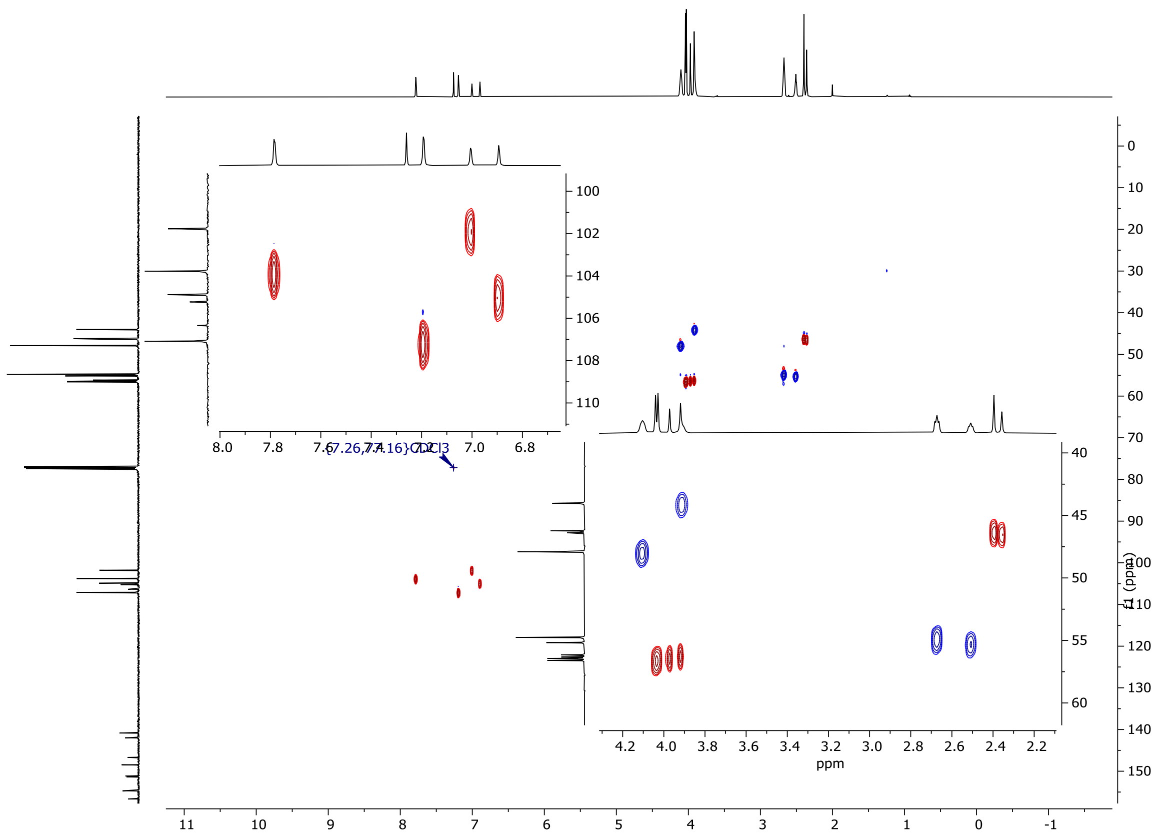

Figure S71: ^{1}H-^{13}C HSQC spectrum.

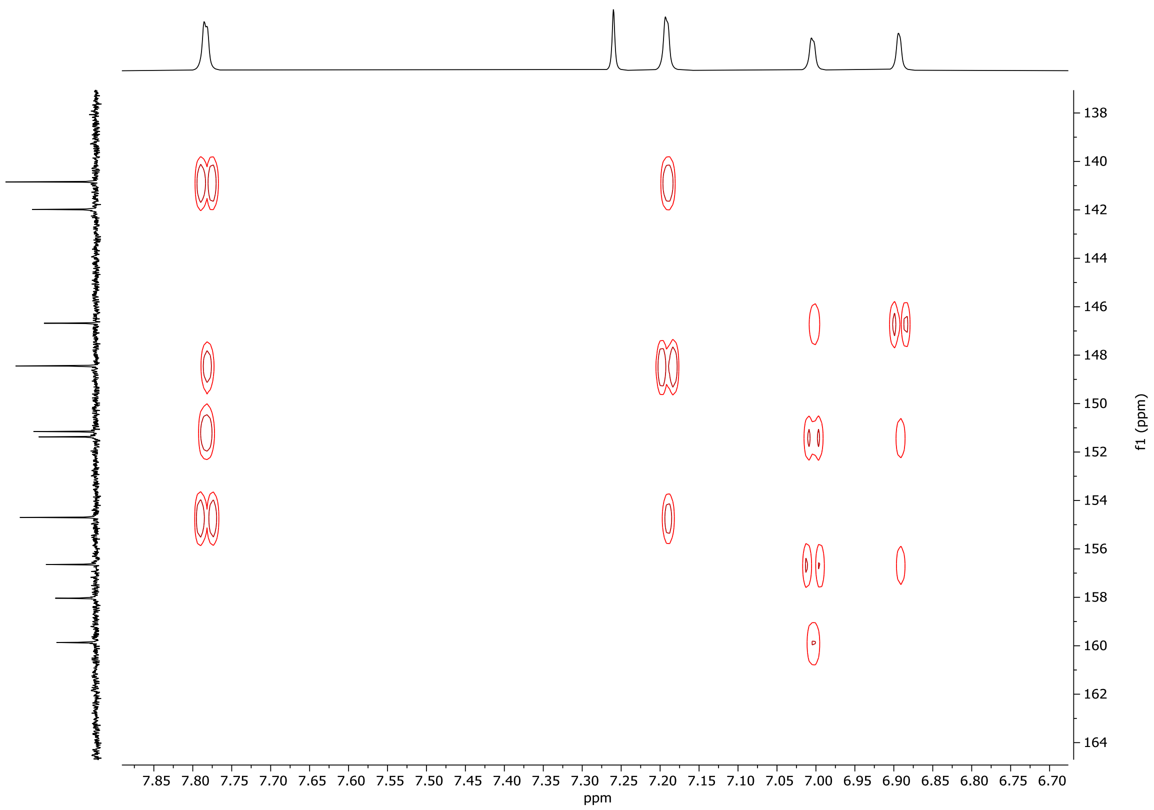

Figure S72: ^{1}H-^{13}C HMBC spectrum.

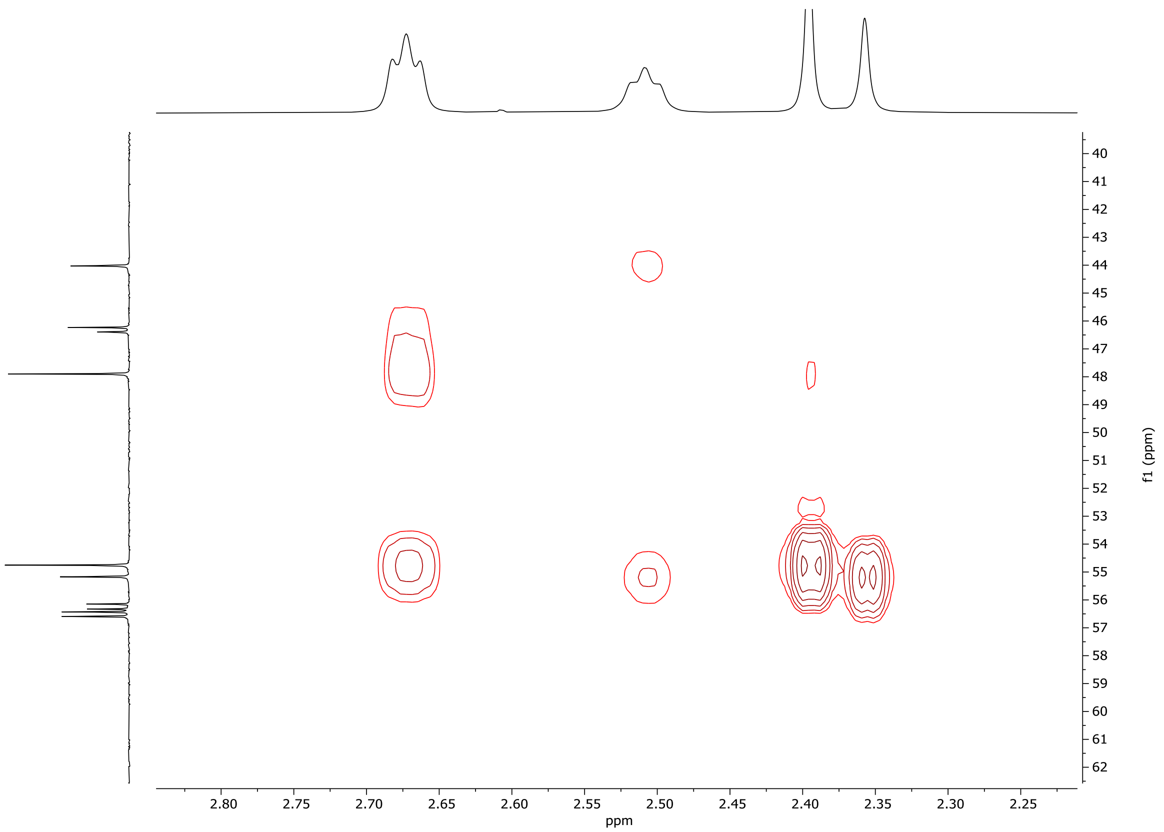

Figure S73: ^{1}H-^{13}C HMBC spectrum.

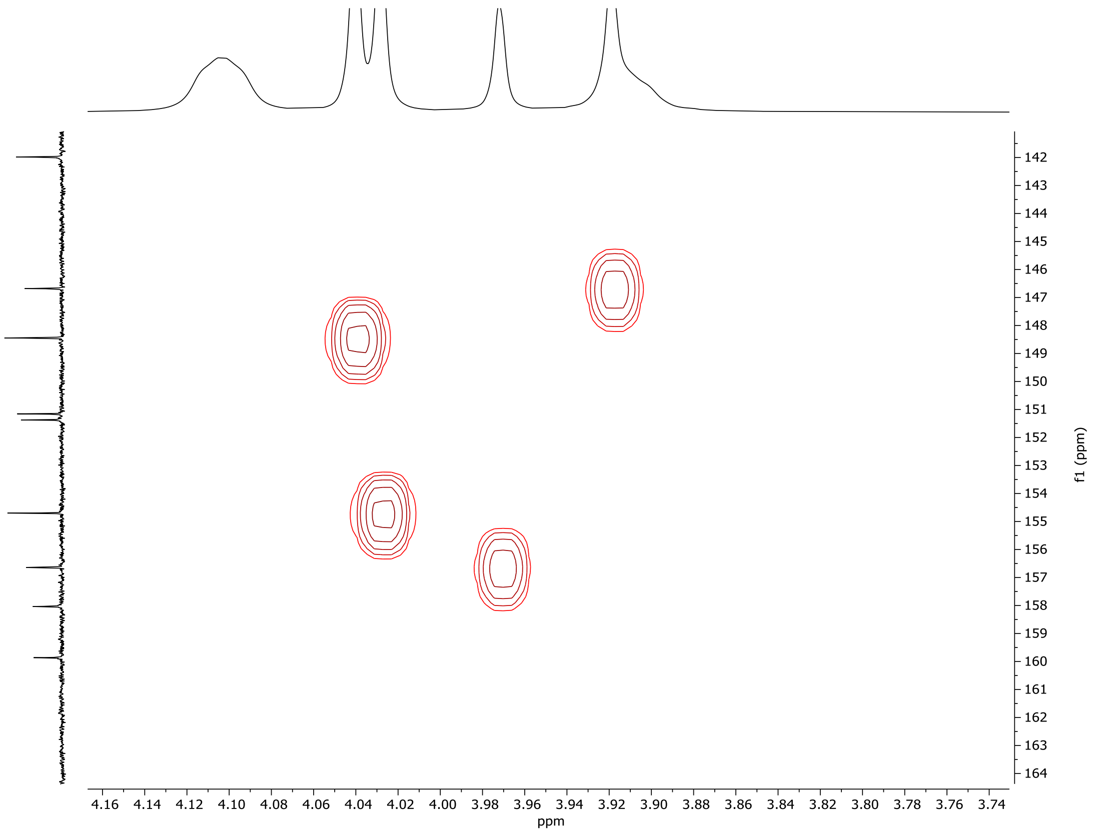

Figure S74: ^{1}H-^{13}C HSQC spectrum.

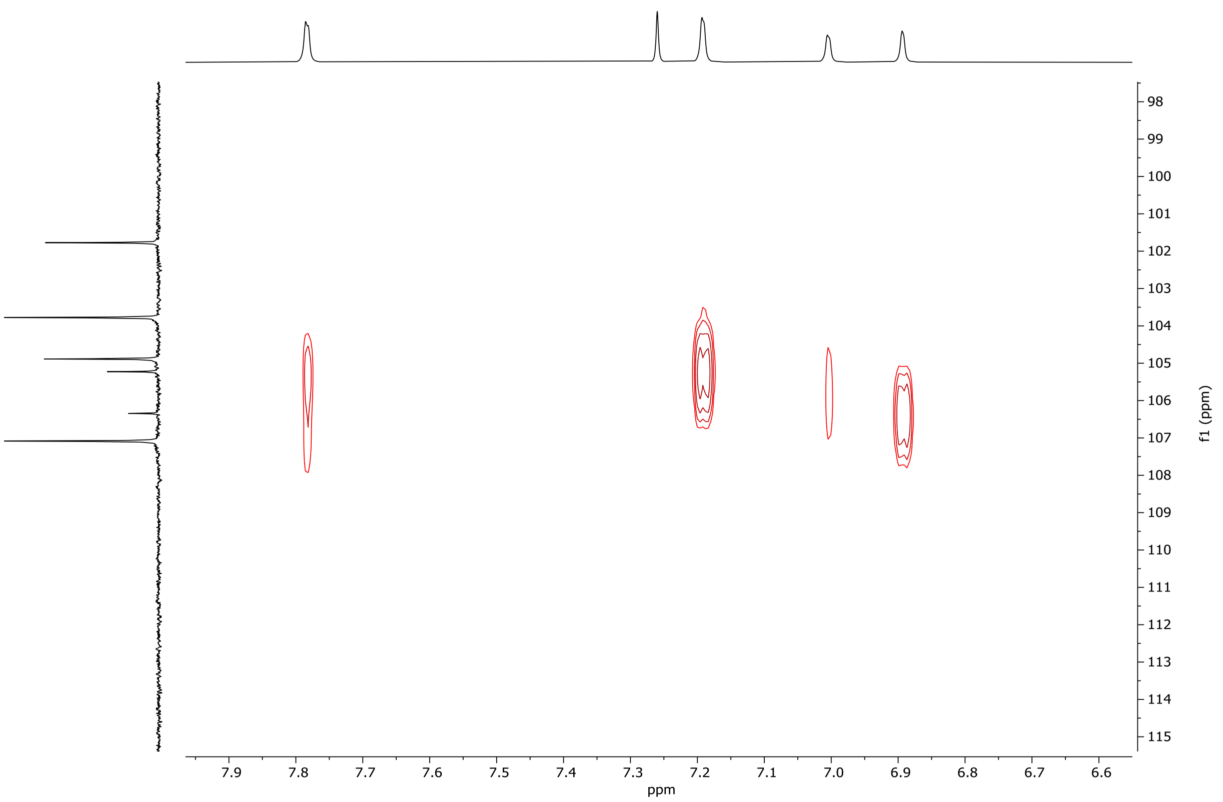

Figure S75: ^{1}H-^{13}C HSQC spectrum.

Tautomeric mixture of (4-(4-azido-6,7-dimethoxyquinazolin-2-yl)piperazin-1-yl)(tetrahydrofuran-2-yl)methanone and (4-(8,9-dimethoxytetrazolo[1,5-*c*]quinazolin-5-yl)piperazin-1-yl)(tetrahydrofuran-2-yl)methanone (**17e**)

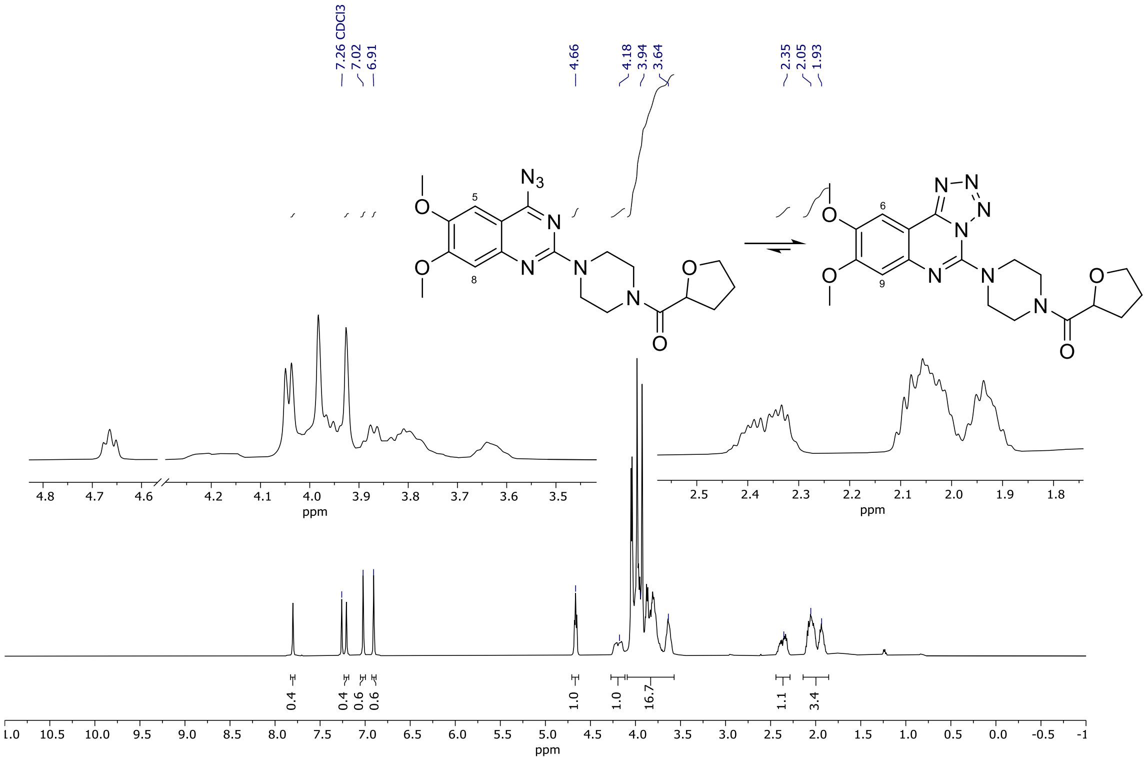

Figure S76: ^{1}H NMR (500 MHz, $CDCl_3$) spectrum.

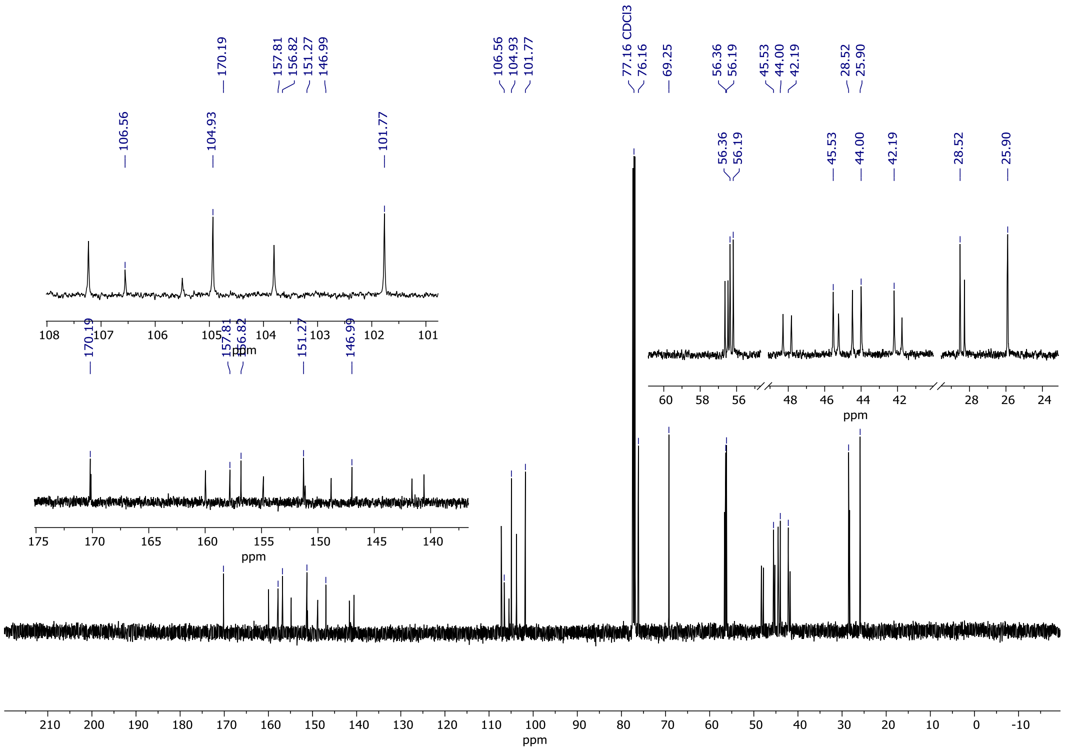

Figure S77: ^{13}C NMR (126 MHz, $CDCl_3$) spectrum.

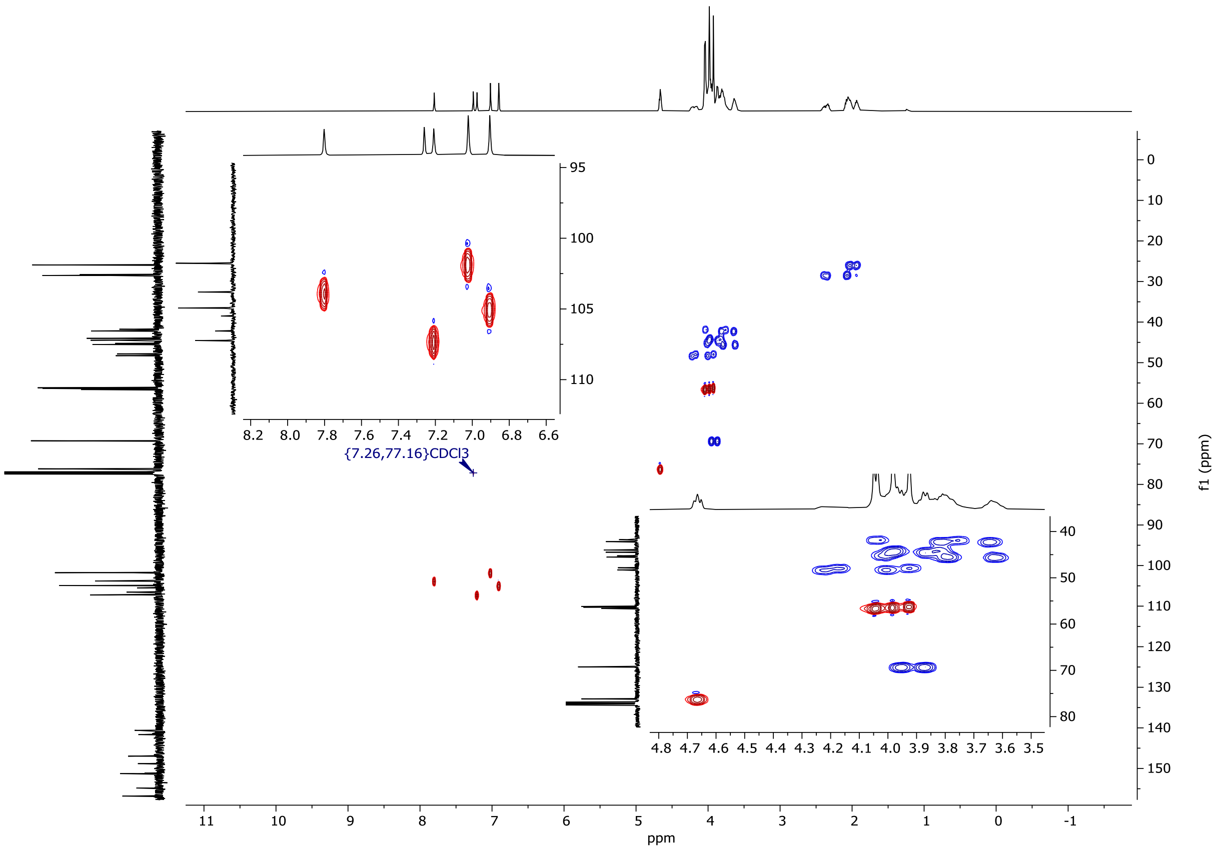

Figure S78: ^{1}H-^{13}C HSQC spectrum.

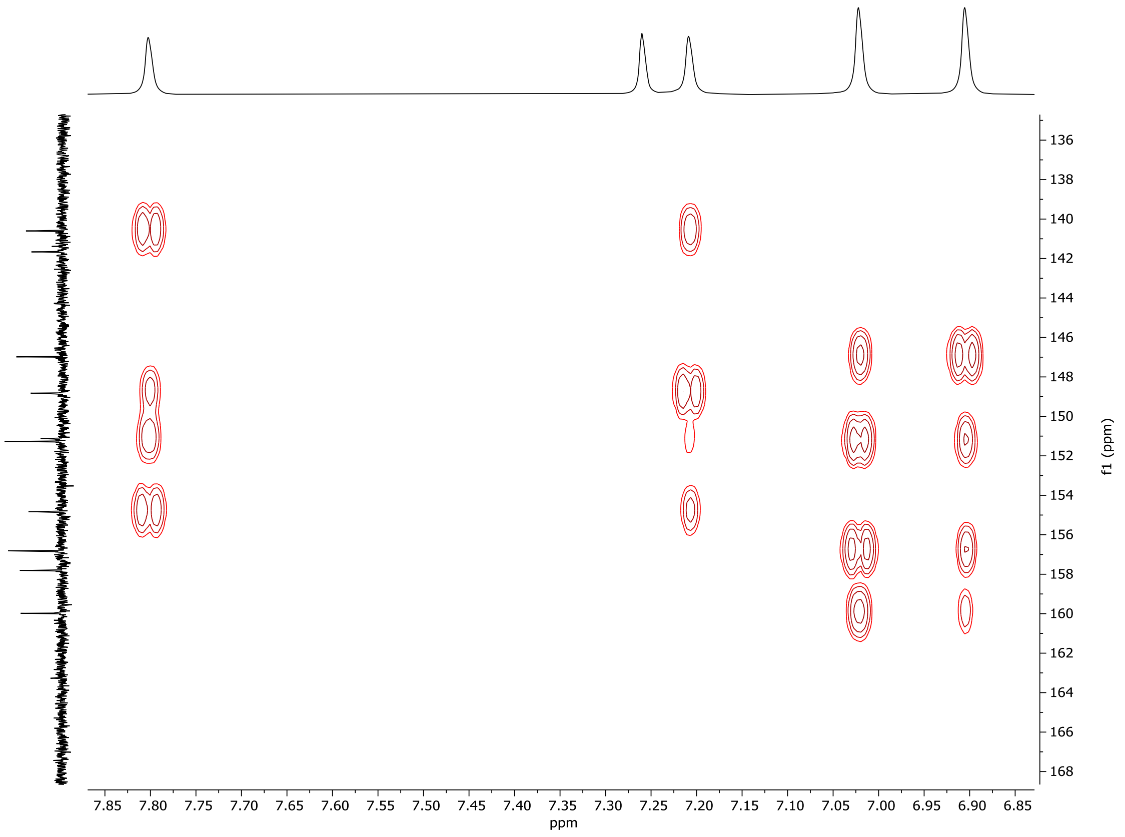

Figure S79: ^{1}H-^{13}C HMBC spectrum.

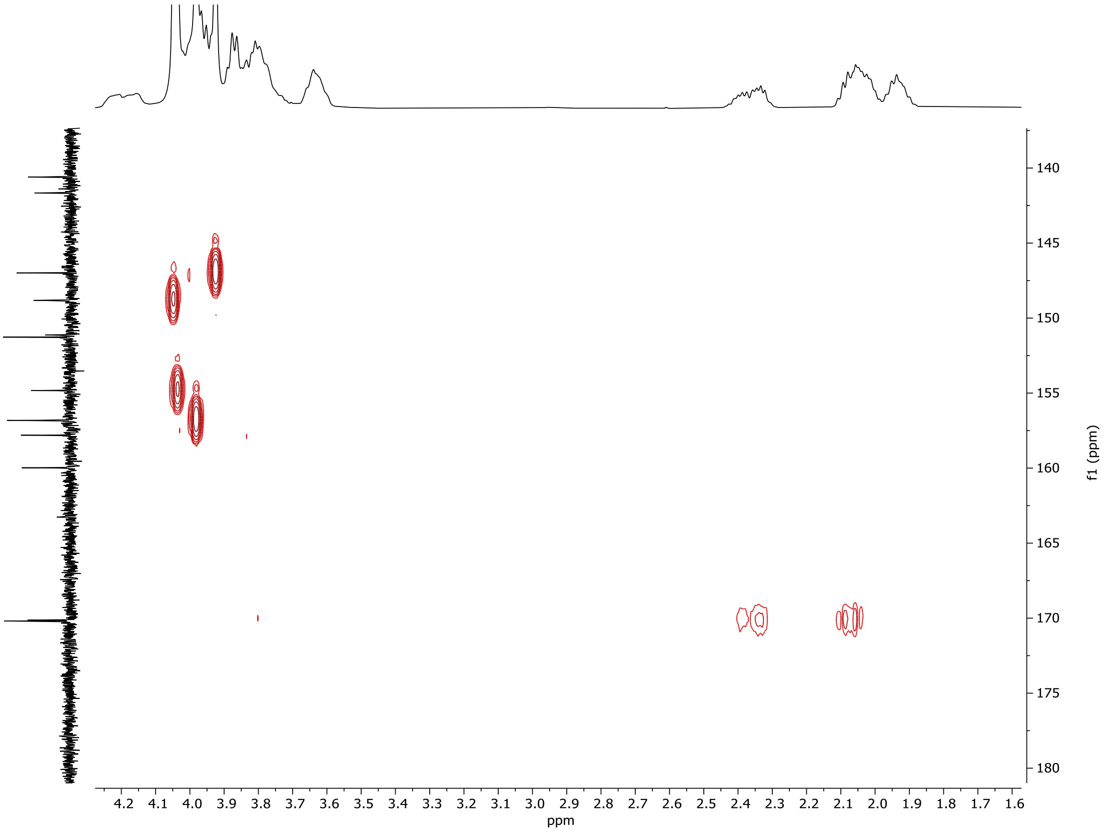

Figure S80: ^{1}H-^{13}C HMBC spectrum.

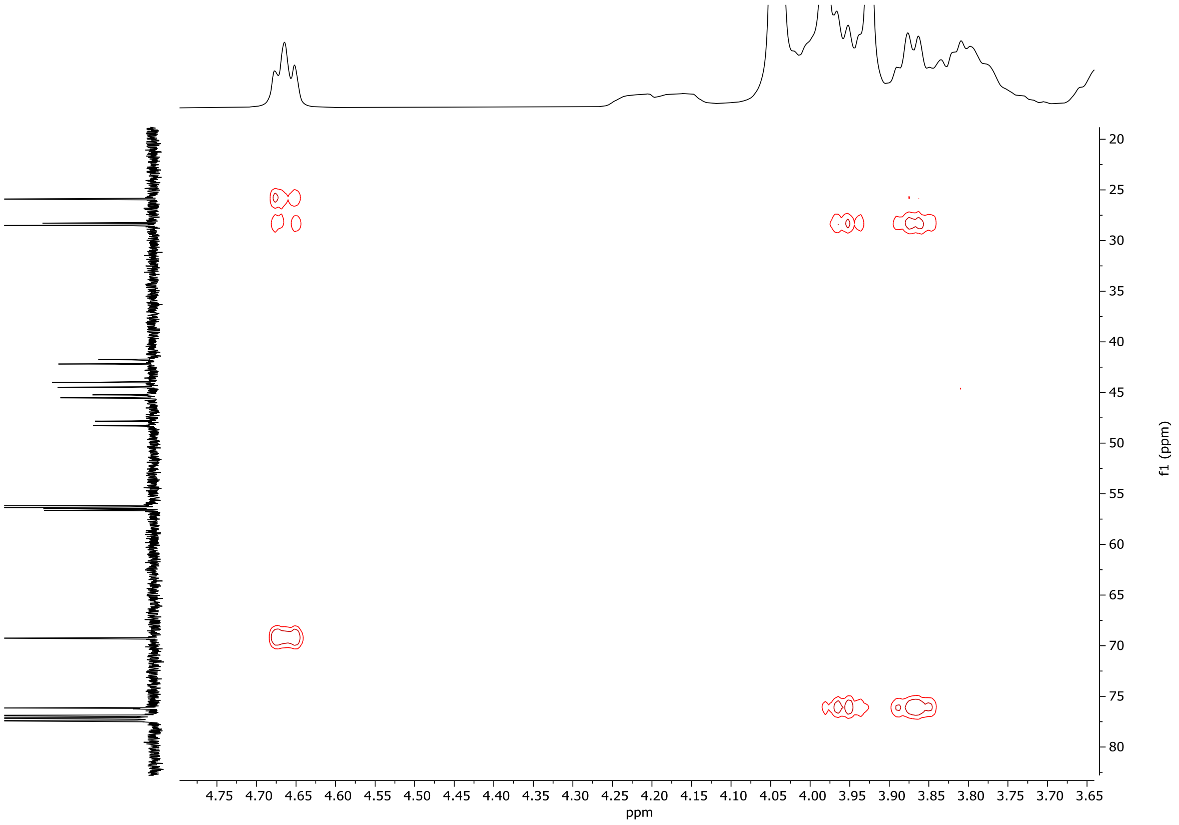

Figure S81: ^{1}H-^{13}C HMBC spectrum.

Tautomeric mixture of (4-(4-azido-6,7-dimethoxyquinazolin-2-yl)piperazin-1-yl)(furan-2-yl)methanone and (4-(8,9-dimethoxytetrazolo[1,5-*c*]quinazolin-5-yl)piperazin-1-yl)(furan-2-yl)methanone (**17f**)

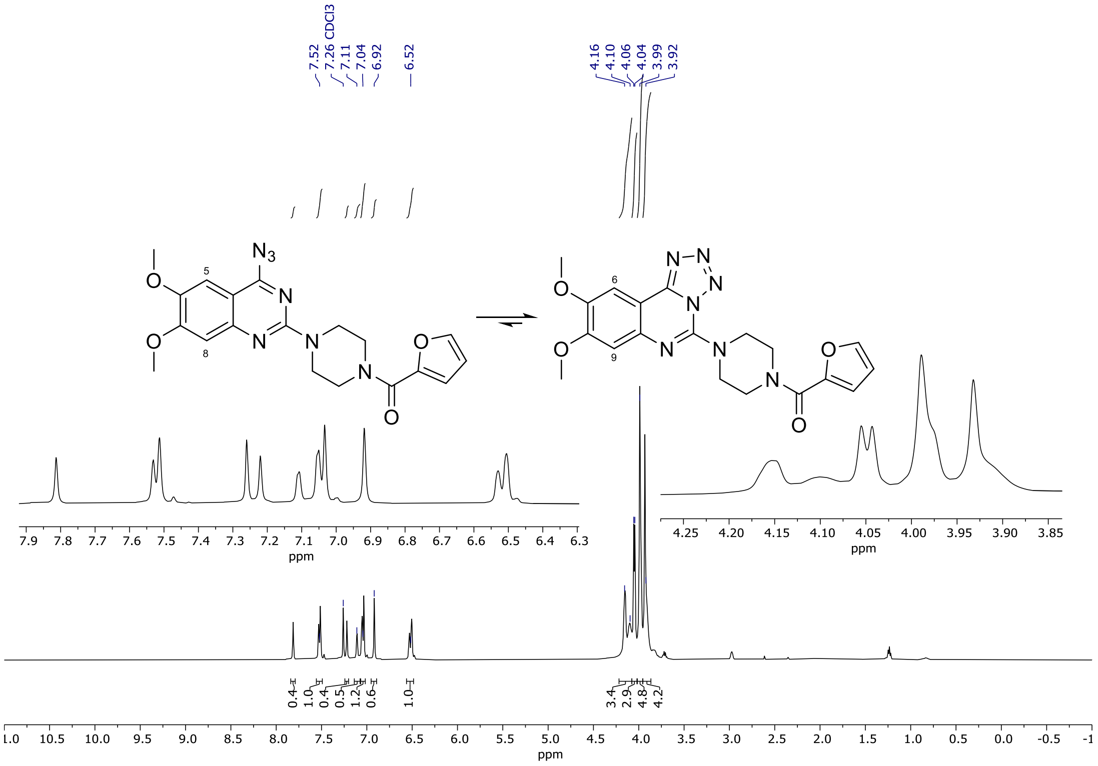

Figure S82: ^{1}H (500 MHz, $CDCl_3$) spectrum.

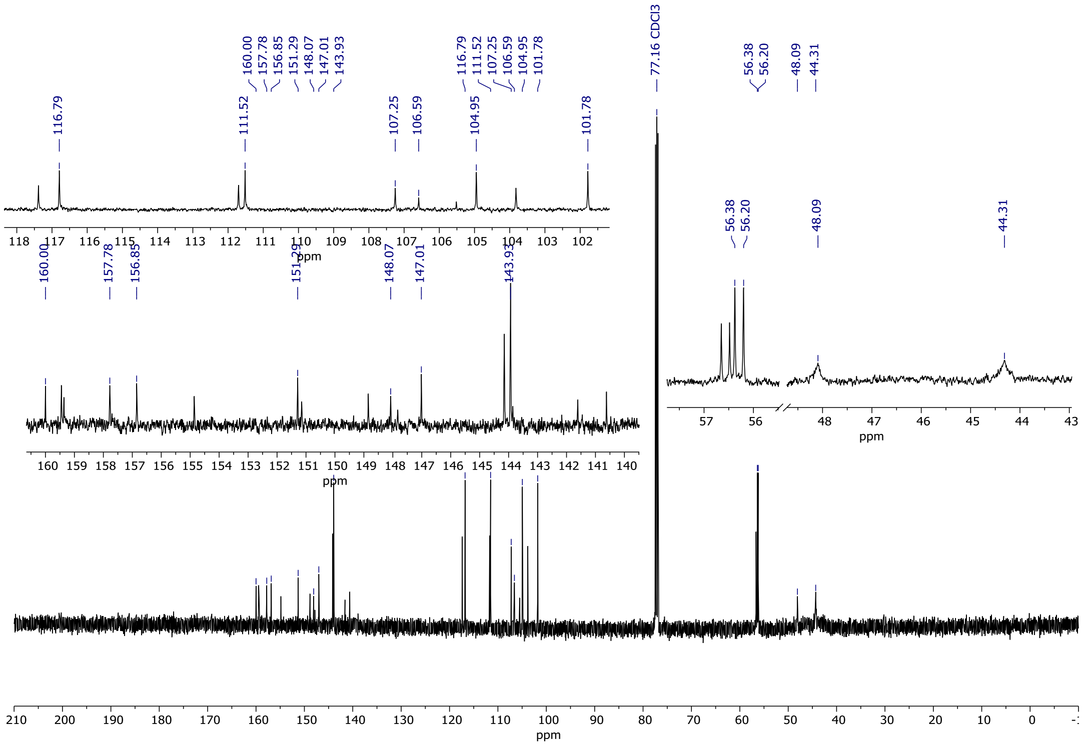

Figure S83: ^{13}C NMR (126 MHz, $CDCl_3$) spectrum.

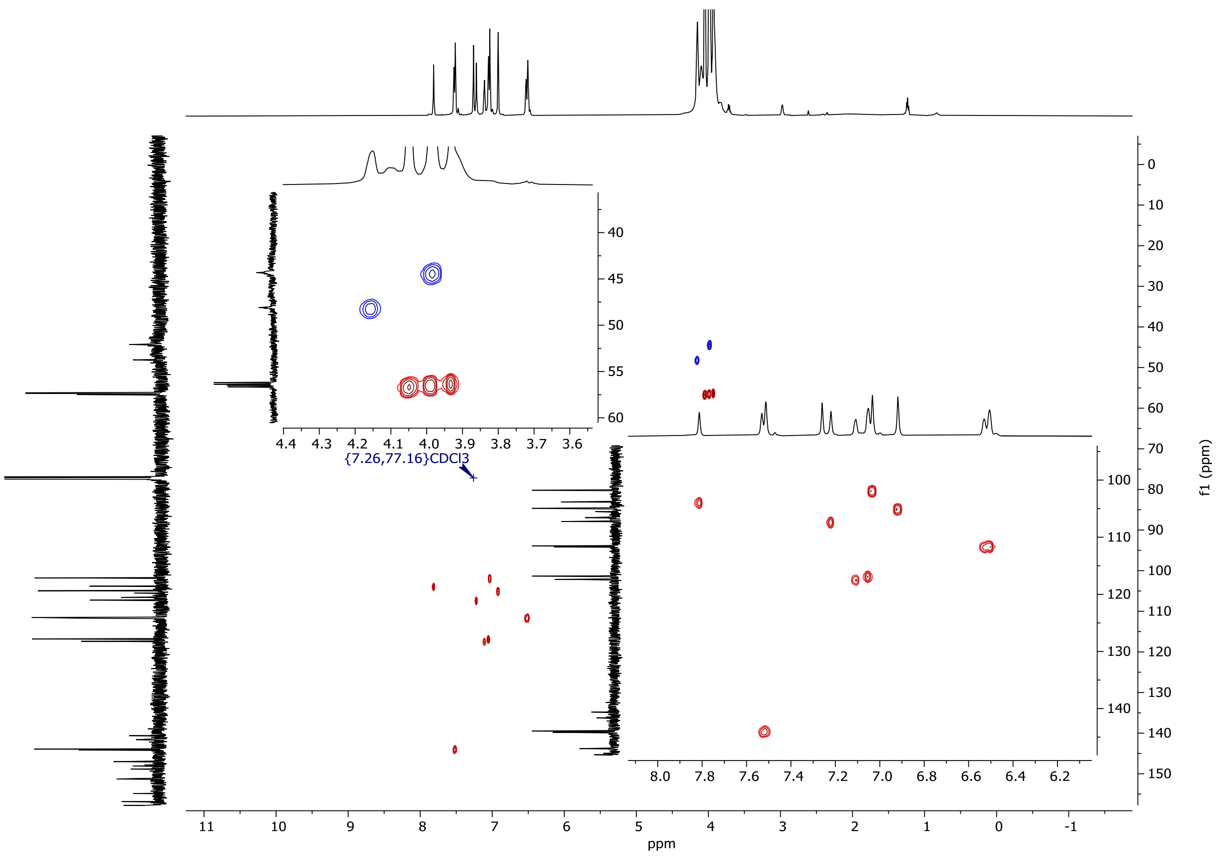

Figure S84: ^{1}H-^{13}C HSQC spectrum.

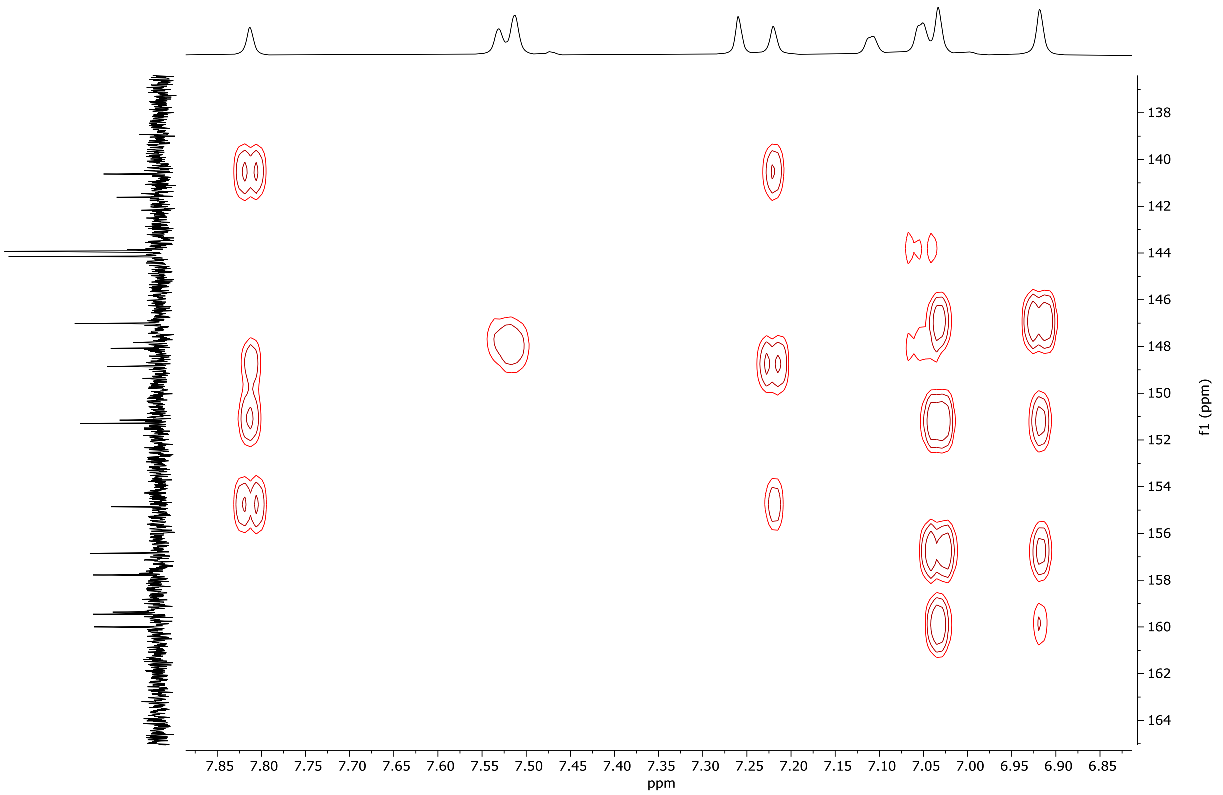

Figure S85: ^{1}H-^{13}C HMBC spectrum.

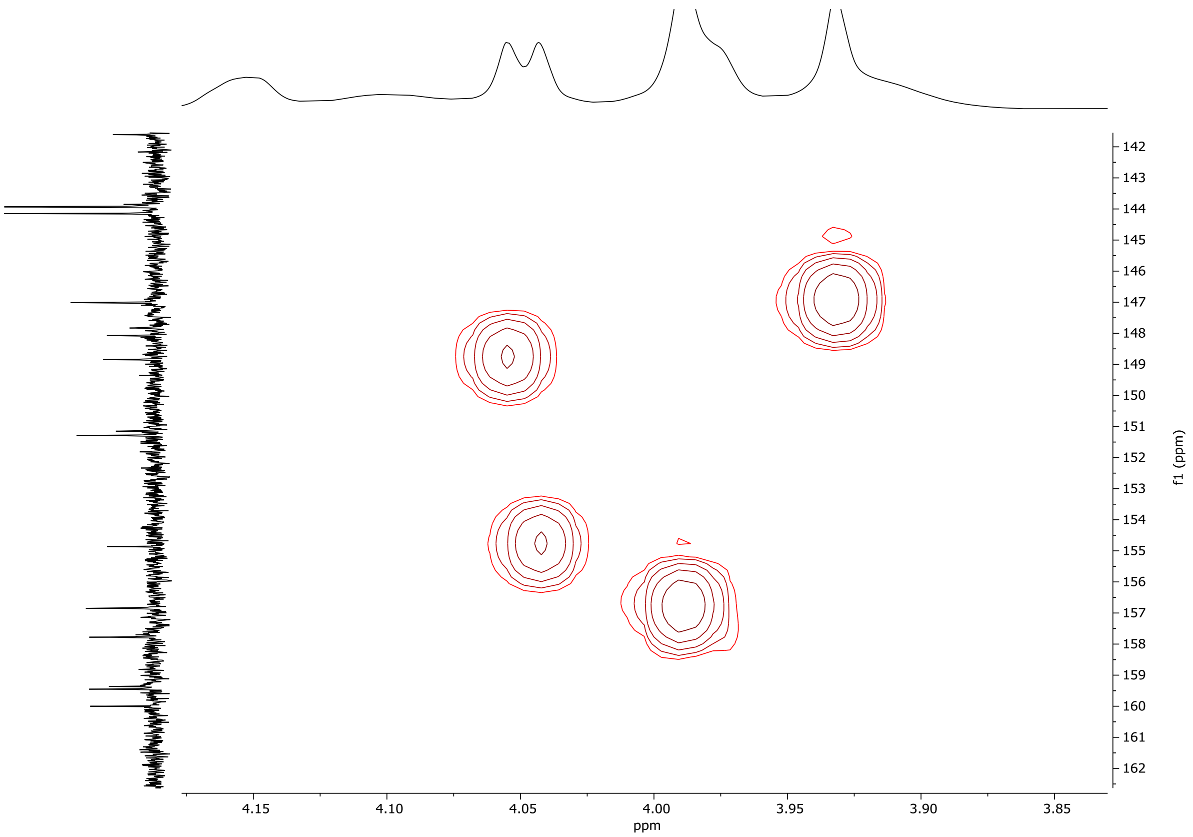

Figure S86: ^{1}H-^{13}C HMBC spectrum.

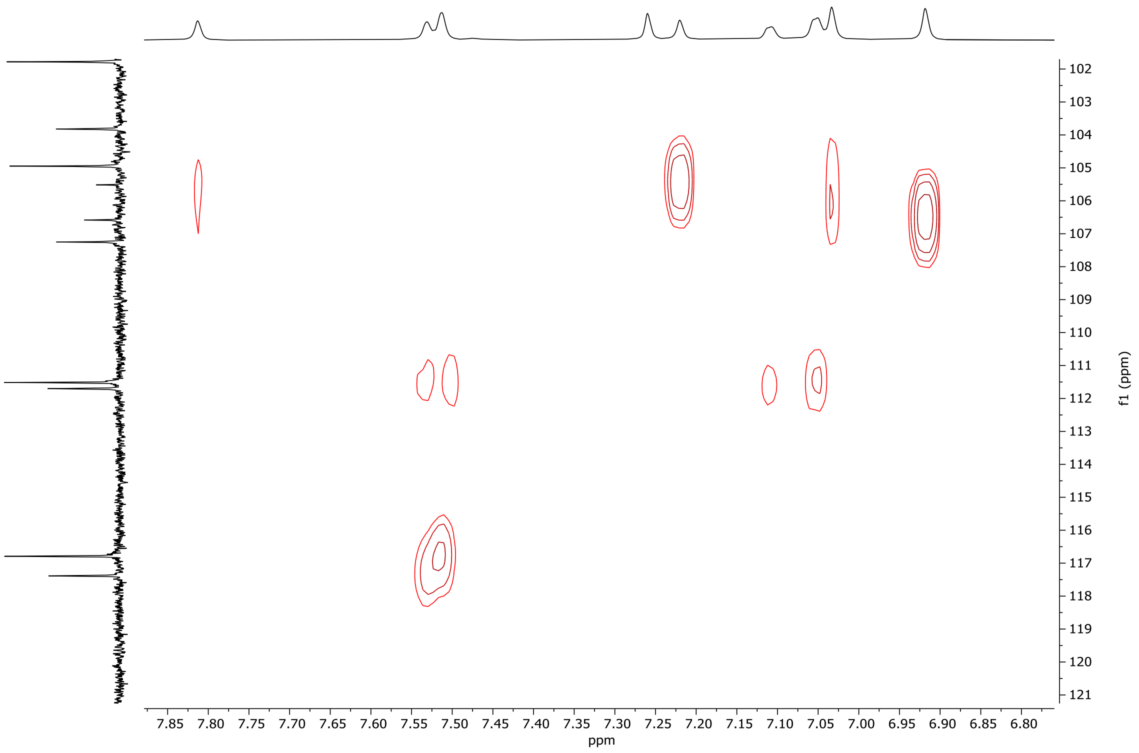

Figure S87: ^{1}H-^{13}C HMBC spectrum.

6,7-Dimethoxy-4-(4-phenyl-1*H*-1,2,3-triazol-1-yl)-2-(pyrrolidin-1-yl)quinazoline (**20a**)

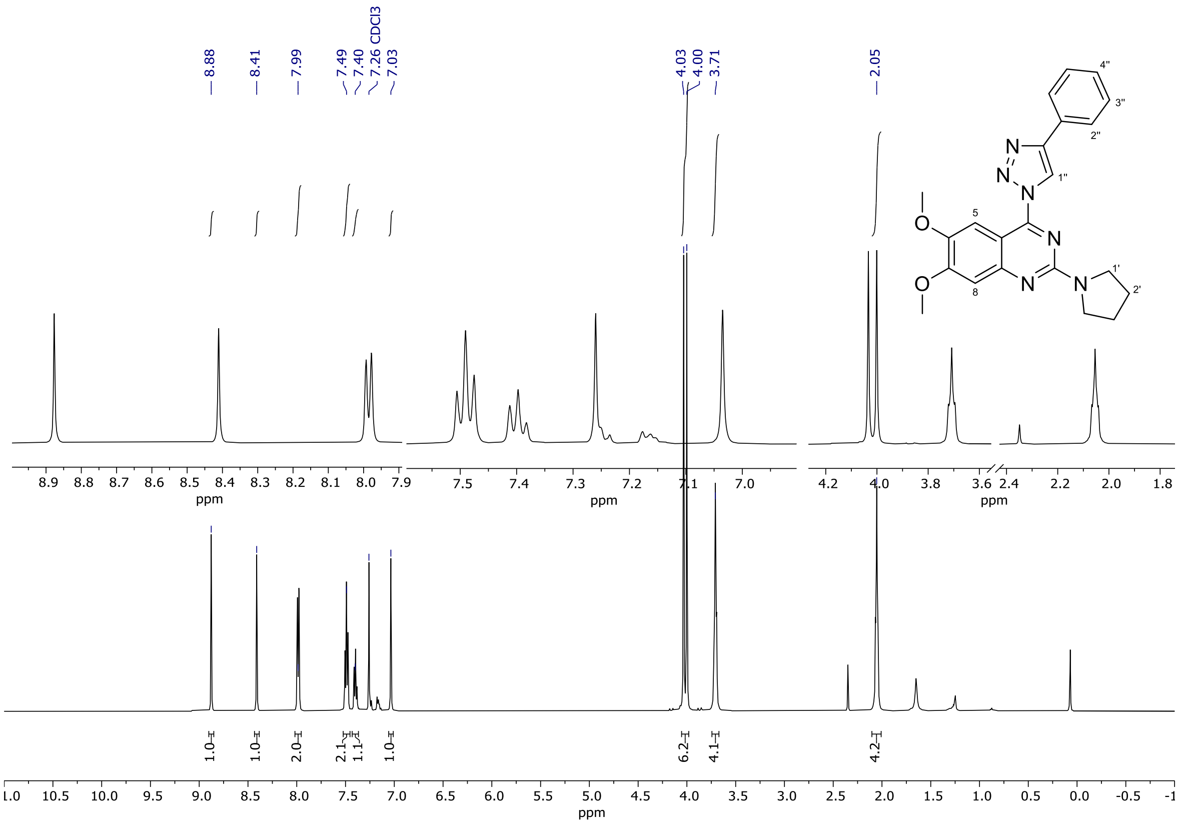

Figure S88: ^{1}H NMR (500 MHz, $CDCl_3$) spectrum.

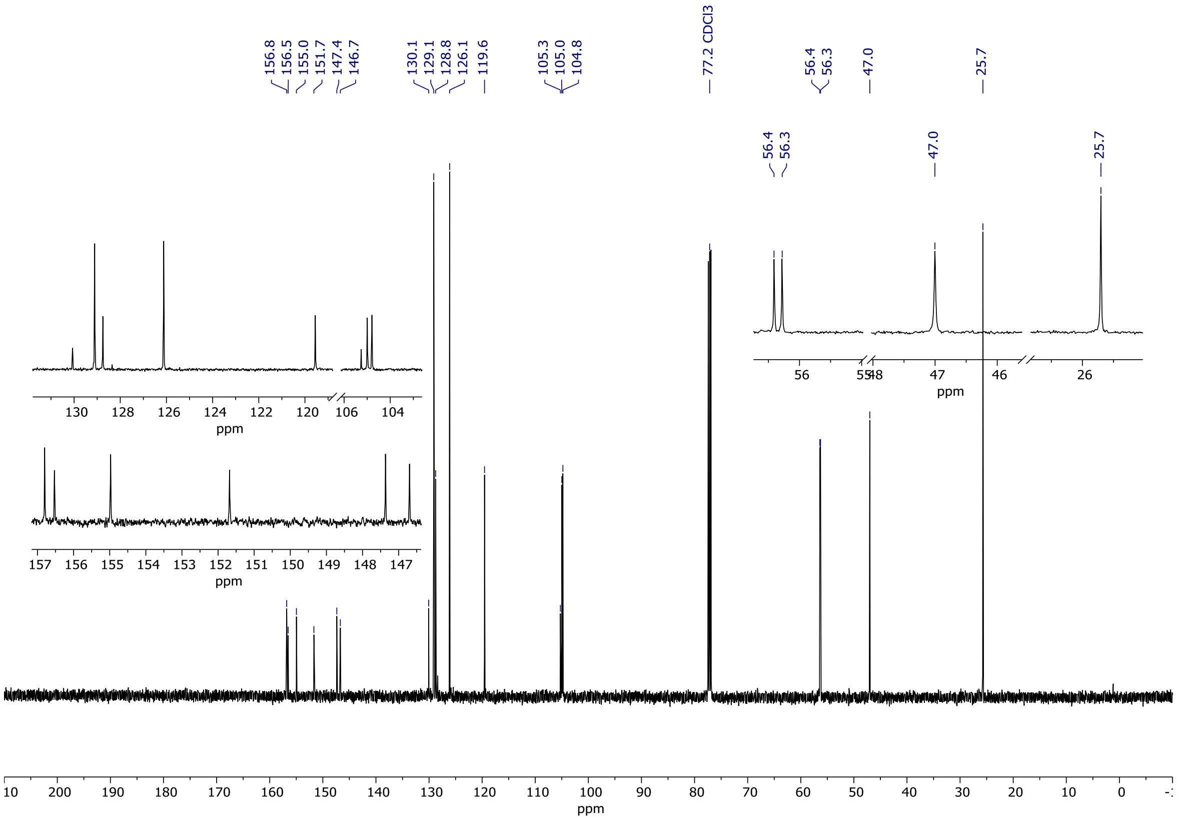

Figure S89: ^{13}C NMR (126 MHz, $CHCl_3$) spectrum.

6,7-Dimethoxy-4-(4-(4-cyanophenyl)-1*H*-1,2,3-triazol-1-yl)-2-(4-methylpiperazin-1-yl)quinazoline (**20b**)

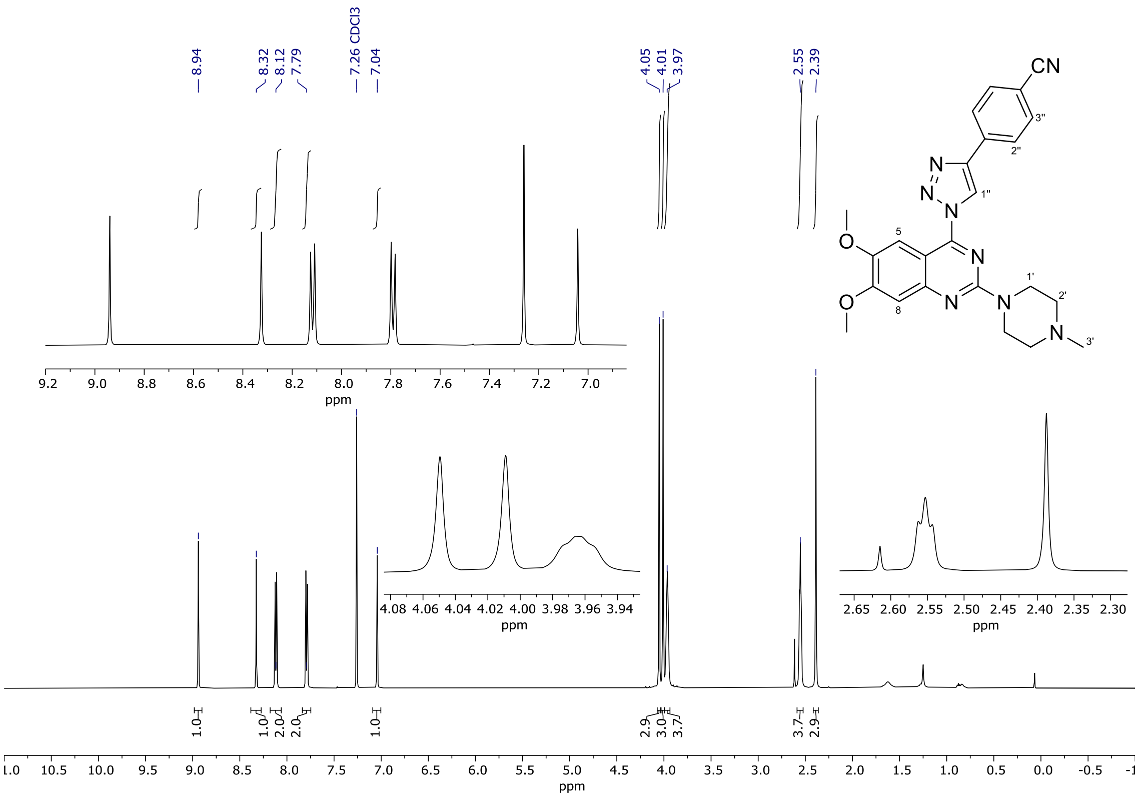

Figure S90: ^{1}H NMR (500 MHz, $CDCl_3$) spectrum.

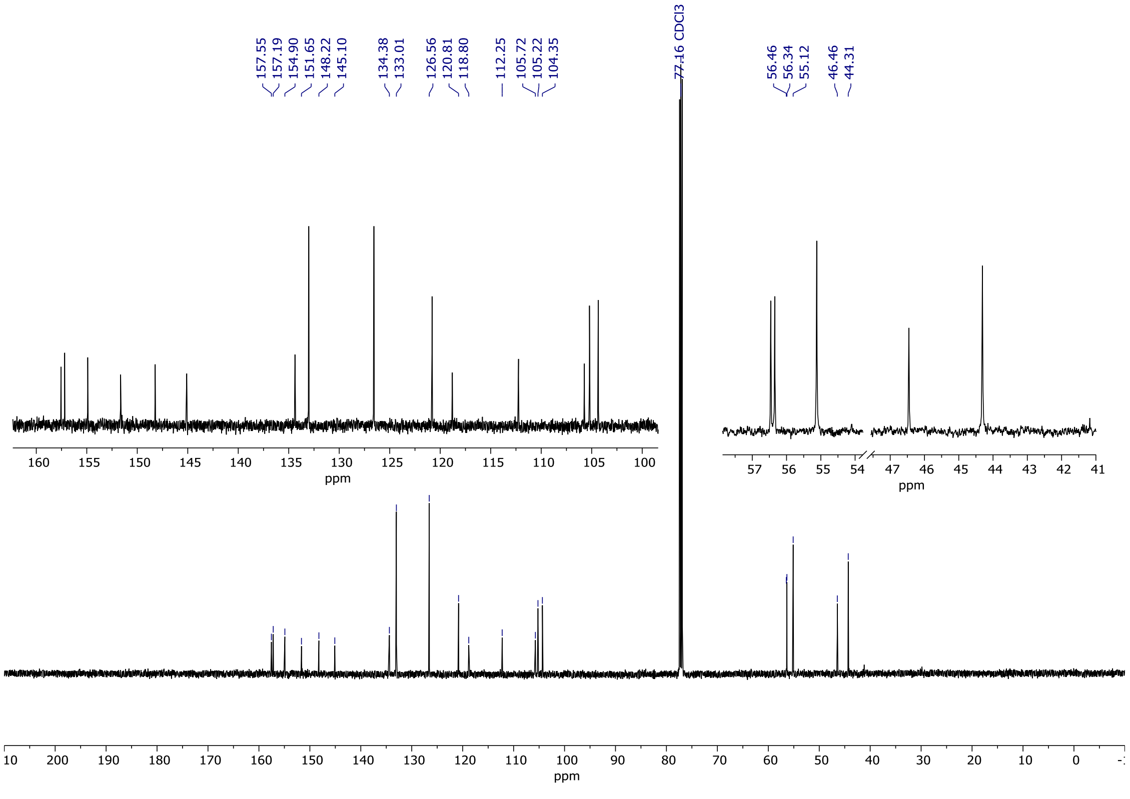

Figure S91: ^{13}C NMR (126 MHz, $CHCl_3$) spectrum.

6,7-Dimethoxy-2-(4-methylpiperazin-1-yl)-4-[(triphenylphosphoronylidene)amino] quinazoline (**21**)

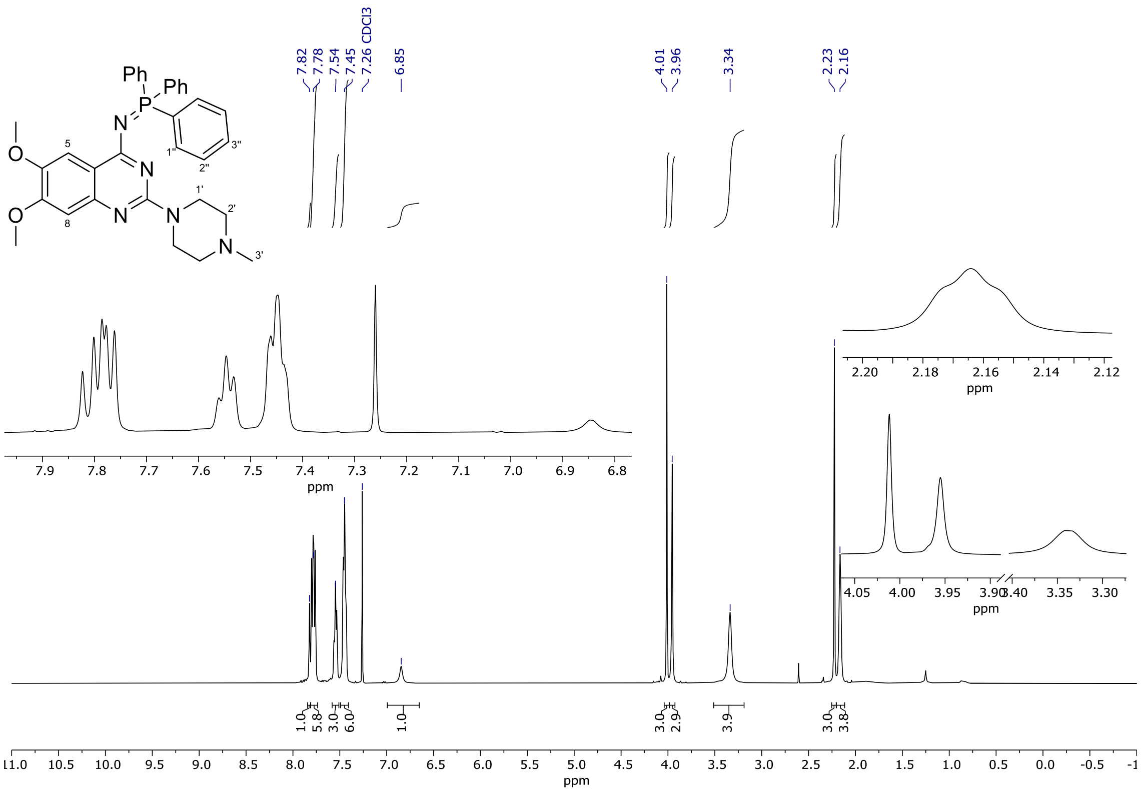

Figure S92: ^{1}H NMR (500 MHz, $CHCl_3$) spectrum.

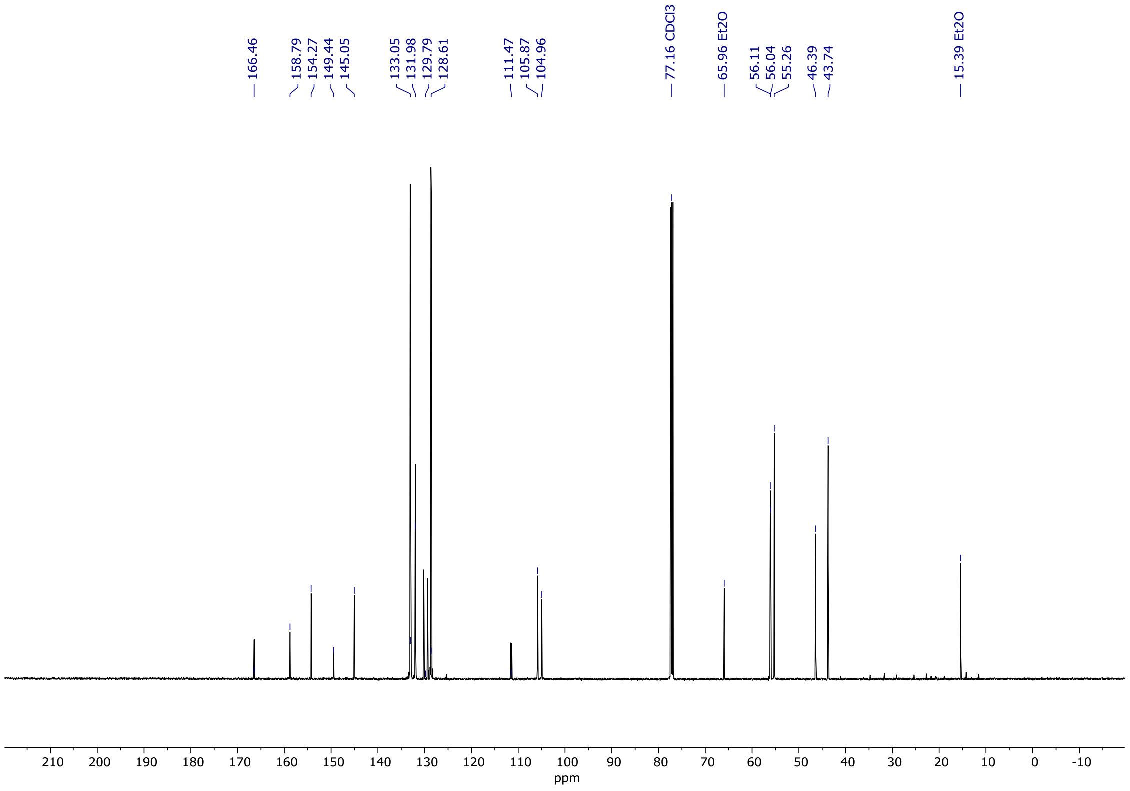

Figure S93: ^{13}C NMR (126 MHz, $CHCl_3$) spectrum.

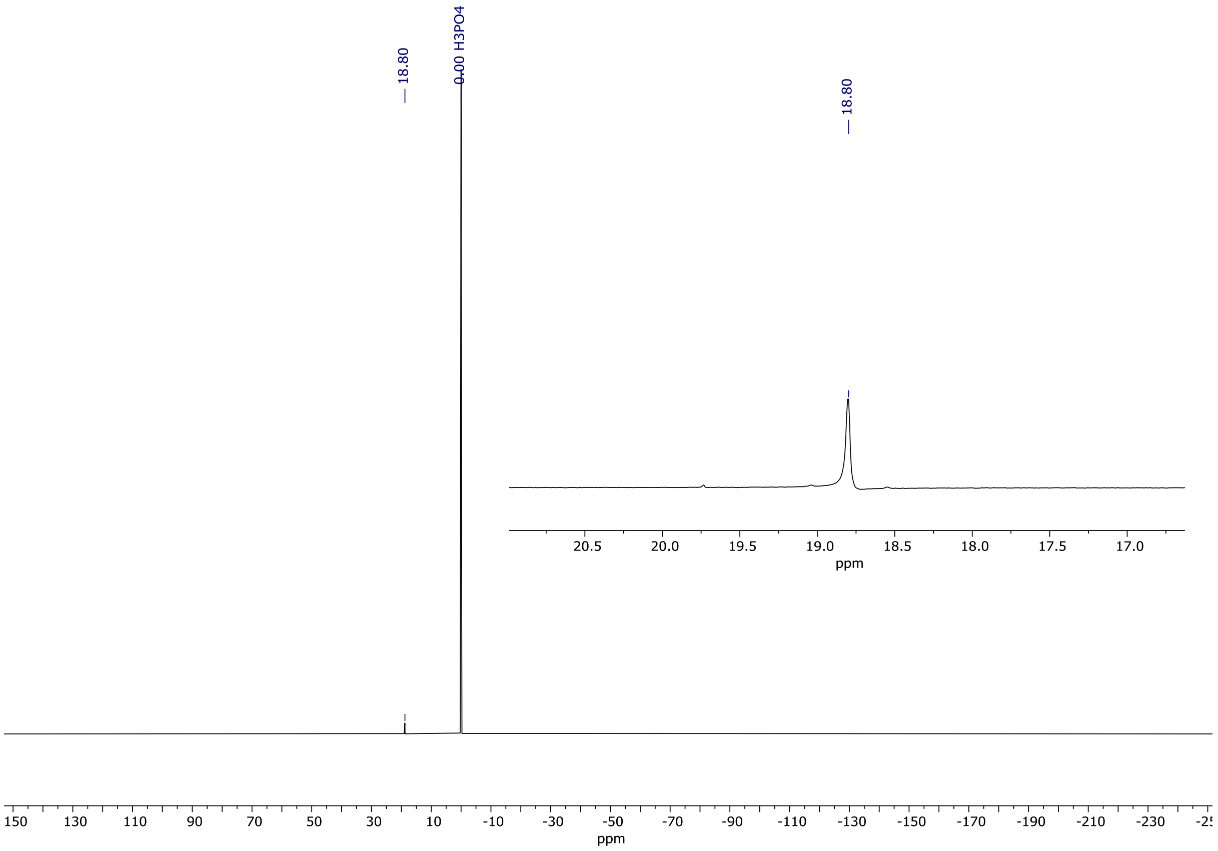

Figure S94: ^{31}P NMR (202 MHz, $CHCl_3$) spectrum with 85% H_3PO_4 external standard.

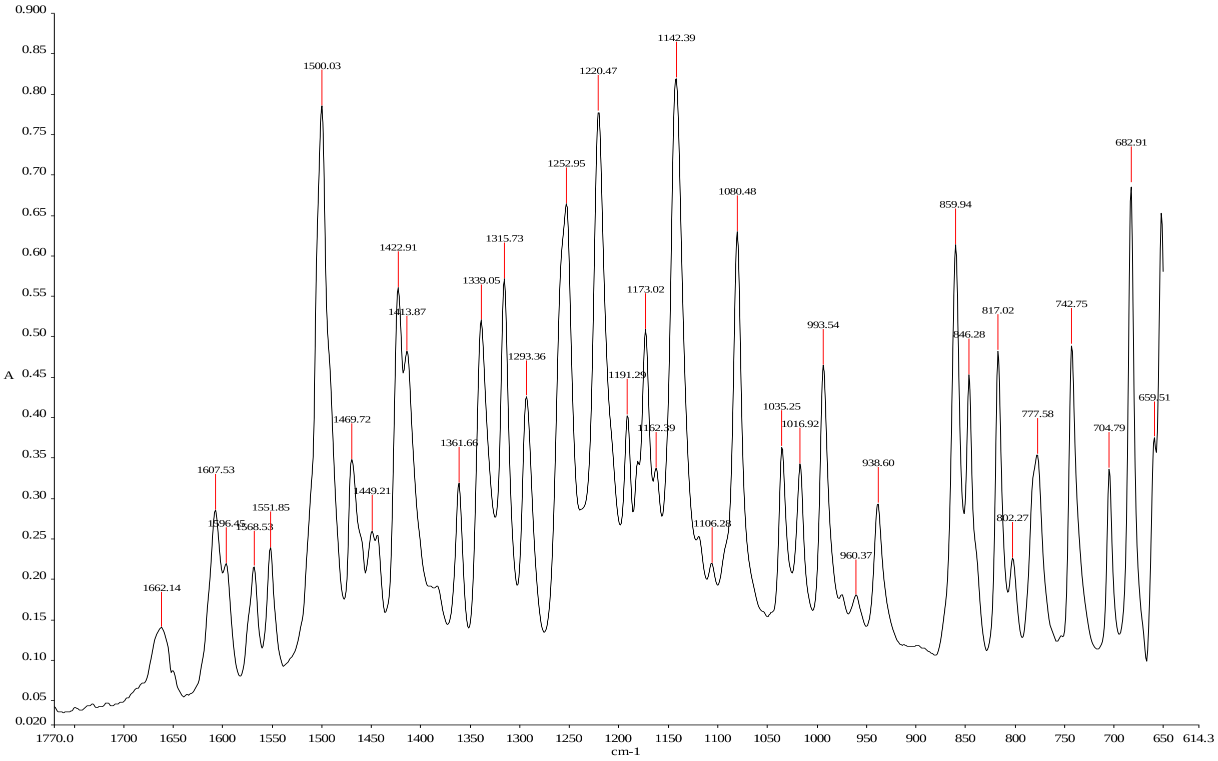

Figure S95: IR (KBr) ν (cm^{-1}) spectra of compound **12a**.

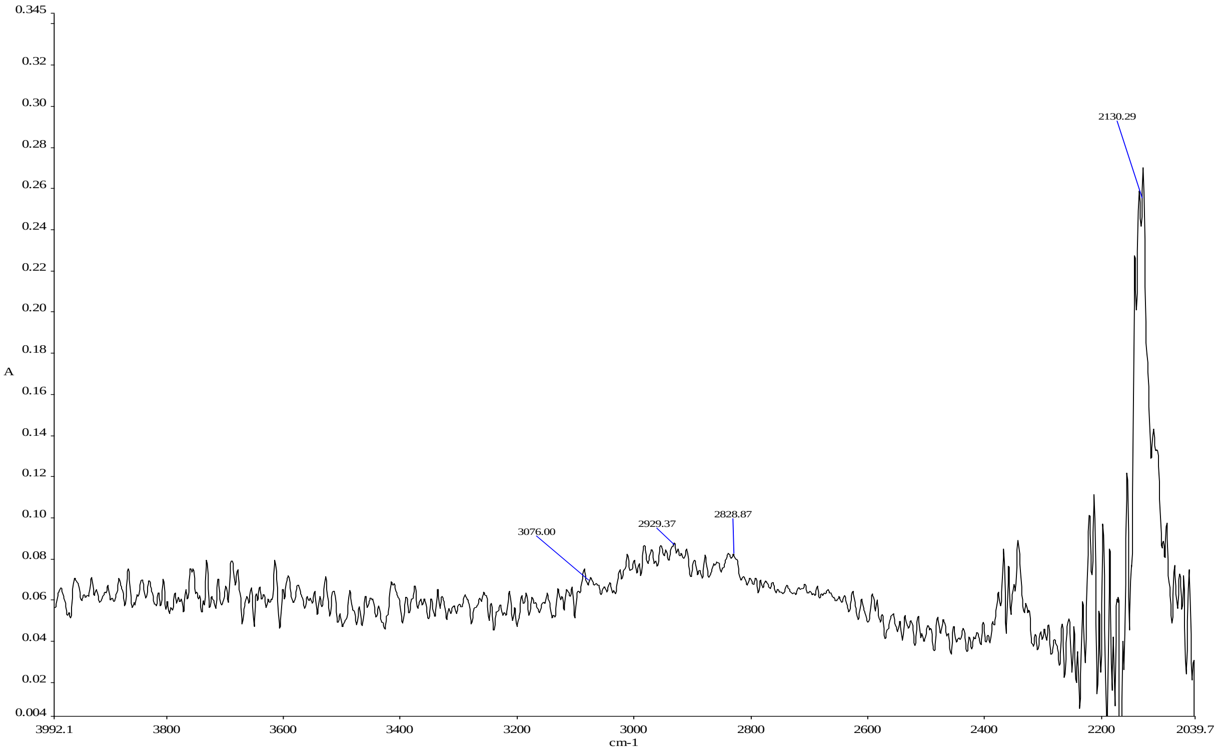

Figure S96: IR (KBr) ν (cm^{-1}) spectra of compound **12a**.

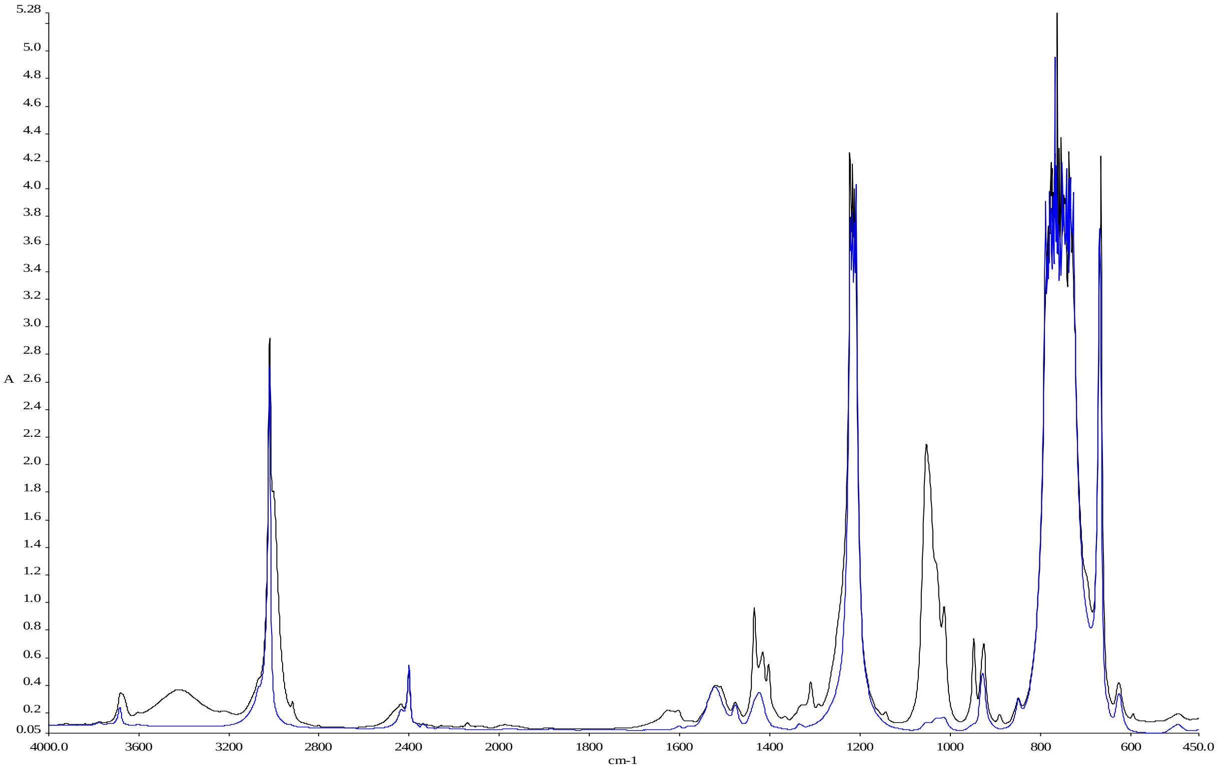

Figure S97: IR ($CHCl_3$ in cuvette) ν (cm^{-1}) spectra of compound **12a**. $CHCl_3$ spectra in blue. Compound **12a** solution in $CHCl_3$ in black.

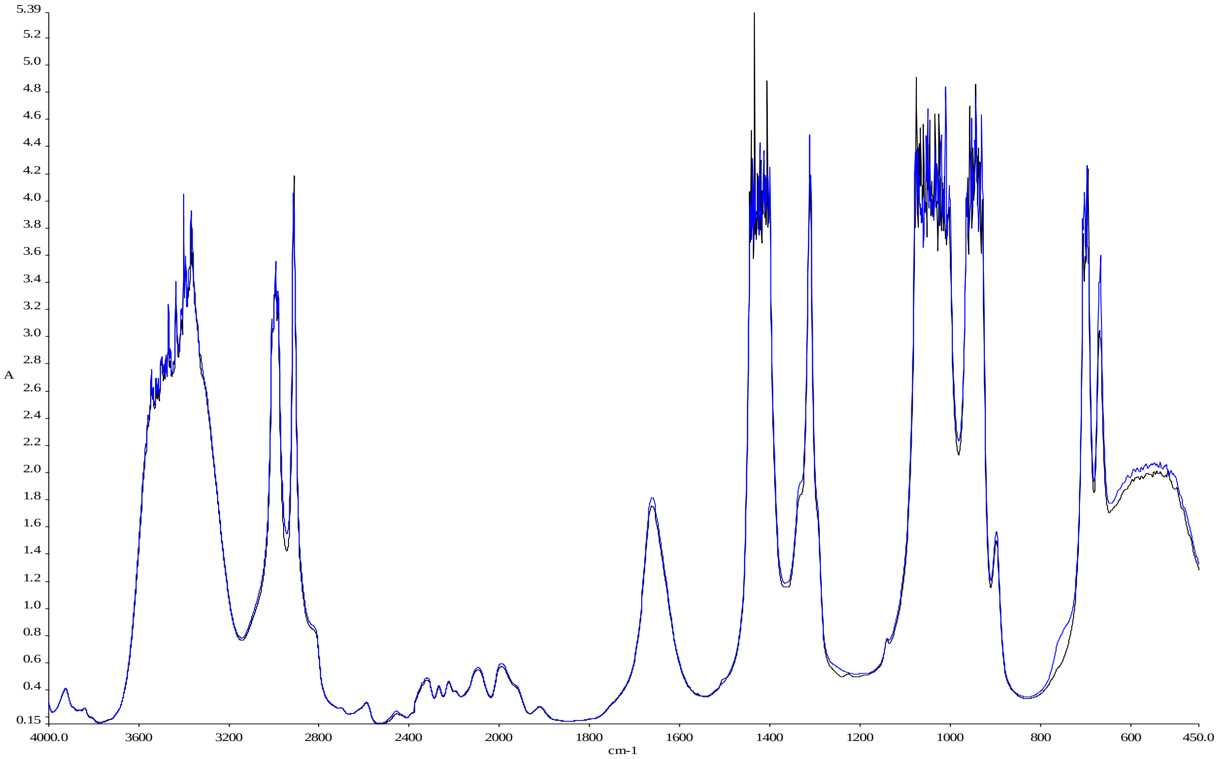

Figure S98: IR (DMSO in cuvette) ν (cm^{-1}) spectra of compound **12a**. DMSO spectra in blue. Compound **12a** solution in DMSO in black.

X-ray crystallography data

4-Azido-6,7-dimethoxy-2-tosylquinazoline (12a)

CCDC-2312750

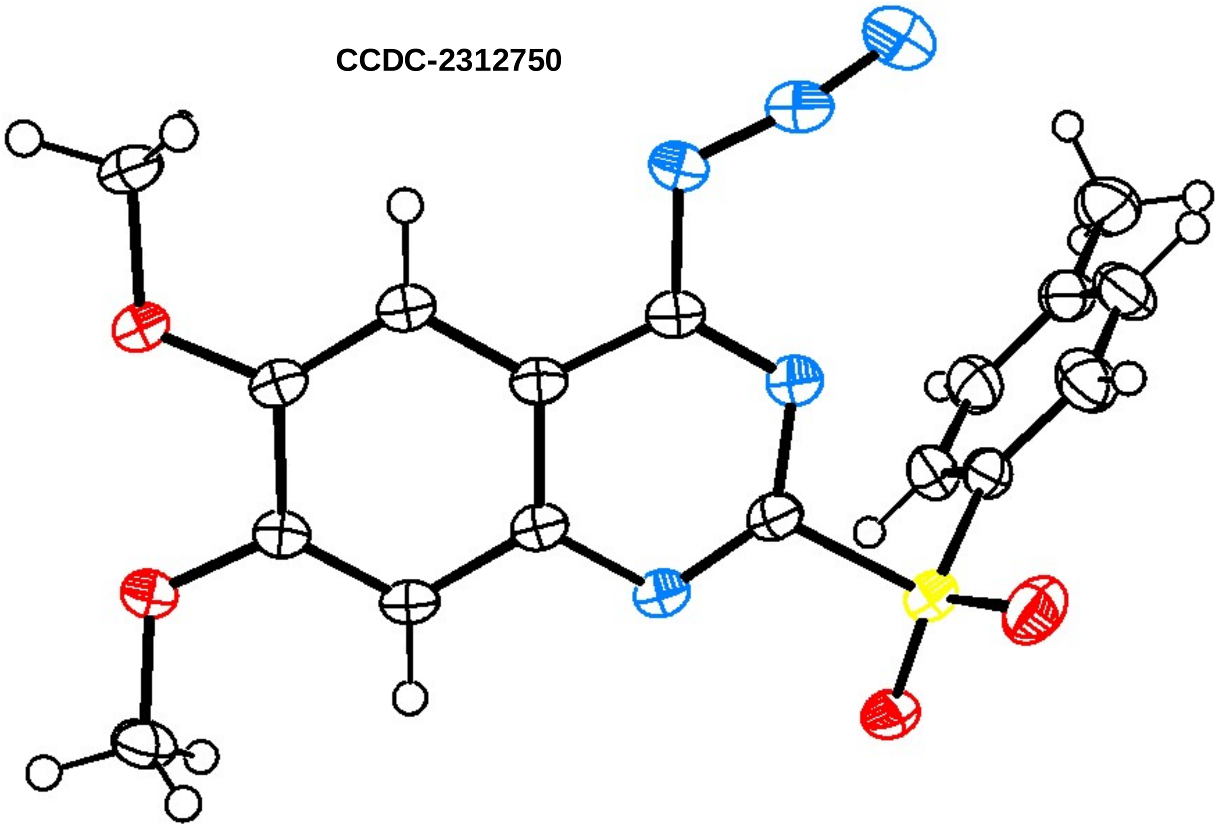

Figure S99. ORTEP plot for compound **12a**.

Crystal data, data collection and structure refinement details are summarized in Table 1.

Table S1. Experimental details.

**Crystal data**	
Chemical formula	$C_{17}H_{15}N_5O_4S$
M_r	385.40
Crystal system, space group	Triclinic, *P*-1
Temperature (K)	150
a, *b*, *c* (Å)	6.9122 (2), 8.46912 (17), 15.3541 (7)
α, β, γ (°)	91.186 (3), 102.063 (3), 99.164 (2)
V (Å^3)	866.41 (5)
Z	2
Radiation type	Cu *K*α
μ (mm^{-1})	1.98
Crystal size (mm)	0.06 × 0.05 × 0.04
**Data collection**	
Diffractometer	XtaLAB Synergy, Dualflex, HyPix
Absorption correction	Multi-scan *CrysAlis PRO* 1.171.41.123a (Rigaku Oxford Diffraction, 2022) Empirical absorption correction using spherical harmonics, implemented in SCALE3 ABSPACK scaling algorithm.
T_{min}, T_{max}	0.823, 1.000
No. of measured, independent and observed [$I > 2\sigma(I)$] reflections	15849, 3481, 3183
R_{int}	0.034
$(\sin \theta/\lambda)_{max}$ (Å^{-1})	0.632
**Refinement**	

$R[F^2 > 2\sigma(F^2)]$, $wR(F^2)$, S	0.036, 0.105, 1.08
No. of reflections	3481
No. of parameters	248
No. of restraints	6
H-atom treatment	H-atom parameters constrained
$\Delta\rho_{max}$, $\Delta\rho_{min}$ (e Å^{-3})	0.32, -0.45

References

(1) Horn, A.; Kazmaier, U. *Eur. J. Org. Chem.* **2018**, *2018*, 2531–2536. doi:10.1002/ejoc.201701645

(2) Nguyen, V. D.; Nguyen, V. T.; Haug, G. C.; Dang, H. T.; Arman, H. D.; Ermler, W. C.; Larionov, O. V. *ACS Catal.* **2019**, *9*, 4015–4024. doi:10.1021/acscatal.9b00464

(3) Joshi, Shreerang V.; Soni, Mukesh N.; Fulwala, Ketan M.; Jalani, Hitesh B.; Prajapati, K. K. Process for preparing anhydrous terazosin hydrochloride. IN2004MU01090, April 27, 2007.

(4) Schwartz, E.; Mendelovici, M.; Gershon, N. Process for the preparation of terazosin hydrochloride dihydrate. US6248888B1, June 19, 2001.

(5) Bottini, A.; De, S. K.; Wu, B.; Tang, C.; Varani, G.; Pellecchia, M. *Chem. Biol. Drug Des.* **2015**, *86*, 663–673. doi:10.1111/cbdd.12534

(6) Honkanen, E.; Pippuri, A.; Kairisalo, P.; Thaler, H.; Koivisto, M.; Tuomi, S. *J. Heterocycl. Chem*. **1980**, *17*, 797–798. doi:10.1002/jhet.5570170436

(7) Petty, A.; Idippily, N.; Bobba, V.; Geldenhuys, W. J.; Zhong, B.; Su, B.; Wang, B. *Eur. J. Med. Chem.* **2018**, *143*, 1261–1276. doi:10.1016/j.ejmech.2017.10.026
