# Supplementary material for: Regioselective quinazoline C2 modifications through the azide–tetrazole tautomeric equilibrium
Source: Beilstein J Org Chem. 2024 Mar 28;20:675–83. doi: 10.3762/bjoc.20.61 (PMC10999978; doi:10.3762/bjoc.20.61)

BEILSTEIN JOURNAL OF ORGANIC CHEMISTRY

# Supporting Information

for

# Regioselective quinazoline C2 modifications through the azide–tetrazole tautomeric equilibrium

Dāgs Dāvis Līpiņš, Andris Jeminejs, Una Ušacka, Anatoly Mishnev, Māris Turks and Irina Novosjolova

*Beilstein J. Org. Chem.* **2024,** *20,* 675–683. doi:10.3762/bjoc.20.61

# Checkcif for compound 12a

License and Terms: This is a supporting information file under the terms of the Creative Commons Attribution License (https://creativecommons.org/licenses/by/4.0). Please note that the reuse, redistribution and reproduction in particular requires that the author(s) and source are credited and that individual graphics may be subject to special legal provisions.
The license is subject to the *Beilstein Journal of Organic Chemistry* terms and conditions: (https://www.beilstein-journals.org/bjoc/terms)

# checkCIF/PLATON report

Structure factors have been supplied for datablock(s) dal_195

THIS REPORT IS FOR GUIDANCE ONLY. IF USED AS PART OF A REVIEW PROCEDURE FOR PUBLICATION, IT SHOULD NOT REPLACE THE EXPERTISE OF AN EXPERIENCED CRYSTALLOGRAPHIC REFEREE.

No syntax errors found. CIF dictionary Interpreting this report

# Datablock: dal_195

---

```
Bond precision:   C-C = 0.0022 A           Wavelength=1.54184

Cell:           a=6.9122(2)        b=8.46912(17)      c=15.3541(7)
                alpha=91.186(3)    beta=102.063(3)    gamma=99.164(2)
Temperature:    150 K

                 Calculated                   Reported
Volume           866.41(5)                    866.41(5)
Space group      P -1                         P -1
Hall group       -P 1                         -P 1
Moiety formula   C17 H15 N5 O4 S              C17 H15 N5 O4 S
Sum formula      C17 H15 N5 O4 S              C17 H15 N5 O4 S
Mr               385.40                       385.40
Dx,g cm-3        1.477                        1.477
Z                2                            2
Mu (mm-1)        1.983                        1.983
F000             400.0                        400.0
F000'            401.90
h,k,lmax                                      8,10,19
Nref                                          3481
Tmin,Tmax        0.888,0.924                  0.823,1.000
Tmin'            0.888

Correction method= # Reported T Limits: Tmin=0.823 Tmax=1.000
AbsCorr = MULTI-SCAN

Data completeness=                Theta(max)= 77.052

R(reflections)= 0.0358( 3183)                          wR2(reflections)=
                                                       0.1051( 3481)

S = 1.083                     Npar= 248
```

The following ALERTS were generated. Each ALERT has the format
**test-name_ALERT_alert-type_alert-level.**
Click on the hyperlinks for more details of the test.

## Alert level B

```
PLAT230_ALERT_2_B Hirshfeld Test Diff for    N18      --N19      .       10.5 s.u.
```

**Author Response: Large differences in anisotropic displacement parameters along chemical bond N18-N19 may be related to contamination of these parameters with other unresolved effects.**

## Alert level C

```
PLAT031_ALERT_4_C Refined Extinction Parameter Within Range of ...      2.600 Sigma
PLAT911_ALERT_3_C Missing FCF Refl Between Thmin & STh/L=    0.600          6 Report
                4 -5  8,   3  4  9,  -7  5 10,  -7  4 11,  -7  3 12,  -6  5 12,
```

## Alert level G

```
PLAT003_ALERT_2_G Number of Uiso or Uij Restrained non-H Atoms ...          2 Report
PLAT178_ALERT_4_G The CIF-Embedded .res File Contains SIMU Records          1 Report
PLAT860_ALERT_3_G Number of Least-Squares Restraints ............           6 Note
PLAT883_ALERT_1_G No Info/Value for _atom_sites_solution_primary .     Please Do !
PLAT899_ALERT_4_G SHELXL2018 is Deprecated and Succeeded by SHELXL     2019/3 Note
PLAT912_ALERT_4_G Missing # of FCF Reflections Above STh/L=  0.600        183 Note
PLAT941_ALERT_3_G Average HKL Measurement Multiplicity ..........         4.6 Low
PLAT978_ALERT_2_G Number C-C Bonds with Positive Residual Density.          8 Info
```

0 **ALERT level A** = Most likely a serious problem - resolve or explain
1 **ALERT level B** = A potentially serious problem, consider carefully
2 **ALERT level C** = Check. Ensure it is not caused by an omission or oversight
8 **ALERT level G** = General information/check it is not something unexpected

1 ALERT type 1 CIF construction/syntax error, inconsistent or missing data
3 ALERT type 2 Indicator that the structure model may be wrong or deficient
3 ALERT type 3 Indicator that the structure quality may be low
4 ALERT type 4 Improvement, methodology, query or suggestion
0 ALERT type 5 Informative message, check

It is advisable to attempt to resolve as many as possible of the alerts in all categories. Often the minor alerts point to easily fixed oversights, errors and omissions in your CIF or refinement strategy, so attention to these fine details can be worthwhile. In order to resolve some of the more serious problems it may be necessary to carry out additional measurements or structure refinements. However, the purpose of your study may justify the reported deviations and the more serious of these should normally be commented upon in the discussion or experimental section of a paper or in the "special_details" fields of the CIF. checkCIF was carefully designed to identify outliers and unusual parameters, but every test has its limitations and alerts that are not important in a particular case may appear. Conversely, the absence of alerts does not guarantee there are no aspects of the results needing attention. It is up to the individual to critically assess their own results and, if necessary, seek expert advice.

**Publication of your CIF in IUCr journals**

A basic structural check has been run on your CIF. These basic checks will be run on all CIFs submitted for publication in IUCr journals (*Acta Crystallographica*, *Journal of Applied Crystallography*, *Journal of Synchrotron Radiation*); however, if you intend to submit to *Acta Crystallographica Section C* or *E* or *IUCrData*, you should make sure that full publication checks are run on the final version of your CIF prior to submission.

**Publication of your CIF in other journals**

Please refer to the *Notes for Authors* of the relevant journal for any special instructions relating to CIF submission.

---

**PLATON version of 29/11/2023; check.def file version of 14/09/2023**

**Datablock dal_195** - ellipsoid plot

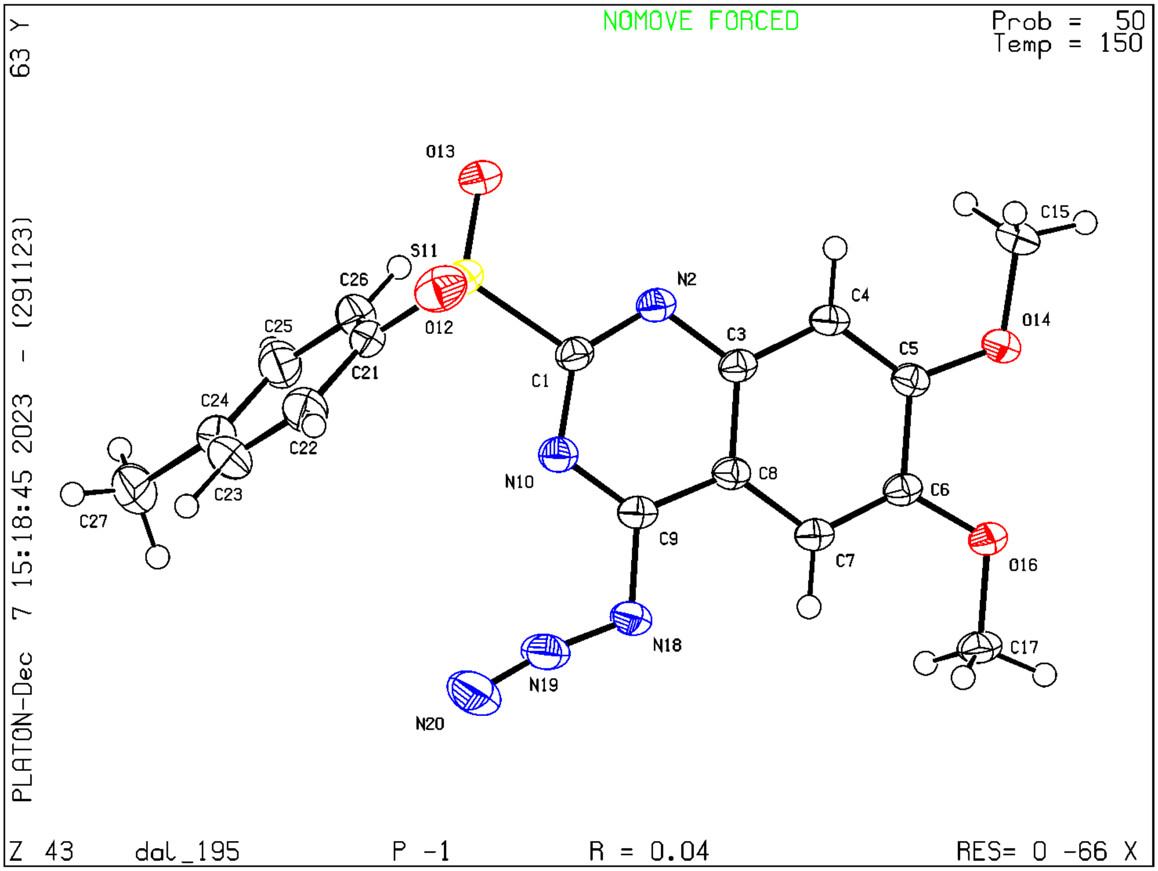

Supplement: File 2 — Checkcif for compound 12a. [file Beilstein_J_Org_Chem-20-675-s002.pdf]
